# STUB1-induced polyubiquitination of SIK3 in alveolar type 2 epithelial cells alleviates severity and outcomes of acute lung injury

**DOI:** 10.1038/s41419-026-08822-x

**Published:** 2026-05-04

**Authors:** Feng Tian, Nianlin Xie, Daixing Zhong, Yunfeng Ni, Binghua Zhang, Xiaohua Liang, Jun Ma, Xiaokang Gong, Zhuochen Sun, Jie Zhao, Tao Jiang, Wei Li

**Affiliations:** 1 Department of Thoracic Surgery, Tangdu Hospital, Air Force Medical University (formerly known as Fourth Military Medical University), Xi’an, PR China; 2 Department of Pulmonary and Critical Care Medicine, Air Force 986 Hospital, Air Force Medical University (formerly known as Fourth Military Medical University), Xi’an, PR China; 3 Department of Thoracic Surgery, Air Force 986 Hospital, Air Force Medical University (formerly known as Fourth Military Medical University), Xi’an, PR China; 4 Department of Human Anatomy, Histology and Embryology, Basic Medical Science Academy, Air Force Medical University (formerly known as Fourth Military Medical University), Xi’an, PR China

**Keywords:** Cell death and immune response, Respiratory tract diseases

## Abstract

Disruption in alveolar type 2 epithelial cells (AT2s) homeostasis by oxidative stress plays an essential role in the pathogenesis of acute lung injury (ALI). However, significant discrepancies remain in understanding the mechanisms for AT2 as a main target for reactive oxygen species (ROS). Herein, we show that STUB1, an E3 ligase involved in protein homeostasis, was dominantly expressed in AT2s. Mild levels of ROS potentiated Nrf2-mediated transactivation of the *STUB1* gene via activation of the ERK signaling, whereas high levels of ROS compromised STUB1 expression by dampening STUB1 protein half-life. Ablation of *Stub1* in AT2s caused failure in conferring K63-mediated nonproteolytic polyubiquitination of SIK3 (salt-inducible kinase 3), which in turn abrogated CRTC2 (CREB-regulated transcription co-activator 2) substrate binding for SIK3 and thereby triggered CREB signaling-mediated proinflammatory phenotypes. Consequently, disruption in STUB1/SIK3 signaling aggravated lung edema, augmented inflammatory infiltrate, and increased AT2 apoptosis in vivo. Mice lacking STUB1 also demonstrated increased susceptibility to ischemia-reperfusion and overventilation-induced lung injury. These findings unambiguously uncover STUB1 as a critical post-translational regulator of ALI severity and outcomes.

## Introduction

Acute lung injury (ALI), as well as its most severe form acute respiratory distress syndrome (ARDS), is initiated by either infectious or noninfectious inflammatory stimuli and represents a spectrum of common life-threatening and rapid respiratory system deterioration that are associated with diffuse alveolar damage, excessive pulmonary inflammation and apoptosis of alveolar epithelial cells (ATs). Persistent and repetitive injury by ALI can eventually lead to pulmonary fibrosis. ALI is associated with 30–40% mortality with current standard of care and is responsible for approximately 79,000 deaths in US yearly which would make it 1 of the top 8 causes of death in 2018 [1]. A prospective 12-month clinical study in Shanghai showed that incidence of ARDS for patients >15 years is 2% in China, with a mortality rate of 70% [2]. In essence, ALI/ARDS reflects severe injury causing dysfunction and compromise of the barrier properties of the pulmonary endothelium and epithelium as a consequence of an unregulated inflammatory response [3]. The damaged alveolar endothelial and epithelial integrity may in part be caused by increased oxidative stress originated from many potential sources (e.g. itinerant and resident leukocytes, parenchymal cells, circulating oxidant-generating enzyme and inhaled gases with high concentrations of oxygen during mechanical ventilation), which results in extensive damage in membrane, cytosolic, and nuclear lipids and proteins [4]. Other signaling pathways have been proposed to underlie early loss of lung endothelial and epithelial integrity, including activation of the TLR4/NF-κB signaling pathway [5], aberrant activation of macrophages and local proinflammatory signaling [6] and high-mobility group box-1 (HMGB1)-associated autophagy [7]. Nevertheless, the exact mechanisms by which alveolar endothelium is impaired in ALI remain elusive.

The *STUB1* gene (STIP1 homology and U-box containing protein 1), encoding the CHIP (carboxyl terminus of Hsp70-interacting protein) functioning as a chaperone-dependent E3 ubiquitin ligase, is fundamentally involved in protein quality control that manages the turnover of multiple substrates. STUB1 is widely expressed throughout whole body depending on its targeting substrates [8]. Although originally named for its ability to interact with heat shock protein 70 (HSP70), it is now clear that STUB1 can ubiquitinate a broad range of important molecules, thus exhibiting widespread functions in various physiological process, such as aging [9], autophagy [10], male reproduction [11], neurodegenerative disorders [12] and bone remodeling [13]. Moreover, emerging evidence has shown that STUB1 can destabilize IFNγ-receptor complex to suppress tumor IFNγ signaling, thus exerting tumor-suppressive functions [14]. Similarly in macrophages, STUB1 binds with the PPARγ protein to mediate PPARγ polyubiquitination and degradation [15]. The last two observations suggest that STUB1 may regulate immune programs in numerous cell types.

Although the molecular basis underlying STUB1’s actions in various physiological systems remain largely obscure, recurring discovery of STUB1 among the top 1% hits in the genetic screens of the targets needed for immunity, highlight a significant, yet under-appreciated role of STUB1 in regulating immune responses [16]. Based on the canonical role of STUB1 in response to stress stimuli [17], we hypothesize that STUB1 may play a conserved and prominent role in dampening stress triggered by ALI. In this study, using both cell culture and a number of alveolar epithelial-specific genetic knockout (KO) mouse models, we uncovered critical factors regulating the abundance of STUB1 on alveolar type 2 epithelial cells (AT2s). We further mechanistically characterized how STUB1 governed activation of the SIK3 (salt-inducible kinase 3) signaling by regulating its polyubiquitination. Lastly, we assessed the context-dependent impact of STUB1 deficiency on lung injury, as well as STUB1-dependent SIK3 signaling for the response to ALI treatment in vivo.

## Results

### Upregulation of pulmonary STUB1 expression following LPS-induced ALI

**Fig. 1:**
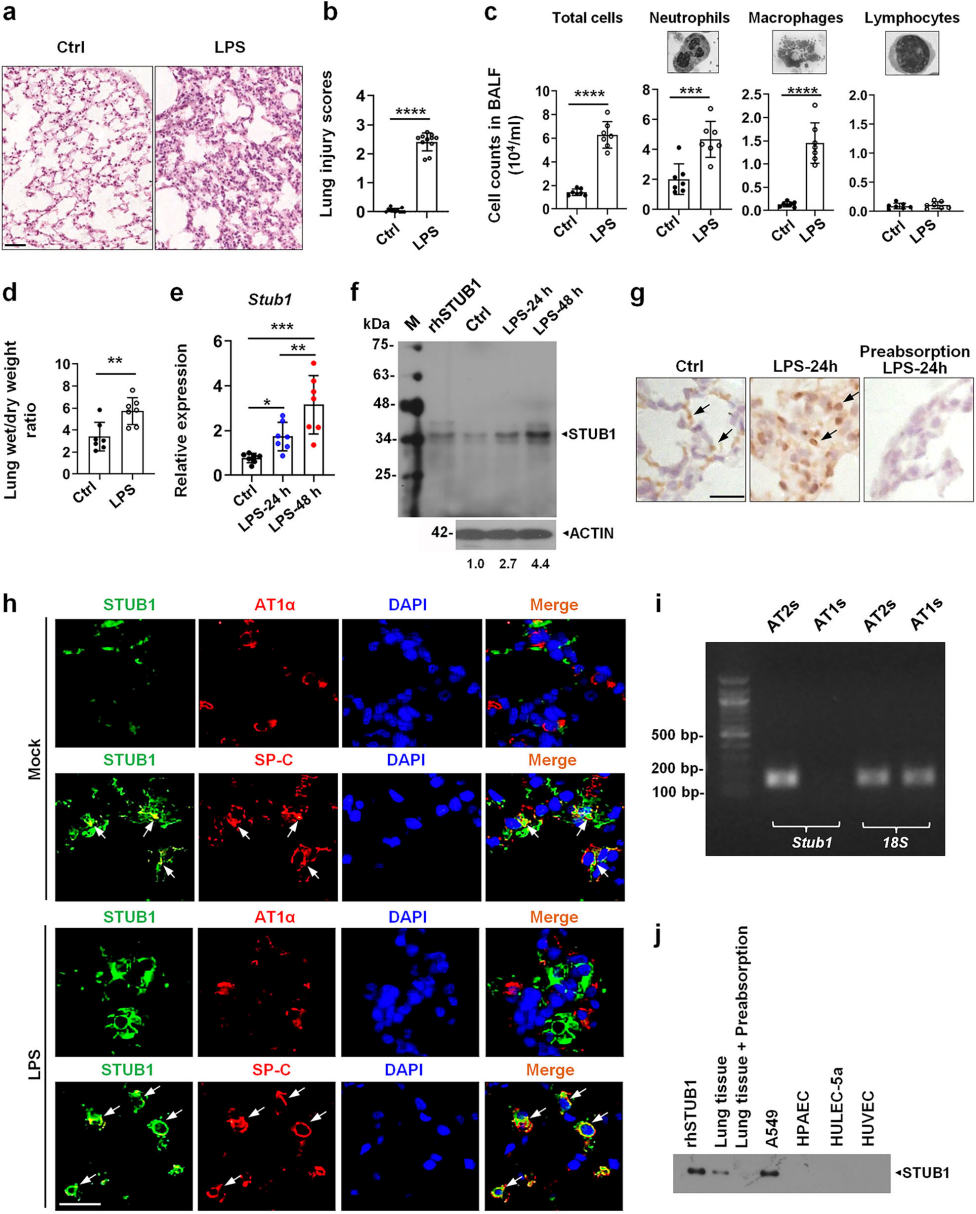
Specific expression of STUB1 in alveolar type 2 epithelial cells (AT2s) is induced by acute lung injury (ALI). **a** Haematoxylin and eosin (H&E) staining of lung sections derived from sham control or lipopolysaccharide (LPS)-challenged mice at 24 h post-treatment. Bar=25 μm (**b**) Morphometric analyses of lung by lung injury scores on a 0‒4-point scale in all groups as in (**a**), *n* = 11 in each group, ****P < 0.0001 (*Student’s t* test). **c** Cell counts (including total cells, neutrophils, macrophages, lymphocytes) in bronchoalveolar lavage fluid (BALF) 24 h after intratracheal administration of PBS or LPS (5 μg/g), *n* = 7/group, ***P < 0.001 and ****P < 0.0001 (*Student’s t* test). All samples were biologically independent, and three or more independent experiments were performed. All quantitative data are shown as mean ± S.D. and analyzed with a 95% confidence interval. **d** Lung oedema was measured as the wet/dry weight ratio of the excised lung from mice 24 h after intratracheal administration of PBS or LPS, *n* = 7/group, **P < 0.01. **e** Relative expression levels of *Stub1* mRNA in the lung were assessed by qPCR at 24 h or 48 h after intratracheal administration of PBS or LPS, *n* = 7/group, *P < 0.05, **P < 0.01 and ***P < 0.001 (one-way ANOVA followed by Tukey’s post-hoc test and each bar represents the mean ± SD of results from three experiments using different tissue samples). **f** Immunoblotting analysis of STUB1 along the pathogenesis of ALI. β-ACTIN served as a loading control. Densitometric scanning of immunoblots was performed in which the level of a target protein was normalized against the protein level in Ctrl group, which was arbitrarily set at 1. **g** Immunostaining of STUB1 protein in lung sections revealed an increased expression of STUB1 (arrows) at 24 h following LPS challenge. Replacement of the primary antibody with a preabsorbed IgG totally abolished the positive staining, confirming the specificity of the assay. The pre-absorbed serum was prepared by incubating the anti-STUB1 antibody with the STUB1 peptide antigen (final concentration of peptide 5.0 μg/ml) for 12 h at 4°C while rotating. Bar=25 μm (**h**) Double immunofluorescence labeling suggests STUB1 was predominantly expressed in AT2s, as indicated by the overlapping staining of STUB1 with SP-C (arrows), a AT2 marker but not with AT1α (a AT1 marker). Bar=25 μm (**i**) Isolation and purification of murine alveolar epithelial cells (ATs) was carried out as described in Supplementary information, followed by RT-PCR analysis of *Stub1* expression in different cell types. *18S* was used as a loading control. **j** Immunoblotting of STUB1 with lysates from mouse lung tissues, and human lung adenocarcinoma A549 cells, human pulmonary artery endothelial cells (HPAEC), human lung microvascular endothelial cells (HULEC-5a) and human umbilical vein endothelial cells (HUVEC; 50 μg per lane). rhSTUB1 (100 ng) served as a positive control for the immunoblotting. The positive signals were completely abolished when samples were incubated with preabsorbed primary antibody (*lane*: lung tissue + Preabsorption), verifying the specificity of the assay.

To explore a potential involvement of STUB1 in ALI, we first generated a murine model of pulmonary damage induced by lipopolysaccharide (LPS) via intratracheal instillation. LPS challenge for 24 h resulted in significant changes in pulmonary histology, including interstitial tissue edema, capillary congestion and inflammatory infiltrate (Fig. 1a). Consistently, lung injury scores equal to or above 3 ( ≥ 2.5 if rapid deterioration [18]) were frequently observed in LPS-treated mice but undetectable in Ctrl mice (Fig. 1b). There was also a significant increase in total cell counts, as well as in total number of neutrophils and macrophages, in bronchoalveolar lavage fluid (BALF) at 24 h in the LPS-treated mice compared to PBS control group, while no changes were observed in total number of BALF lymphocytes between two experimental groups (Fig. 1c). The recruitment of neutrophils and macrophages in LPS-treated wild-type (WT) mice was further validated through qPCR analysis employing specific primers for each cell type, in addition to biochemical assessment of the neutrophil activation biomarker myeloperoxidase (MPO) (Supplementary Fig. 1). Pulmonary edema, measured as a significant increase in lung wet/dry weight ratio, was noticeable in LPS-treated mice but not in Ctrl mice (Fig. 1d). To ask whether STUB1 expression changed during ALI, we performed qPCR and immunoblotting analyses using lung tissue samples. The results demonstrated a progressive increase in *Stub1*/STUB1 expression in lung tissues throughout ALI induction, with the maximum expression level observed in samples at postoperative 48 h (Fig. 1e, f). Subsequent immunohistochemistry consistently showed a more intensive STUB1-positive staining: arrows in Fig. 1g indicate the STUB1-positive alveolar epithelial cells in LPS-treated mice compared to the PBS control group. By contrast, replacement with preabsorbed IgG abolished the STUB1 immunostaining, further confirming the assay specificity (Fig. 1g). The following double immunofluorescence staining revealed that STUB1 was mainly expressed in AT2s, as STUB1 immunostaining was overlapped with SP-C, but not with AT1α, in lung tissue sections from both LPS- and PBS-treated mice (Fig. 1h). The exclusive expression of pulmonary STUB1 in murine AT2s was further confirmed by RT-PCR analysis in primary cultured AT1s and AT2s (Figs. 1i and S2). To further validate the expression pattern of pulmonary STUB1 at the in vitro level, we carried out immunoblotting using cultured adenocarcinoma human type II alveolar epithelial cells (A549), human pulmonary artery endothelial cells (HPAEC), human lung microvascular endothelial cells (HULEC-5a) and human umbilical vein endothelial cells (HUVEC), along with lung tissue samples and recombinant human STUB1 (rhSTUB1, positive control). Clearly, endogenous STUB1 was exclusively expressed in AT2s, but not in AT1s or endothelial cells (Fig. 1j).

### STUB1 deficiency exacerbates LPS-associated ALI and mortality

**Fig. 2:**
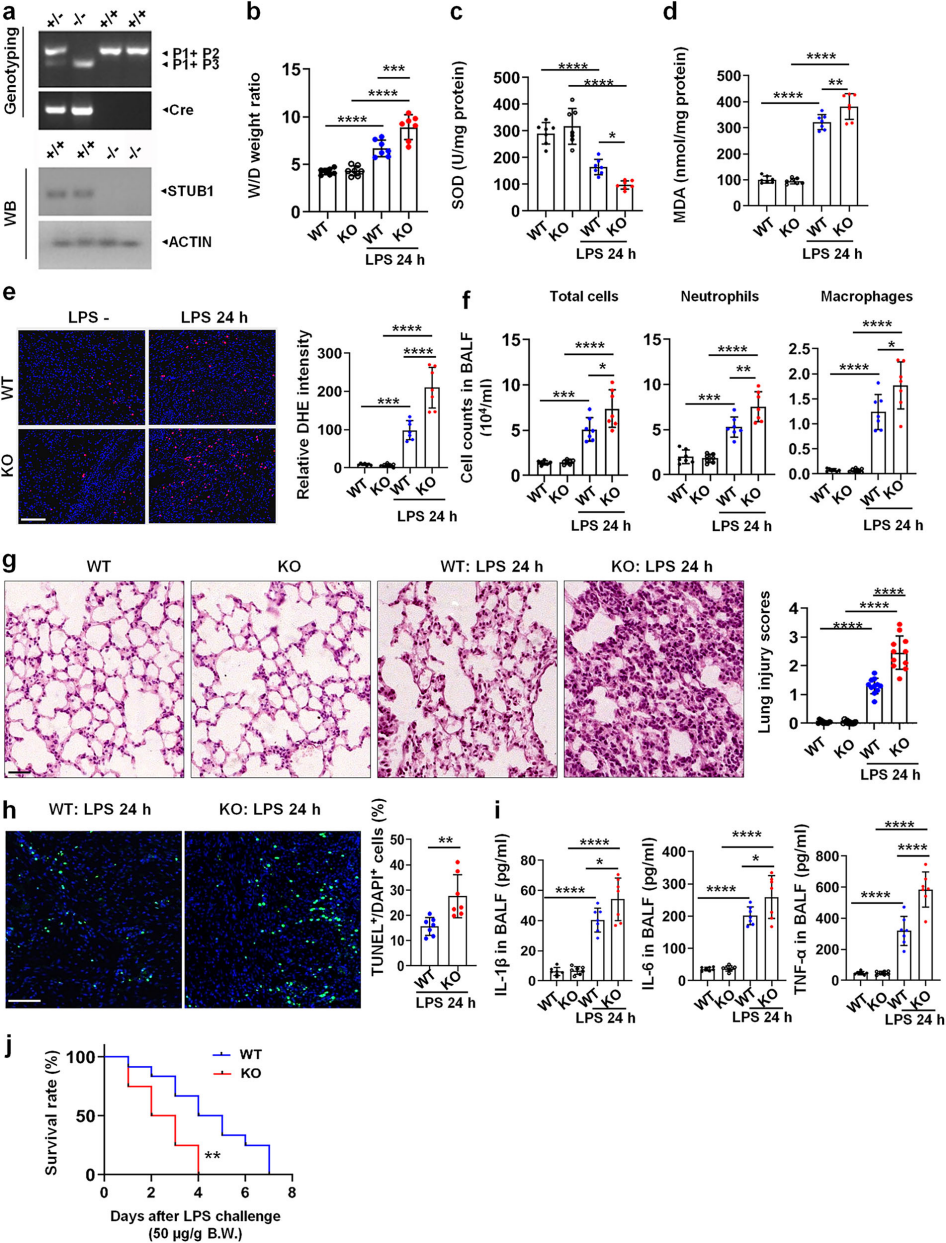
Genetic deletion of *Stub1* augments LPS-induced ALI and mortality. **a** Upper panel, genotyping using genomic DNA samples isolated from tails of 4-week-old pups, was carried out using different primers as described (Reference [11]). Lower panel, immunoblotting analysis confirmed the absence of STUB1 protein in lung samples from 2-month-old KO mice. **b** Lung oedema was measured as the wet/dry weight ratio of the excised lung from WT or KO mice 24 h after intratracheal administration of PBS or LPS, *n* = 7/group, ***P < 0.001, ****P < 0.0001 (two-way ANOVA followed by Tukey’s post-hoc test). **c** The superoxide dismutase (SOD) contents in lung tissues after *Stub1* knock out and/or LPS (sham) challenge were analyzed by spectrophotometry at 450 nm. Data are expressed as units per milligram protein, and the corresponding histograms are shown. *n* = 7/group, *P < 0.05 and ****P < 0.0001 (two-way ANOVA followed by Tukey’s post-hoc test). **d** The malondialdehyde (MDA) contents in lung tissues after *Stub1* knock out and/or LPS (sham) challenge were analyzed by spectrophotometry at 532 nm. Data are expressed as nanomole per milligram protein, and the corresponding histograms are shown. *n* = 7/group, **P < 0.01 and ****P < 0.0001 (two-way ANOVA followed by Tukey’s post-hoc test). **e** Representative images of dihydroethidium (DHE) fluorescent imaging of lung tissue sections from different experimental groups. Right panel, the relative DH signal intensity was calculated and exported as the red 2-OH-E^+^ positive-cells per 10^3^ cells. *n* = 7/group, ****P < 0.0001 (two-way ANOVA followed by Tukey’s post-hoc test). **f** Cell counts (including total cells, neutrophils and macrophages) in BALF **f**rom WT or KO mice 24 h after intratracheal administration of PBS or LPS (5 μg/g), *n* = 7/group, *P < 0.05, **P < 0.01, ***P < 0.001 and ****P < 0.0001 (two-way ANOVA followed by Tukey’s post-hoc test). **g** H&E staining of lung sections derived from sham control or LPS-challenged WT or KO mice at 24 h post-treatment. Bar=25 μm. Right panel, morphometric analyses of lung by lung injury scores on a 0‒4-point scale in all groups, *n* = 11/group, ****P < 0.0001 (two-way ANOVA followed by Tukey’s post-hoc test). **h** Representative 4% paraformaldehyde (PFA)-fixed lung sections stained by TUNEL demonstrated increased apoptosis in KO mice at 24 h after LPS exposure (Bar=25 μm). Right panel, quantification of TUNEL–positive cells per field in LPS-treated WT or KO mice at 24 h after LPS exposure (*n* = 7, **P < 0.01, *Student’s t* test). **i** Levels of IL-1β, IL-6, and TNF-α in BALF from mice 24 h after intratracheal administration of PBS or LPS (5 μg/g) as determined by ELISA, *n* = 7/group, *P < 0.05, **P < 0.01 and ***P < 0.001 (two-way ANOVA followed by Tukey’s post-hoc test). **j** Survival rate following instillations of ALI-inducing lethal LPS (50 μg/g B.W.) in WT or KO mice was assessed by log-rank test (*n* = 12, **P < 0.01, Log-rank (Mantel-Cox) test).

To better understand the biological effects of STUB1, we generated an AT2-specific *Stub1* knockout mouse line (Stub1^fl/fl^/Sftpc-Cre^+/–^, hereafter referred to as KO; while Stub1^+/+^ served as the WT control) using a Cre-loxP conditional KO strategy, which caused a deletion of exons 2 and 3 in the floxed *Stub1* allele. The Stub1^fl/fl^/Sftpc-Cre^+/–^ mice were generated by mating floxed *Stub1* female mice with Sftpc-Cre transgenic male mice [19]. After two generations of mating, Stub1^fl/fl^/Sftpc-Cre^+/–^ male mice and WT littermates were developed. Genotyping of KO mice was identified at the genomic DNA level (non-product for P1-P2 amplification in KO mice, whereas 468 bp for KO mice in P1-P3 products, upper panel in Figs. 2a and S3) [11]. Western blotting confirmed that no STUB1 polypeptide was present in the KO lung (lower panel in Fig. 2a). We then determined the susceptibility of these animals to LPS-induced ALI. The KO mice showed significantly greater W/D weight ratio (Fig. 2b), impaired superoxide dismutase (SOD, Fig. 2c) contents, increased malondialdehyde (MDA, Fig. 2d) and dihydroethidium (DHE) production (Fig. 2e) than WT controls, at 24 h after LPS challenge, affirming aggravation of lung edema formation and oxidative stress-induced DNA damage in the absence of STUB1. Concomitantly, both total cell number and distinct inflammatory cells (neutrophils and macrophages) increased dramatically in lungs from KO mice at 24 h after LPS treatment (Fig. 2f). By H&E (hematoxylin-eosin) staining, we evaluated the pathohistological relevance of these findings. Under resting conditions, no changes in lung histology were observed between KO and WT mice. At 24 h following LPS challenge, however, tissue damage (including alveolar edema, thickening of alveolar walls, inflammatory infiltrates and disruption in alveolar structure), as well as the expression levels of the neutrophil activation biomarker MPO, was more evident in KO mice (Figs. 2g and S4), and meanwhile, STUB1 deficiency also significantly augmented LPS-elicited apoptosis of alveolar epithelial cells 24 h post exposure (Fig. 2h). In accordance with these pathological changes, expression levels of several key pro-inflammatory cytokines (e.g. IL-1β, IL-6 and TNF-α) were also markedly upregulated in BALF from KO mice when exposed to LPS (Fig. 2i). To evaluate the effect of inflammation severity, we subjected mice to intratracheal LPS at lethal (50 μg/g) doses [20]. Compared with WT controls, the KO mice had a lower survival rate following lethal LPS challenge (Fig. 2j). Notably, parallel experiments were conducted to exclude possible effects of Cre expression in the lung (Supplementary Fig. S5). Thus, adequacy of the AT2 STUB1 expression appeared to be a determinant of survival during ALI.

### STUB1 depletion aggravates stress-induced lung injury and anoxia/reoxygenation (A/R)-elicited damage in cultured lung epithelial cells

**Fig. 3:**
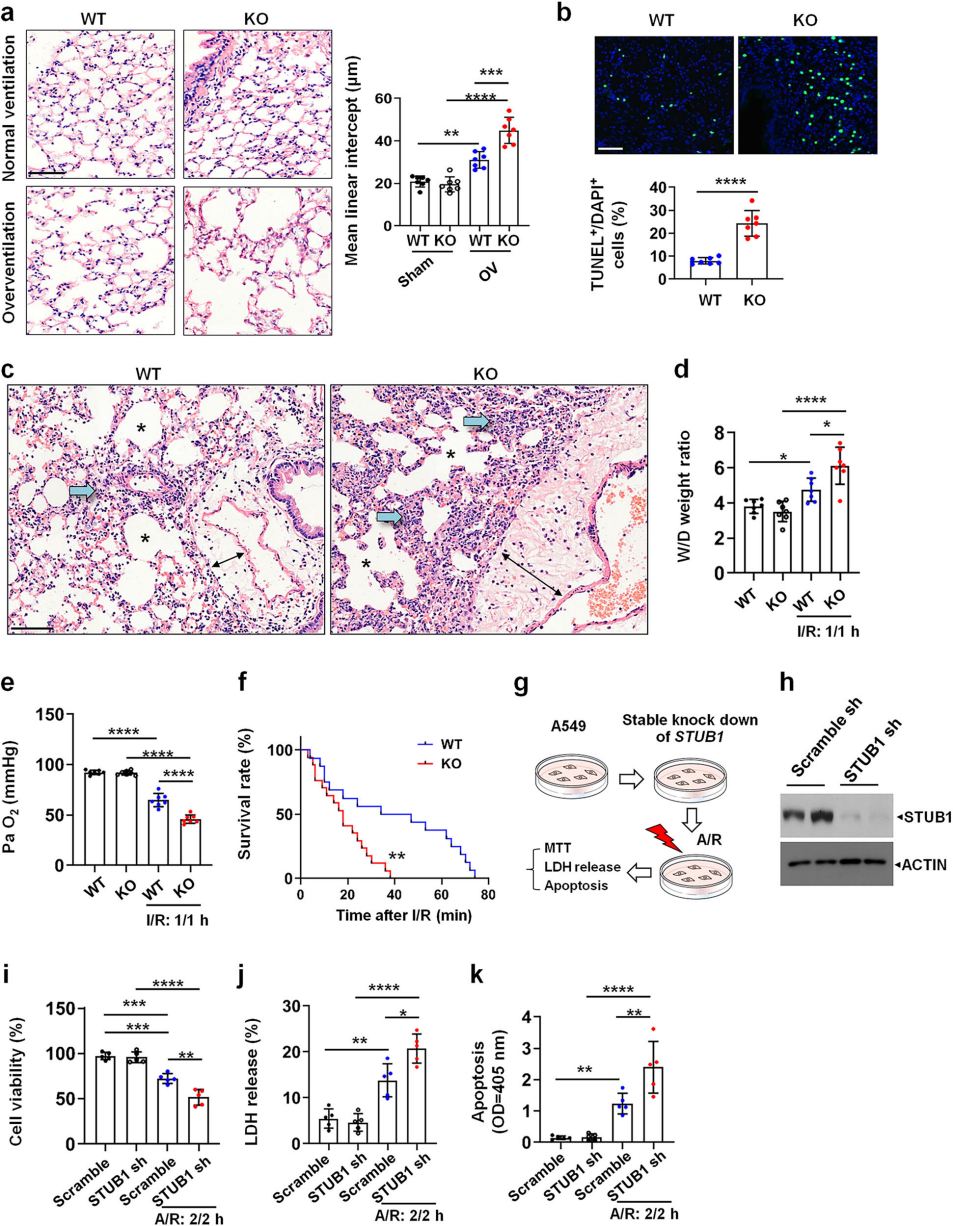
Increased susceptibility of the *Stub1* KO mice to overventilation and I/R-induced lung injury, and in vitro assay with endogenous STUB1 protection against anoxia and reoxygenation (A/R) injury to cultured A549 cells. **a** H&E staining of lung sections derived from WT and KO mice subject to normal ventilation or overventilation (Bar=25 μm). Mean linear intercept measurements (μm) of alveolar spaces reveal a significant difference between the WT and KO lung (*n* = 7/group, **P < 0.01, ***P < 0.001 and ****P < 0.0001, two-way ANOVA followed by Tukey’s post-hoc test). **b** Representative 4% PFA-fixed lung sections stained by TUNEL demonstrated increased apoptosis in KO mice after overventilation treatment (Bar=25 μm). Right panel, quantification of TUNEL–positive cells per field in overventilation-treated WT or KO mice (*n* = 7, ****P < 0.0001, *Student’s t* test). **c** Representative H&E staining of lung sections derived from WT and KO mice subject to ischemia/reperfusion (I/R)-induced left lung injury (Bar=25 μm). Asterisks indicate alveolar spaces. Blue arrows point to inflammatory infiltrates. Double-headed arrows indicate perivascular edema. **d** Lung oedema was measured as the wet/dry weight ratio of the excised lung from WT and KO mice subject to I/R-induced left lung injury (*n* = 7/group, *P < 0.05 and ****P < 0.001, two-way ANOVA followed by Tukey’s post-hoc test). Animals receiving normal low-tidal ventilation without I/R were used as sham controls. **e** Following I/R induction, plasma PaO_2_ concentrations were measur**e**d in deeply anesthetized mice using an oximeter (*n* = 7/group, ****P < 0.0001, two-way ANOVA followed by Tukey’s post-hoc test). **f** Survival rate of WT and KO mice subject to I/R was determined by the log-rank test (*n* = 16, **P < 0.01, Log-rank (Mantel-Cox) test). **g** Schematic representation of the experimental procedure used for the in vitro assay. **h** Stable knockdown of STUB1 in A549 cells was verified using immunoblotting. A549 cells with different transfections were subject to A/R treatment as described in Materials and methods, followed by measurement of cell viability (**i**), lactate dehydrogenase (LDH) cytotoxicity (**j**) and apoptosis (**k**) by spectrophotometry at 570 nm, 490 nm and 405 nm, respectively (*n* = 5/group, *P < 0.05, **P < 0.01, ***P < 0.001 and ***P < 0.001, two-way ANOVA followed by Tukey’s post-hoc test).

To further determine the extent and depth of STUB1’s protective effects, we evaluated the susceptibility of mice to overventilation-induced lung injury by subjecting these animals to ventilation at 30 ml/kg (tidal volume/body weight) for 30 min to induce acute stress to lung resident cells [21]. H&E staining revealed no pathological changes in the lungs derived from either KO or WT mice under normal conditions. In contrast, overventilation resulted in enlarged alveolar space with lung from both KO and WT mice, with a higher mean linear intercept value (a morphological indicator characterizing the enlargement of airspaces in emphysema and the associated severity of structural destruction in lung [22]) being observed in KO lung (Fig. 3a). Meanwhile, 30-min overventilation caused a higher percentage of alveolus resident cells to be labeled with TUNEL (TdT-mediated dUTP nick end labeling) in KO lung when compared to WT lung (Fig. 3b), suggesting enhanced susceptibility to lung injury in the absence of STUB1. In separate experiments, we also tested whether ablation of STUB1 could alter the response of the lung to I/R (ischemia/reperfusion)-induced injury. For this purpose, mice were subjected to 1 h ischemia/1 h reperfusion by clamping of the left pulmonary artery and hilum to introduce complete ischemia and anoxia of the left lung [21]. Compared to WT littermates, KO lung showed more severe perivascular edema (double-headed arrow), alveolar wall swelling (asterisk) and inflammatory infiltrate (blue arrows in Fig. 3c). The W/D weight ratio was significantly higher in the KO group than in the WT controls (Fig. 3d). Pulmonary function revealed by measurement of plasma PaO_2_ concentrations demonstrated that the PaO_2_ in WT mice measured at 64.8 ± 6.6 mmHg, while it dropped to 45.9 ± 4.2 mmHg in KO mice following I/R (*n* = 7 in each, *P* < 0.001, Fig. 3e). Consequently, KO mice displayed a lower survival rate following I/R injury, as compared with WT littermates (Fig. 3f). Next, we tried to figure out the extent that STUB1 may protect lung epithelial cells, by culturing A549^Stub1–/–^ and A549 cells with 5% CO_2_ and 95% N_2_ at 37°C for 2 h followed by reoxygenation for another 2 h to induce A/R injury (Fig. 3g) [21]. Generation of the A549 cells that were stably deprived of endogenous STUB1, using transfection with a mixture of three lentivirus-based shRNAs at a time, was verified by immunoblotting (Fig. 3h). Subsequently, cell viability, lactate dehydrogenase (LDH) release and cell apoptosis were determined to assess epithelial injury. Cell viability was significantly reduced in the A549^Stub1–/–^ cells compared with A549 cells following A/R challenge (Fig. 3i). Likewise, A/R treatment caused augmented LDH release (Fig. 3j) and increased cell apoptosis (Fig. 3k) in A549^Stub1–/–^ cells than in A549 cells. Thus, STUB1 deficiency may exacerbate stress-induced lung epithelial injury both in vitro and in vivo.

### Proper ROS stimulates STUB1 expression via potentiation of Nrf2-mediated transactivation

**Fig. 4:**
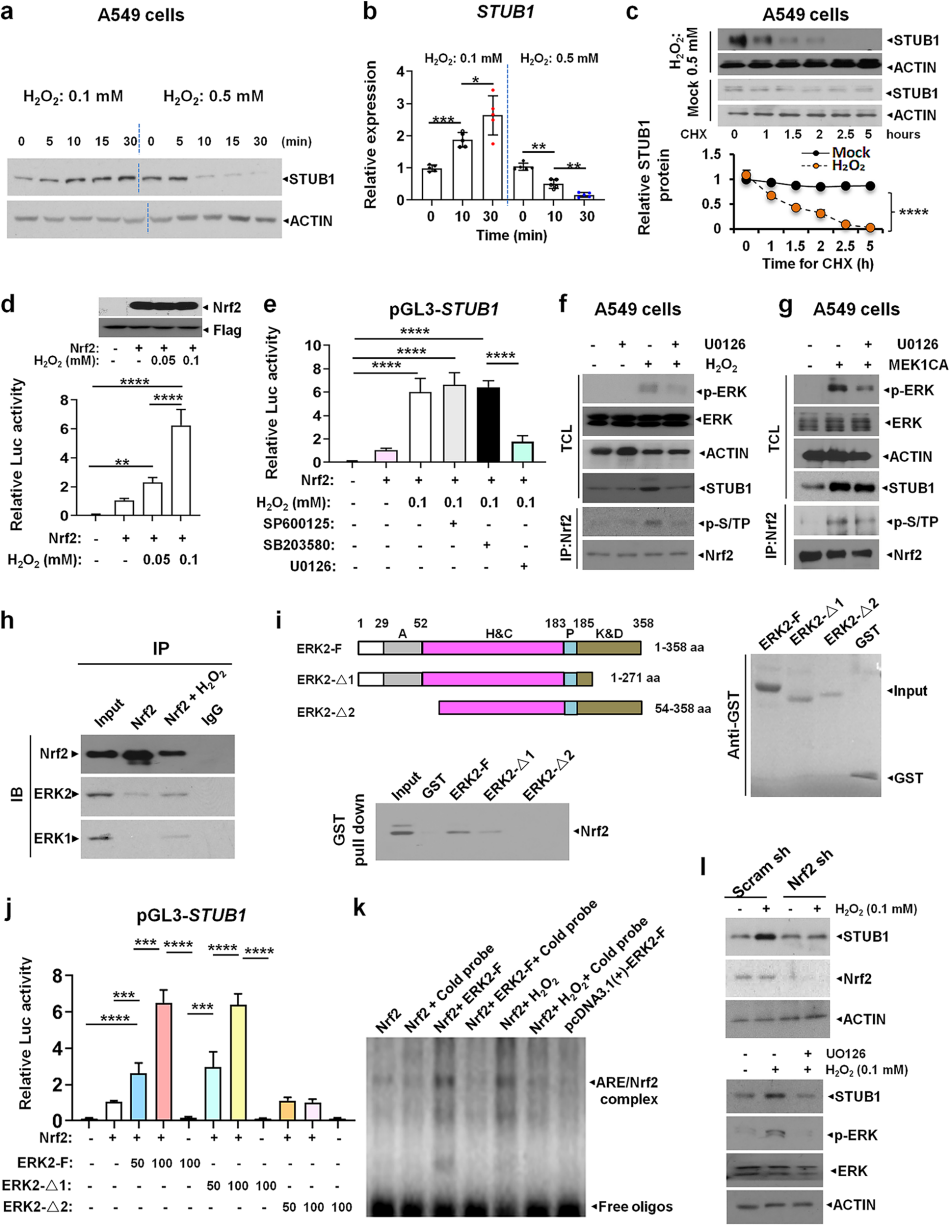
Bidirectional regulation of STUB1 expression by different doses of ROS. **a** A549 cells were treated with different doses of H_2_O_2_ for the indicated durations, followed by immunoblotting analysis. **b** qPCR analyses of *Stub1* mRNA expression following H_2_O_2_ challenge. *n* = 5/group, *P < 0.05, **P < 0.01 and ***P < 0.001 (one-way ANOVA followed by Tukey’s post-hoc test and each bar represents the mean ± SD of results from three experiments using different cell samples). **c** A549 cells were placed in media containing cycloheximide (CHX, 100 μM) to block protein synthesis and then incubated with 0.5 mM H_2_O_2_ to induce oxidative stress, followed by immunoblotting analysis. Lower panel, quantification of the relative expression of STUB1 protein in different settings by Image J. Biological replicates (*n* = 3) for CHX experiments with SEM. Prism 8 Simple linear regression results for slope differences between paired relations: ****P < 0.0001. **d** Nrf2-null 293 T cells were cotransfected with 0.5 μg of Flag-Nrf2 and the indicated reporter plasmid. H_2_O_2_ was applied at the indicated concentrations after 48 h of transfection, and luciferase activity was measured after 6 h of incubation. The error bars indicate the standard deviation (*n* = 4/group, **P < 0.01 and ****P < 0.0001, two-way ANOVA followed by Tukey’s post-hoc test). Absence of altered Nrf2 expression levels by H_2_O_2_ treatment was confirmed by immunoblotting analysis using anti-Nrf_2_ antibody. Flag expression served as a control for transfection efficiency. **e** Nrf2-null 293 T cells were cotransfected with 0.5 μg of Flag-Nrf2 and the indicated reporter plasmid. After 48 h of transfection, the cells were treated with the JNK inhibitor SP600125 (50 μM), P38 inhibitor SB203580 (10 μM), or MEK (ERK upstream signal molecule) inhibitor U0126 (5 mM) for 30 min. Cells were then challenged with 0.1 mM H_2_O_2_ for 6 h and harvested for the measurement of luciferase activity. The error bars indicate the standard deviation (*n* = 4/group, ****P < 0.0001, two-way ANOVA followed by Tukey’s post-hoc test). **f** A549 cells were incubated with U0126 (5 mM) for 30 min, prior to challenge with 0.1 mM H_2_O_2_ for another 6 h. Cells were then lysed in denaturing conditions. Cell lysates were diluted and immunoprecipitated with anti-Nrf2 antibodies. Immunoblotting was performed using either precipitated samples or total cell lysates (TCL) with the indicated antibodies. p-S/TP, antibodies specific for phosphorylated serine or threonine residues adjacent to a proline. **g** A549 cells were transiently transfected with a lentiviral pHAGE-MEK1CA expressing a constitutive active form of the kinase MEK1 (MEK1CA) acting upstream of ERK1/2, using Lipofectamine 3000. 48 h later, cells were incubated with U0126 (5 mM) for 30 min, prior to cell harvest. Cells were subsequently lysed in denaturing conditions. Cell lysates were diluted and immunoprecipitated with anti-Nrf2 antibodies. Immunoblotting was performed using either precipitated samples or TCL with the indicated antibodies. **h** Nrf2 associates with ERK in vivo. Co-immunoprecipitations were performed with anti-Nrf2 antibody using A549 cells that were transfected with Flag-Nrf2, followed by treatment with 0.1 mM H_2_O_2_ for 6 h, and immunoblotting analyses were done with anti-Nrf2 or anti-ERK1/_2_ antibodies. **i** Nrf2 directly interacts with the N-terminal domain of ERK2 in vitro. Bacter**i**ally expressed GST-ERK2 mutants were mixed with recombinant Flag-Nrf2 proteins, and GST pull-down was performed as described. Washed beads were subjected to SDS-PAGE and immunoblotted with anti-Nrf2 antibody. Upper panel, a scheme showing deletion mutants of ERK-2. A, ATP binding site; H&C, hinge region and catalytic HRD motif; K&D, kinase insert domain and common docking domain; P, phosphorylation site. **j** The N-terminal but not the C-terminal region of ERK2 is required for the full suppression of Nrf2 transactivation. 293 T cells were cotransfected with 0.5 μg of Flag-Nrf2 and the indicated reporter plasmid, along with 50 or 100 ng of ERK2 deletion mutant expression plasmids. After 48 h of transfection, luciferase activity was measured. The error bars indicate the standard deviation (*n* = 4/group, ***P < 0.001 and ****P < 0.0001, two-way ANOVA followed by Tukey’s post-hoc test). **k** ERK2 enhances the DNA binding activity of Nrf2. A549 cells were cotransfected with Flag-NRF2 and pcDNA3.1( + )-ERK2-F as described. 48 h later, cells were treated with 0.1 mM H_2_O_2_ for another 6 h, followed by isolation of nuclear protein extracts and subsequent electrophoretic mobility shift assay (EMSA), using 1 μg of Nrf2-enriched nuclear extracts and 30 mM of custom DIG-labeled probes corresponding to the putative Nrf2-binding ARE motifs. A competition assay was performed with a 50-fold excess of cold ARE oligomer. Positions of the specific protein-DNA complex and free probe were indicated. **l** Upper panel, A549 cells were transiently transfected with Nrf2 shRNA or Scramble shRNA using Lipofectamine 3000. 48 h later, cells were harvested and treated with 0.1 mM H_2_O_2_ for 6 h, followed by immunoblotting. Lower panel, A549 cells were incubated with U0126 (5 mM) for 30 min, prior to challenge with 0.1 mM H_2_O_2_ for another 6 h. Cells were then lysed and subjected to immunoblotting.

Oxidative stress, resulting from overproduction of ROS, is a leading etiology that causes lung damage and consequently leads to various disease states (e.g., LPS- or I/R-induced lung injury [23]). To study a potential involvement of ROS in the direct regulation of STUB1 expression, we treated A549 cells with low dose (0.1 mM) or high dose (0.5 mM) of H_2_O_2_. The selection of these two dosages was made based on the following criteria: (1) The PubMed database indicates that concentrations of H_2_O_2_ within the range of 0.1-0.5 mM have been utilized to induce oxidative damage in cellular models in half of the studies [24]. (2) Both low (0.1 mM) and high (0.5 mM) doses of H_2_O_2_ have been commonly employed to provoke oxidative damage in cellular models derived from AT2s [25, 26]. (3) Most significantly, the maximum dosage of H_2_O_2_ that can activate the transactivating function of Nrf2 and promote the up-regulation of ARE-mediated gene expression by Nrf2 is 0.5 mM. Conversely, exposure to high doses of H_2_O_2_ (e.g., 1 mM) results in the nuclear exclusion of Nrf2, a reduction in transcriptional regulatory activity, and a down-regulation of ARE-mediated gene expression [27]. Interestingly, low dose (0.1 mM) of H_2_O_2_ steadily stimulated STUB1 expression in a time-dependent manner, while high dose (0.5 mM) of H_2_O_2_ displayed a clear-cut inhibitory effect (Fig. 4a). Similar effects were also demonstrated by qPCR at the mRNA level (Fig. 4b). We subsequently treated A549 cells with 100 μM cycloheximide (CHX) to block protein synthesis and then challenged cells with 0.5 mM H_2_O_2_. Under these stress conditions, STUB1 protein exhibited a protein half-life less than 2 h (Fig. 4c). A parallel experiment confirmed that 0.5 mM H_2_O_2_ had no effects on cell viability (Supplementary Fig. S6). Thus, high levels of H_2_O_2_ may decrease STUB1 expression by directly impairing its protein half-life. Because nuclear factor erythroid 2-related factor 2 (Nrf2) is a major transcription factor that modulates ROS-related lung damage [28], and because our in silico screen has identified a potential antioxidant response element (ARE) for Nrf2 binding in the promoter region of *STUB1* (Supplementary Fig. S7), we hypothesized that ROS may promote the transactivation of Nrf2 on the promoter of the *STUB1* gene. Nrf2-null 293 T cells were cotransfected with Flag-Nrf2, along with pGL3-STUB1 and pRL-TK Renilla reporter plasmids, and treated with or without H_2_O_2_. Ectopic expression of Nrf2 enhanced the promoter activity of *STUB1*, and co-treatment with H_2_O_2_ further potentiated Nrf2 transactivation dose-dependently (lower panel, Fig. 4d). Contrarily, mutations at the sequence of the Nrf2 motif or co-treatment with the ROS scavenger N-Acetyl-L-cysteine (NAC) could both completely abrogate the H_2_O_2_-elicited *STUB1* promoter activity (Supplementary Fig. S8). Of note, the overexpression of the exogenous Nrf2 may circumvent its regulation by Keap-1, and it is very likely that the levels of overexpression of Nrf2 were far in excess of endogenous Nrf2 levels in this system. To exclude this possibility, we transfected Nrf2-null 293 T cells with full-length pBABE-Nrf2 or with mutant Nrf2 (namely C506S, L4A), containing the C506S mutation and four leucine zipper (ZIP) mutations (L553A_L557A_L560A_L562A), and then challenged the cells with 0.1 mM H_2_O_2_. In accordance with the previous reports that the mutant Nrf2, containing C506S mutation and four leucine zipper (ZIP) mutations, is significantly less efficient in activating ARE-mediated gene expression [29, 30], we observed that the full-length Nrf2 markedly potentiated H_2_O_2_-induced transactivation of the *STUB1* whereas the mutant Nrf2 failed to do so (Supplementary Fig. S8). Additionally, Nrf2 protein levels were unaffected upon H_2_O_2_ challenge (upper panel, Fig. 4d). Thus, ROS signaling may stimulate STUB1 expression, at least in part, by promoting the Nrf2-mediated transactivation of the *STUB1* gene. During ALI, cellular response to oxidative stress often involves mitogen-activated protein kinases (MAPKs) that act in concert to influence cell fate [31]. We therefore checked which member of MAPKs was involved in the ROS-mediated potentiation of Nrf2 transactivation. Nrf2-null 293 T cells were cotransfected with Flag-Nrf2, along with pGL3-STUB1 and pRL-TK Renilla reporter plasmid, and treated with different inhibitors for 30 min prior to H_2_O_2_ challenge. The H_2_O_2_-mediated promotion of Nrf2 transactivation was effectively abrogated by cotreatment with U0126 for the *STUB1* promoter, but was unaffected by the treatment with SP600125 or SB203580 (Fig. 4e), indicative of the involvement of the ERK signaling pathway in the modulation of ROS-mediated stimulation of Nrf2 transactivation. To verify that Nrf2 is a bona fide substrate of ERK in vivo, A549 cells were incubated with U0126 for 30 min, followed by treatment with 0.1 mM H_2_O_2_ for another 30 min. Cell lysates were then immunoprecipitated with anti-Nrf2 antibody, and subsequent immunoblotting using antibodies that recognize phosphorylated serine or threonine residue adjacent to a proline (p-S/TP), the consensus motif phosphorylated by ERK [32], demonstrated that phosphorylation of Nrf2 was augmented by H_2_O_2_ but totally abolished by co-treatment with the ERK inhibitor U0126 (Fig. 4f and Supplementary Fig. S9). In the same line, transfection with pHAGE-MEK1CA expressing a constitutive active form of the kinase MEK1 (MEK1CA) acting upstream of ERK1/2 could consistently enhance phosphorylation of Nrf2, regardless of cotreatment with U0126 or H_2_O_2_ (Fig. 4g and Supplementary Fig. S9). MEK1 is typically activated by phosphorylation on serine 218 and 222 [33], and recent studies show that introduction of mutations at these two sites produces a constitutively active MEK1 form (MEK1CA) that is also resistant to MEK inhibitors, including U0126 [34]. Considering that the rates of ERK2 autophosphorylation can be increased by several synergistic mutations such as Lys73Pro and Ser151Asp [35], the aforementioned consistent activation of ERK2 and resistance to U0126 by MEK1CA may stem from an enhanced ERK2 autophosphorylation. To ask whether Nrf2 protein interacts with ERK in vivo, a Co-IP assay was performed using Flag-Nrf2- transfected A549 cells treated with H_2_O_2_. When Nrf2 was precipitated with an anti-Nrf2 antibody, ERK2 but not ERK1 coprecipitated with Nrf2. The co-precipitation between Nrf2 and ERK1/2 was apparently strengthened by cotreatment with H_2_O_2_ (Fig. 4h and Supplementary Fig. S9). To further validate the direct functional interaction between ERK2 and Nrf2 in vitro, we performed a GST pull-down assay using GST fusion proteins of full-length ERK2 or its deletion mutants. Nrf2 interacted with full-length ERK2 and the ERK2 N-terminal region, but not with the C-terminal region (Fig. 4i and Supplementary Fig. S9). We then tested whether the ERK2 N-terminal region alone was sufficient for the stimulation of Nrf2 transactivation. Transient transfection of full-length ERK2 and ERK2 N-terminal region, but not the C-terminal region, was able to promote Nrf2 transactivation (Fig. 4j). To further determine whether the ERK2 mutants are catalytically active in vitro, we performed an in vitro kinase assay to test the ERK2 mutants for their capability to phosphorylate myelin basic protein (MBP, a frequently used substrate for ERK) [36]. As expected, ERK2-F, as well as the mutants, exhibited no activity in the absence of MEK1. To further elucidate whether the ERK2 mutants could be activated by MEK1-mediated dual phosphorylation, we incubated all ERK2 proteins with an activated MEK1 and ATP. Subsequent in vitro kinase assay showed that both ERK2-F and ERK2-△1 could be further activated by MEK1-mediated phosphorylation, whereas ERK2-△2 failed to do so. Thus, the activity of the purified recombinant ERK2-△1 proteins is intrinsic and depends on upstream activation (Supplementary Fig. S10). Because the DNA-binding domain of Nrf2 might be involved in interaction with ERK2, we tested whether ERK2 could augment the DNA-binding activity of Nrf2 onto the *STUB1* chromatin using the EMSA assay. Nrf2 in the nuclear extracts from A549/Flag-Nrf2 cells clearly formed a shifted DIG-labeled band, which was noticeably enhanced by cotreatment with H_2_O_2_ or cotransfection with pcDNA3.1( + )-ERK2-F. Instead, ERK2-F alone failed to bind the *STUB1* chromatin. Additionally, competition with a 50-fold excess of cold probe totally abrogated the shifted DIG-labeled band, regardless of cotreatment with H_2_O_2_ or cotransfection with pcDNA3.1( + )-ERK2-F, confirming the assay specificity (Fig. 4k and Supplementary Fig. S11). The available data support a close link among Nrf2, ERK2 and STUB1. To validate this from a functional standpoint, we next investigated the effects of UO126 co-treatment or Nrf2 knockdown on the H_2_O_2_-induced changes in endogenous STUB1 expression. As expected, co-treatment with UO126 or knockdown of Nrf2 both completely abolished H_2_O_2_-induced STUB1 expression in A549 cells (Fig. 4l and Supplementary Fig. S9). Altogether, these findings implied that ERK2 may strengthen the DNA-binding activity of Nrf2, thus facilitating the transactivation of *Stub1*.

### STUB1 induces atypical polyubiquitination of salt-inducible kinase 3 (SIK3)

**Fig. 5:**
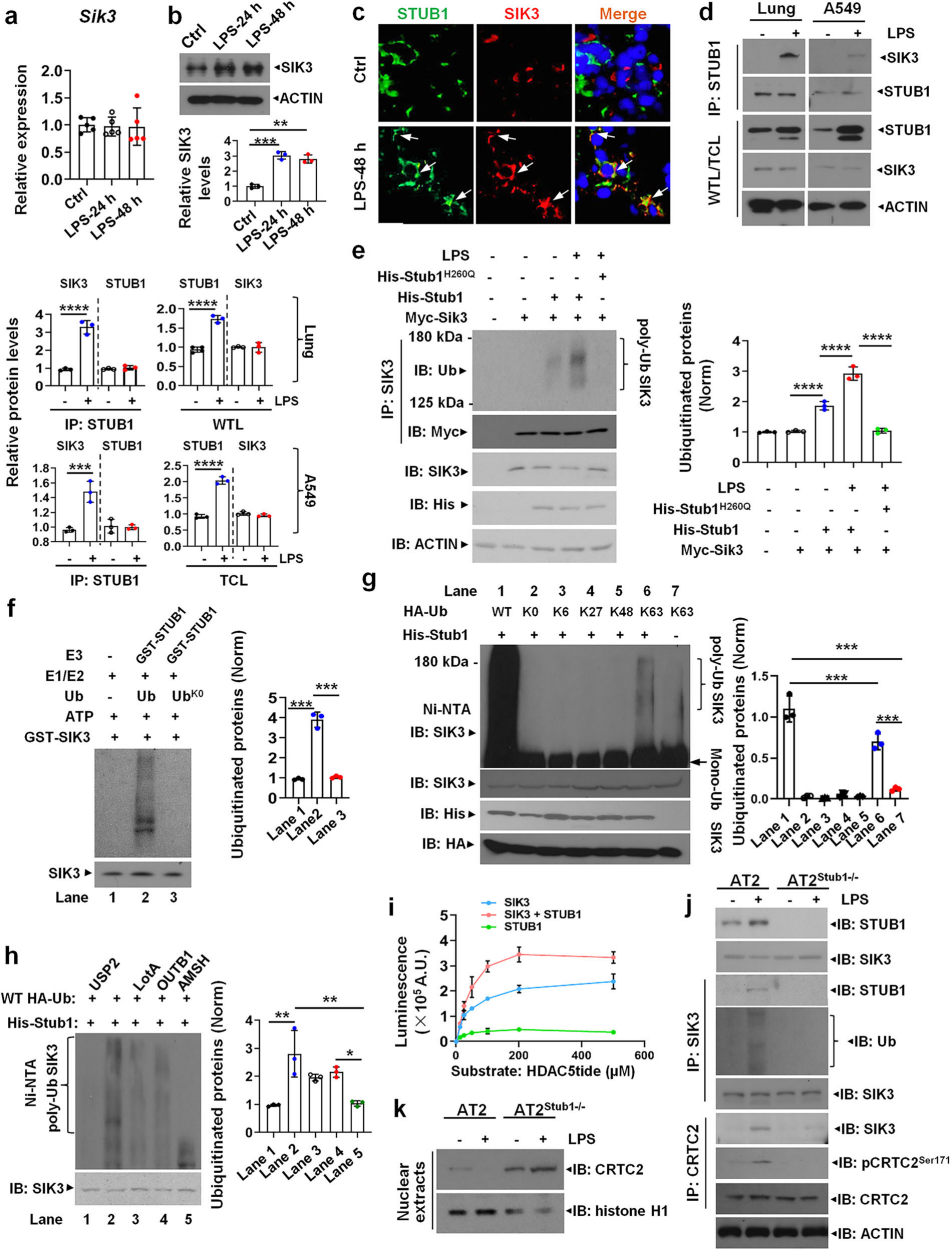
STUB1-induced atypical polyubiquitination mediates kinase activity and binding capacity of salt-inducible kinase 3 (SIK3). **a** Relative expression levels of *Sik3* mRNA in the lung were evaluated by qPCR at 24 h or 48 h after intratracheal administration of PBS or LPS, *n* = 5/group (one-way ANOVA followed by Tukey’s post-hoc test and each bar represents the mean ± SD of results from three experiments using different tissue samples). **b** Immunoblotting analysis of SIK3 along the pathogenesis of ALI. β-ACTIN served as a loading control. Densitometric scanning of immunoblots was performed in which the level of a target protein was normalized against the protein level in Ctrl group, which was arbitrarily set at 1. Lower panel, quantification of the relative expression of SIK3 protein in different settings by Image J (*n* = 3, **P < 0.01 and ***P < 0.001, two-way ANOVA followed by Tukey’s post-hoc test). **c** Double immunofluores**c**ence labeling suggests STUB1 was colocalized with SIK3 in LPS-treated lung (arrows), but not in Ctrl lung. Bar = 25 μm. **d** It is important to note that following treatment with LPS, we consistently observed an upregulation of STUB1 expression, regardless of whether lung tissue or A549 cells were utilized. To confirm that the heightened association between STUB1 and SIK3 was not a result of the increased STUB1 expression, we made significant efforts to standardize the IP: STUB1 levels in the Co-IP assay. This explains why the STUB1 protein levels in the WTL (whole tissue lysates)/TCL did not correspond with those observed in the IP assay. Upper panel, total protein was prepared either from lung tissues from LPS-treated mice at 24 h post-treatment or from LPS (100 ng/ml)-challenged A549 cells at 12 h after LPS incubation. Protein samples were then diluted and immunoprecipitated with anti-STUB1 antibodies. Immunoblotting was performed using either precipitated samples or TCL with the indicated antibodies. Lower panel, quantification of the relative protein expression in different settings by Image J (*n* = 3, ***P < 0.001 and ****P < 0.0001, two-way ANOVA followed by Tukey’s post-hoc test). **e** 293 T cells were transfected with pcDNA3.1-His-Stub1 and pCMV6-Myc-Sik3, in the presence or absence of co-transfection with an inactive mutant His-Stub1^H260Q^. 48 h after transfection, cells were treated with LPS (100 ng/ml) for another 12 h. Cells were then lysed and subjected to immunoprecipitation using anti-SIK3 under denaturing conditions, followed by immunoblotting with the indicated antibodies. Right panel, quantification of the relative ubiquitinated proteins in different settings by Image J (*n* = 3, **P < 0.01 and ****P < 0.0001, two-way ANOVA followed by Tukey’s post-hoc test). **f** In vitro polyubiquitination assays were carried out as described in Materials and Methods, using wild-type ubiquitin or a lysine-lacking variant (Ub^K0^). Immunoblot of anti-SIK3 to detect SIK3 and SIK3 conjugates. Right panel, quantification of the relative ubiquitinated proteins in different settings by Image J (*n* = 3, ***P < 0.001, two-way ANOVA followed by Tukey’s post-hoc test). **g** 293 T cells were transfected with pRK5-HA-Ubiquitin-WT ubiquitin, a lysine null mutant (pRK5-HA-Ubiquitin^K0^), or a single-lysine-containing mutant as indicated, along with His-Stub1 and Myc-Sik3. At 48 h posttransfection, cells were lysed, and lysates were incubated with Ni-NTA beads rotating at 4°C overnight. Immunoblotting was performed using the indicated antibodies. Right panel, quantification of the relative ubiquitinated proteins in different settings by Image J (*n* = 3, **P < 0.01 and ***P < 0.001, two-way ANOVA followed by Tukey’s post-hoc test). **h** The immunoprecipitates in **5** **g** were digested with USP2, a broad-spectrum DUB, or with K48 (OUTB1), K6 (LotA) or K63 (AMSH) chain-type-specific DUBs at 37°C for 30 min, followed by immunoblotting analysis of SIK3 expression. Right panel, quantification of the relative ubiquitinated proteins in different settings by Image J (*n* = 3, *P < 0.05 and **P < 0.01, two-way ANOVA followed by Tukey’s post-hoc test). **i** 293 T cells were cotransfected with His-Stub1 and Myc-Sik3. On the 4^th^ day after transformation, cells were harvested and treated with LPS (100 ng/ml) for another 12 h. The Myc fusion protein was then immunoprecipitated using anti-Myc beads, followed by measurement of in vitro kinase activity of Myc-SIK3 proteins using HDAC5tide as a substrate. **j** AT2s and AT2^Stub–/–^ cells were treated with LPS (100 ng/ml) for 12 h. Cells were then harvested, and cell lysates were immunoprecipitated with anti-SIK3 or anti-CRTC2 antibodies, and the precipitates were then probed with different antibodies as indicated. **k** Upon treatment with LPS, AT2s and AT2^Stub–/–^ cells were fractionated into nuclear fractions, followed by immunoblotting analysis of CRTC2. Blots were simultaneously probed with the antibody against the nuclear marker histone H1 to verify the fidelity of the fractionation.

As further exploration of the molecular basis underpinning STUB1 action, we employed UbiBrowser 2.0 (http://ubibrowser.bio-it.cn/ubibrowser_v3/) to predict proteome-wide E3 ligase-substrate interactions, and have identified SIK3 as one of the top substrates shown to interact with STUB1 (Supplementary Table 1). qPCR analysis using lung tissues showed that *Sik3* mRNA was unaffected following LPS challenge (Fig. 5a), whereas SIK3 protein levels were steadily increased in lung samples prepared from LPS-treated mice (Fig. 5b). In contrast, the expression levels of SIK1 and SIK2, whether at the mRNA or protein levels, were similar in lung samples obtained from naive or LPS-treated WT/KO mice, thereby ruling out the possibility of any potential functional compensation among the members of the SIK kinase family (Supplementary Fig. S12). These findings implicated that pulmonary SIK3 may demand precise posttranslational control of its function during ALI. Consistently, double immunofluorescence using lung tissues revealed that STUB1 was only colocalized with SIK3 in LPS-treated mice but not under resting conditions (Fig. 5c). In accordance with these descriptive data, Co-IP demonstrated that LPS challenge significantly stimulated STUB1 protein expression and enhanced association between STUB1 and SIK3 in lung tissues and A549 cells, but had no effects on expression levels of SIK3 protein (Fig. 5d). To test whether SIK3 was a substrate of STUB1, Myc-Sik3 and His-tagged wild-type Stub1 or the mutant Stub1 deficient in enzymatic activity (namely the E3 ligase dead mutant H260Q [14]) were coexpressed in 293 T cells, followed by LPS challenge. Accumulation of polyubiquitinated SIK3 was detected in cells expressing wild-type Stub1, but not in cells expressing inactive mutant H260Q. Furthermore, LPS treatment markedly augmented STUB1-mediated polyubiquitination of SIK3 (Fig. 5e). Additionally, transfection with His-Stub1 but not Myc-Sik3 in 293 T cells completely abolished LPS-enhanced polyubiquitination of SIK3, excluding again the possibility of a nonspecific protein coming down in the IP assay (Supplementary Fig. S13). Thus, STUB1 could induce SIK3 polyubiquitination in an E3 ligase-dependent manner. Likewise, using in vitro polyubiquitination assays with wild-type (Ub) or a ubiquitin mutant without lysines (Ub^K0^), we found that the higher molecular-weight polyubiquitinated SIK3 was only detected in the presence of Ub (Fig. 5f). Therefore, STUB1 may mediate polyubiquitylation of the SIK3 rather than multiple monoubiquitylation. We were then curious which lysine residue(s) in the ubiquitin molecules may be responsible for the assembly of the atypical polyubiquitin chains on SIK3 induced by STUB1. SIK3 and STUB1 were co-expressed with HA-ubiquitin or lysine-lacking variant (HA-Ubiquitin^K0^) or various mutants with a single lysine in 293 T cells. Apparently, the addition of STUB1 could remarkably enhance polyubiquitination of SIK3 in the presence of wild-type Ub or Ub-K63 (Fig. 5g). Of note, transfected SIK3 was more heavily polyubiquitinated in the presence of wild-type Ub than of Ub-K63, possibly due to the activities of some endogenous E2 or E3 ligases. To elucidate the ubiquitin chain topology of the STUB1 substrate, we incubated immunoprecipitates (IPs) from Fig. 5g with the nonselective USP2 or with K48- (OUTB1), K6- (LotA), or K63- (AMSH) specific deubiquitinating enzymes (DUBs), followed by immunoblotting using antibodies against SIK3. Notably, both USP2 and AMSH were able to effectively eliminate the total polyubiquitination signal (Fig. 5h). Consequently, it appears that STUB1 preferentially assembles polyubiquitination chains at K63. It is important to mention that while the introduction of a K63 mutation or cotreatment with AMSH both effectively abolished STUB1-mediated polyubiquitination of SIK3, a slight polyubiquitination was often observed at the lower molecular weight (Fig. 5g, h). These results, which indicate a low stoichiometry of polyubiquitination, suggest that although K63 seems to be the primary linkage associated with the polyubiquitination of SIK3, the possibility of other linkages (such as hybrid ubiquitin linkages or M1 chains) at low levels cannot be disregarded. To explore whether STUB1 may exert effects on the kinase activity of SIK3, we performed in vivo transformation plus in vitro kinase assay of SIK3 using HDAC5tide as a substrate. 293 T cells were cotransfected with His-Stub1 and Myc-Sik3. On the 4th day after transformation, cells were harvested and treated with LPS (100 ng/ml) for another 12 h. The Myc fusion protein was then immunoprecipitated using anti-Myc beads, followed by measurement of in vitro kinase activity of Myc-SIK3 proteins using HDAC5tide as a substrate. Co-transfection with His-STUB1 markedly increased HDAC5tide phosphorylation by SIK3 in 293 T cells (Fig. 5i). Disrupted CRTC2 (CREB-regulated transcription co-activator 2)-CREB signaling downstream of SIKs serves as the major mechanistic pathway mediating tissue inflammation [37]. CRTC2 is usually phosphorylated at Ser171 and becomes sequestered in the cytoplasm, and dephosphorylated CRTC2 enters the nucleus to promote CREB-dependent transcription at inflammatory gene loci, thus leading to cytokine expression (e.g. IL-2, IL-6 and TNF-α) [38]. In this context, we next determined how STUB1 deficiency affects the binding of SIK3 to its downstream substrate CRTC2 in LPS-challenged primary AT2s. Intact expression of endogenous STUB1 significantly enhanced the binding of SIK3 to CRTC2, as well as phosphorylation of CRTC2 at Ser171, in LPS-treated AT2s, but failed to do so in AT2^STUB1–/–^ cells (Fig. 5j). In line with these biochemical changes, CRTC2 expression was observed to be accumulated more in the nuclei of LPS-challenged AT2^STUB1–/–^ cells, when compared to that in LPS-challenged AT2s (Fig. 5k). Consequently, STUB1 deletion potentiated LPS-stimulated CREB-mediated cytokine expression in AT2^STUB1–/–^ cells (Supplementary Fig. S14). These findings thus establish SIK3/CRTC2 signaling as a core mechanism, probably in concert with CREB, for the regulation of inflammatory responses downstream of STUB1 in LPS-stimulated AT2s. We therefore proposed that the signaling cascade in part mediated by STUB1/SIK3/substrates might be required for the proper response to stress-induced alveolar injury.

### Intranasal delivery of constitutive-active SIK3 mutant protects against LPS-induced lung injury in KO mice

**Fig. 6:**
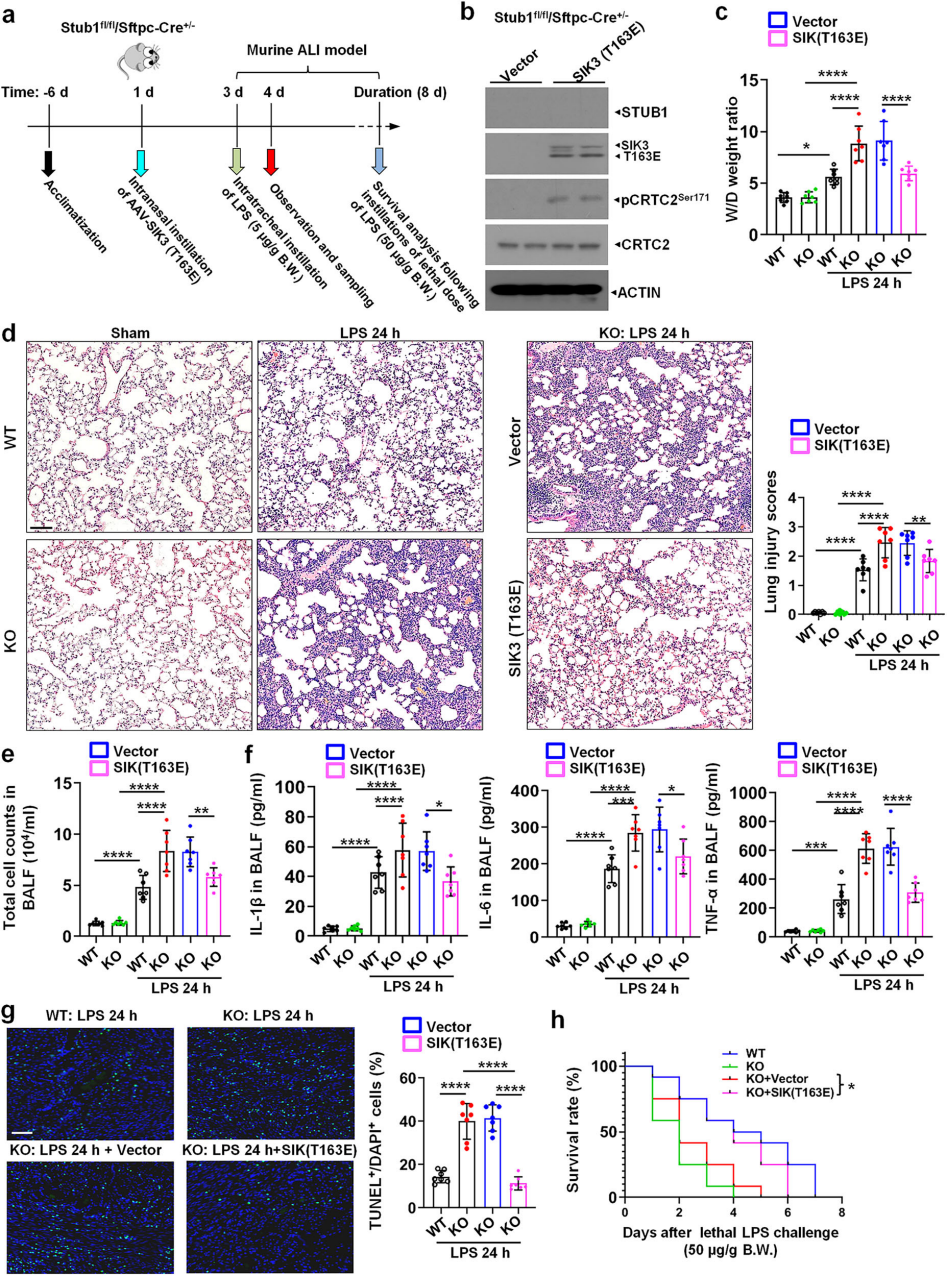
Overexpression of a constitutive-active SIK3 mutant ameliorates LPS-induced lung injury and mortality in KO mice. **a** Experimental design for rescue-assay using an intranasal delivery of adeno-associated virus (AAV)-packaged constitutive-active SIK3 mutant (T163E) in Stub1-deficient mice. **b** At 4 d after intranasal instillation of AAV-SIK3 (T163E), lungs were collected from vector- or AAV-SIK3-treated KO mice, and immunoblotting analyses were performed using the indicated antibodies. **c** Lung oedema was measured as the wet/dry weight ratio of the excised lung from different model mice 24 h after intratracheal administration of PBS or LPS, *n* = 7/group, *P < 0.05, ****P < 0.0001, two-way ANOVA followed by Tukey’s post-hoc test. **d** H&E staining of lung sections derived from sham control or LPS-challenged different model mice at 24 h after intratracheal administration of LPS. Bar=25 μm. Right panel, morphometric analyses of lung by lung injury scores on a 0-4-point scale in all groups, *n* = 7/group, **P < 0.01, ****P < 0.0001, two-way ANOVA followed by Tukey’s post-hoc test. **e** Total cell counts in BALF from sham control or LPS-challenged different model mice at 24 h after intratracheal administration of LPS, *n* = 7/group, **P < 0.01, ****P < 0.0001 (two-way ANOVA followed by Tukey’s post-hoc test). **f** Levels of IL-1β, IL-6, and TNF-α in BALF from different model mice 24 h after intratracheal administration of PBS or LPS (5 μg/g) as determined by ELISA, *n* = 7/group, *P < 0.05, ***P < 0.001 and ****P < 0.0001 (two-way ANOVA followed by Tukey’s post-hoc test). **g** Representative 4% PFA-fixed lung sections stained by TUNEL demonstrated reduced apoptosis in KO mice after intranasal delivery of AAV-SIK3 (T163E) (Bar=25 μm). Right panel, quantification of TUNEL-positive cells per field in sham control or LPS-challenged different model mice (*n* = 7, ****P < 0.0001, two-way ANOVA followed by Tukey’s post-hoc test). **h** Survival rate following instillations of ALI-inducing lethal LPS (50 μg/g B.W.) in WT, as well as vector- or AAV-SIK3-treated KO mice, was assessed by Log-rank (Mantel-Cox) test (*n* = 12/group,*P < 0.05).

The preceding data indicate that alveolar STUB1 may exert its protective role through a direct regulation of the SIK3 signaling upon LPS challenge. To address this from a functional standpoint, we performed the rescuing experiments in vivo, using intranasal instillation of AAV-SIK3 (T163E), a constitutive-active SIK3 mutant [39], in LPS-induced murine ALI model (Fig. 6a). Intranasal administration of AAV-packaged SIK3 (T163E) markedly activated the SIK3 signaling in STUB1-deficient lung tissues (evidenced by increased phosphorylation of its downstream substrate CRTC2 at Ser171), even under resting conditions (Fig. 6b). As a result, constitutive expression of active SIK3 effectively but partially reversed STUB1 depletion-induced W/D weight ratio in LPS-treated KO mice (Fig. 6c). Meanwhile, SIK3 (T163E) overexpression significantly ameliorated lung injury-related histological changes (such as thickening of alveolar walls, inflammatory infiltrates and disruption in alveolar structure) in KO mice 24 h following LPS challenge (Fig. 6d). In keeping with this, constitutive activation of SIK3 noticeably ameliorated STUB1 deficiency-elicited total cell counts in BALF (Fig. 6e), secretion of inflammatory cytokines into BALF (Fig. 6f), as well as alveolar epithelial apoptosis (Fig. 6g), at 24 h after LPS treatment. Importantly, administration of AAV-packaged SIK3 (T163E) in vivo markedly improved survival rate of KO mice after lethal LPS challenge (Fig. 6h). Furthermore, the administration of AAV over a duration of 30 days did not impact body weight, fundamental blood chemistry, or the histological examination of various organs, including the liver, lungs, and kidneys, thereby affirming the long-term safety of our AAV assay (Supplementary Fig. S15). Collectively, the available data provided unequivocal evidence that SIK3 may serve as a major downstream effector of STUB1 during ALI.

## Discussion

ARDS represents a swiftly advancing type of respiratory failure in patients with ALI, contributing to approximately 10% of intensive care unit (ICU) admissions and linked to consistently elevated mortality rates between 35% and 46% in severe instances [40, 41]. Even among patients whose ARDS improved quickly, the mortality rate was 31% [42]. Present therapeutic approaches are insufficiently supportive, largely because of a limited understanding of their molecular underpinnings. Consequently, it is essential to develop novel anti-inflammatory medications, identify biomarkers for early diagnosis and prognosis, and tailor treatment strategies accordingly. Despite the indispensability of STUB1 signaling in integrating proteasomal degradation and cellular balance, which has remained unquestionable in recent years, numerous studies have been focusing on perturbations in this signaling that contribute to dysregulation of immunogenicity and tumorigenicity [14]. Considerably less is known about the role and regulation of STUB1 in non-immune system responses to stress. We show here that in the lung, (i) STUB1 was specifically expressed in AT2s (Fig. 1h–j). (ii) Exposing mice to intratracheal LPS progressively induced STUB1 expression (Fig. 1f, g). (iii) Deletion of AT2 *Stub1* remarkably aggravated LPS-induced lung injury and led to increased mortality (Fig. 2). (iv) STUB1 deficiency potentiates lung injury in different animal model systems (Fig. 3). Thus, viability of the AT2 STUB1 determines severity and outcomes of stress-induced lung injury.

Our findings descriptively link specific expression of STUB1 and AT2s (Fig. 1h, i). This observation is novel and important. Compared to the monotonous function of AT1 in allowing gas exchange, AT2 provides wider functionality, including producing surfactant, mediating host defense and acting as local progenitors for AT1, thus leaving it as a primary target for inhaled substances [43]. In this regard, injury to AT2s is generally believed to be a major initiating event for many lung diseases, including ARDS, sepsis, lung inflammation, and ventilator-induced lung injury [44]. An emerging body of studies has identified STUB1 as a dual-function protein with a role in polyubiquitination as a co-chaperone with heat shock proteins, and as an E3 ligase [45]. This functional variability may affect its subcellular expression in a very cell–type–specific manner [46]. Nevertheless, the exclusive expression of STUB1 in AT2s indeed predicted a potential regulatory role of STUB1 in endogenous repair and recovery following injury. In support, specific knockout of *Stub1* in AT2s, by means of AT2-specific driver (*Sftpc*) of Cre recombinase, significantly exacerbated lung injury-related changes, including air space enlargement, AT2 apoptosis, accumulation of alveolar edema, inflammatory infiltrates and disruption in alveolar structure, in three different rodent models of emphysema involving overventilation, tracheal application of LPS and lung I/R injury (Figs. 2, 3). Our results contradict a prior study conducted by Wei Q *et al*., which indicated that IL-4Rα plays a role in mediating the protective effects of STUB1 against airway inflammation in STUB1^–/–^ mice [47]. There are two factors that may clarify this discrepancy: (1) Wei Q et al. utilized different mouse models, specifically asthma and chronic obstructive pulmonary disease (COPD), which differ from the ALI model used in our research. ALI/ARDS, along with asthma and COPD, represent two major respiratory conditions that can significantly affect individuals. Although ALI/ARDS, COPD and asthma may share some symptoms, such as difficulty breathing and low oxygen levels, they have distinct causes and characteristics. ALI is characterized by an acute onset, whereas asthma and COPD are chronic conditions. ALI arises from either direct or indirect lung injury, while asthma and COPD are generally triggered by allergens or irritants [48]. (2) IL-4Rα operates in airway epithelial cells, whereas SIK3 primarily targets AT2 cells. Consequently, STUB1 may provide protective effects against the potential pathogenicity of stress-induced injury in various lung cells through the modulation of different substrates. The existing data also suggest a functional diversity of STUB1 in conjunction with its various substrates across distinct cell types. To be noted, our data have also shown that pulmonary STUB1 was not expressed in endothelial cells (Fig. 1i), which was contrary to the previous reports regarding STUB1 expression in various cancers. Two possible reasons may explain this discrepancy: (i) The endothelial expression of STUB1 is mostly reported in cancer studies [16], raising the possibility that endothelial STUB1 may play a more important role in transformed cells than in non-transformed cells. (ii) Even for the same tissue type, STUB1 may exhibit a distinct expression pattern in different species. For example, STUB1 is expressed in pachytene spermatocytes, spermatogonia, differentiating spermatids and Sertoli cells (SCs) in the normal human testis, whereas it is exclusively expressed in SCs in the mouse testis [49]. Thus, this tissue and cell-type specificity in STUB1 expression may strengthen the point that it is critical to study causal links between STUB1 expression and its upstream regulation mechanisms in as many different organisms, tissues and cell types in the future, given that gene regulation is highly context dependent [50].

Given our observations on the effects of differential STUB1 levels on the magnitude of stress-induced lung injury and its potent impact on alveolar responses in vivo, we endeavored to understand the upstream regulation of STUB1 in AT2s. We therefore carried out a series of pharmacological and biochemical assays in two cell lines and uncovered oxidative stress as the most prominent hit: low levels of ROS stimulated STUB1 expression in a time-dependent manner, while high levels of ROS substantially inhibited STUB1 expression in AT2s (Fig. 4a, b). This is understandable since at high concentrations, ROS usually exhibit cytotoxicity, whereas at low levels, ROS can act as important cellular second messengers involved in various aspects of cell signaling [51]. On the other hand, although AT1s and AT2s play essential collaborative roles in maintaining the integrity of the air-blood barrier of alveoli, AT1s are the most vulnerable to oxidant damage in the alveolus because of their large surface area and the possibility of a reduced antioxidant capacity compared to AT2s. Accordingly, AT2s are well recognized as a key structure and defender in the lung but also are the targets in many lung diseases, including ARDS, ventilator-induced lung injury, and pulmonary fibrosis [52]. In this regard, specific expression of STUB1 in AT2s seemed to be subject to a delicate control by ROS signaling. In support, we further showed that low concentrations of H_2_O_2_ stimulated *STUB1* expression via promotion of Nrf2 transactivation (Fig. 4d). Activation of the Nrf2-antioxidant response element (ARE) signaling is well accepted as a core antioxidant means of the cell [53]. Accumulated data specifying the protective role of Nrf2 that may satisfactorily control the immune response to decrease lung injury by stabilizing host immune competence for lung repair [54, 55]. Of particular interest, Nrf2 has been shown to alleviate LPS-induced ALI by targeting various targets [56, 57]. Our findings extend these understandings by identifying STUB1 as a potent downstream effector of Nrf2 transcription. Additionally, the transactivation of Nrf2 turned out to be regulated by ERK through their direct physical interaction (Fig. 4e–k). The mammalian response to stress-induced injury often involves activation of MAPKs, which consist of extracellular signal-regulated protein kinase (ERK), c-Jun N-terminal kinase/stress-activated protein kinase (JNK/SAPK) and P38. However, the subfamilies are usually differentially affected by particular stimuli [58]. For example, exposure to H_2_O_2_ induces P38 activation and barrier dysfunction in lung microvascular endothelial cells [59]. In contrast, H_2_O_2_ treatment suppresses expression of testicular steroidogenic enzyme genes through JNK-inhibited Nur77 transactivation in Leydig cells [60]. Our findings, in accordance with previous reports demonstrating that ERK is required for Nrf2 phosphorylation and subsequent activation [61–63], showed that U0126, an inhibitor of ERK, but not JNK inhibitor SP600125 or P38 inhibitor SB203580, blocked H_2_O_2_-induced Nrf2 transactivation (Fig. 4e). Thus, we propose that distinct MAPK subfamilies may regulate cell survival in response to stress in a context-dependent manner. Furthermore, because the N-terminal domain of ERK2 profoundly affects ERK2 localization, MEK binding and kinase activity [64], it is therefore a logical observation that Nrf2 interacted with full-length ERK2 and ERK2 N-terminal region, but not with the C-terminal region (Fig. 4i). Additionally, we have convincingly demonstrated a bidirectional regulation of STUB1 by ROS: mild ROS potentiates Nrf2-mediated transactivation of STUB1 expression, while high-level ROS decreases STUB1 expression by directly impairing its protein half-life (Fig. 4). Low concentrations of intracellular reactive oxygen species (ROS) significantly contribute to redox signaling; however, elevated levels of ROS can lead to macromolecular damage, adversely affecting both translation and protein turnover. The oxidation of proteins can hinder their functionality, cause fragmentation, and encourage inappropriate interactions that may result in excessive protein proteolysis, ultimately leading to cellular death through apoptosis. Consequently, it is crucial to maintain low levels of protein oxidation to ensure balanced protein expression within the cell [65]. In this context, we propose that the degradation of STUB1 is likely a consequence of a general dysfunction in the cellular protein synthesis and stability mechanisms under conditions of high oxidative stress. Alternatively, elevated ROS levels might induce post-translational modifications on STUB1 itself, marking it for degradation, as post-translational modifications (PTMs), such as phosphorylation, acetylation and glycosylation, are known to influence proteolytic stability [66]. Furthermore, it is highly improbable that increased ROS levels would activate another E3 ligase responsible for the turnover of STUB1, given that numerous E3 ligases have been predicted to mediate the ubiquitination of STUB1 (http://ubibrowser.bioit.cn/ubibrowser_v3/Home/Result/index/name/Q9UNE7/module/Strict/proteinType/noE3), yet none have so far been substantiated by significant experimental evidence. Additionally, from a translational perspective, the connection between biomarkers of oxidative stress and patient outcomes offers significant prognostic insights that can inform clinical decision-making for ARDS. Elevated levels of ROS and oxidative stress indicators are strongly linked to extended hospital stays, greater reliance on mechanical ventilation, increased rates of ICU admissions, and higher mortality rates [67]. Assessing oxidative stress in ARDS patients by analyzing exhaled gases, such as nitric oxide (NO) and H_2_O_2_, serves as an effective method for real-time assessment [68]. In this context, a deeper understanding of the ROS/ERK2/Nrf2/STUB1 signaling pathway may contribute to the development of more effective strategies that show a significant correlation with the clinical features and disease progression in ARDS patients.

Salt-inducible kinases (SIKs), belonging to the AMP-activated protein kinase (AMPK) family, function mainly in regulating energy response-related physiological processes [69]. While SIK inhibitors exert clearly anti-inflammatory effects, the role of SIKs in inflammation remains complicated. On one hand, the proinflammatory cytokines are upregulated in *Sik3*-, rather than in *Sik1*-, and *Sik2*-KO mice. Consequently, *Sik3* KO mice died of infection following LPS treatment [70]. On the other hand, SIK3 induces pro-inflammatory arginine metabolism and suppresses anti-inflammatory enzymes in breast cancer cells [71]. Thus, whether SIK3 exhibits anti-inflammatory or proinflammatory phenotypes may be cell context-dependent. Our proteome-wide E3 ligase-substrate analysis, protein interactions assays in vitro and in vivo and LPS-induced ALI model suggested that STUB1 may induce atypical polyubiquitination of SIK3 in AT2s (Fig. 5). The STUB1-mediated SIK3 polyubiquitination seemed to be distinct from the previously described STUB1-elicited proteasomal degradation. Instead, STUB1-induced polyubiquitination of SIK3 potentiated its activation (Fig. 5i) and facilitated its binding to downstream substrates (Fig. 5j). One feasible explanation for this noncanonical polyubiquitination is the different topology of polyubiquitin chains controlled by STUB1: STUB1 preferentially facilitated the assembly of K63-mediated polyubiquitination of SIK3 (Fig. 5g). K63-linked polyubiquitination is well known to regulate interaction, translocation, and activation of target proteins [72]. This may help to explain, at least in part, why STUB1-induced polyubiquitination does not lead to SIK3 degradation. Despite the previously reported roles of STUB1 in conferring K48-mediated polyubiquitination of Foxp3 and promoting its degradation in regulatory T cells, we failed to detect an appreciable level of K48-mediated polyubiquitination of SIK3 and associated degradation by STUB1. It is thus reasonable to postulate that STUB1 may partner with a different E2 for distinct substrates in a cell-type-specific manner. K63-mediated polyubiquitination has been emerging as a critical post-translational modification for protein trafficking, kinase and phosphatase activation. This nonproteolytic atypical polyubiquitination is therefore essentially involved in cell survival and cancer development, such as in the cases of Akt and TAK1 [73]. In both cases, polyubiquitination of these kinases does not result in protein degradation but rather regulates kinase activation in a dose- or cell type-dependent manner. Our findings expand this knowledge by adding STUB1 as another intriguing example. On this basis, it is tempting to consider that the proteolysis-independent polyubiquitination may serve as a general mechanism by which certain kinases are regulated during cellular stress response.

There is mounting evidence that adeno-associated virus (AAV) is a highly efficient gene therapy vector that is utilized to deliver transgenes to the lung in vivo. Among various AAV serotypes, AAV6 is one of the most efficient serotypes used for lung gene transfer, and it efficiently transduces both airway and AT2s [74]. To this end, our mouse models of ALI involving intranasal instillation of a constitutive-active SIK3 mutant, namely pAAV6-CMV-Sik3 (T163E), have practical and potential therapeutic relevance because we have demonstrated that replenishment of activated SIK3 in vivo effectively protected against LPS-induced lung injury, even in the absence of STUB1 (Fig. 6). Due to the fact that the T163E mutant simulates phosphorylation and is consequently already in an active state, the beneficial effects of T163E on STUB1 deficiency-aggravated ALI may indicate that the T163E mutation circumvents the requirement for the polyubiquitination-dependent activation phase of SIK3. Alternatively, it is plausible that the polyubiquitination mediated by STUB1 aids in SIK3’s engagement with substrates such as CRTC2, a function that the T163E mutant might also enhance. Supporting this latter theory, it has been demonstrated that the activation of SIK3 further increases its interaction with downstream substrates [75]. In summary, our in vivo rescuing assay demonstrates that the primary function of STUB1 is not solely to sustain SIK3 activity, but it also encompasses additional non-catalytic roles (for instance, scaffolding) that are compensated for by the hyperactive mutant T163E. From a translational standpoint, our findings imply that the reactivation of the downstream effectors of the STUB1 signaling pathway or using pharmacogenetic approaches may act as an alternative therapeutic option for the treatment of ALI, thus emphasizing the translational significance of such a local ROS/STUB1/SIK3 cascade in the delicate control of susceptibility and severity of ALI. In addition, significant advancements have been made in the clinical application of AAV over the past few decades, highlighting its immense potential in gene therapy for a diverse array of genetic and acquired disorders. To date, seven AAV gene therapy products have received FDA approval and entered the market; however, concerns persist regarding the safe application of viral therapies in humans, particularly related to immune responses and negative off-target effects such as genotoxicity, hepatotoxicity, thrombotic microangiopathy, and neurotoxicity [76–78]. In this context, to mitigate the potential off-target effects associated with AAV-SIK3 interactions, a combinatorial approach utilizing various strategies, including the use of extracellular vesicle (EV) encapsulated AAV delivery, optimization of AAV capsid and promoter design, enhancement of precision delivery methods, and the implementation of dosing optimization along with ALI patient stratification, can assist in achieving effective on-target gene editing, with (near-)zero levels of off-target *SIK3* gene editing [79].

Our study has some limitations. First, total SIK3 knockout mice have significant developmental issues [80]. These functional traits may complicate their use for studying the immune system in adult mice. Therefore, conditional deletion of SIK3 in AT2s is absolutely needed to further delineate the mechanistic details of SIK3’s actions during ALI. Second, there is a clear compensation among different SIK isoforms in vivo. Characterization of primary macrophages from the single and double knock-in (KI) mice has highlighted an integral role for SIK2 and SIK3 in innate immunity by regulating macrophage polarization [81]. Similarly, SIK1 and SIK3 are synergistically involved in the negative regulation of the TLR4-mediated signaling [82]. In this regard, while we have present data on the beneficial effects of SIK3 overexpression on ALI pathogenesis, our study does not address whether similar things may also happen to SIK1 and SIK2, and caution should be applied when interpreting and promoting these findings. Therefore, double/triple knockout experiments in the future would greatly deepen our understanding of the compensatory mechanisms at play, further strengthen the interpretability of our findings, and be warranted. Lastly, STUB1 is a well-recognized chaperone-associated E3 ligase that plays a crucial role in protein quality control, with a variety of known substrates. The deletion of STUB1 specific to AT2 may result in more extensive proteostasis issues, extending beyond the mere dysregulation of SIK3. Although our in vivo SIK3 rescuing assay provides strong evidence for the STUB1/SIK3 axis being a significant factor in ALI, the severity of the knockout phenotype may represent a composite effect. Consequently, the disruption of additional STUB1-dependent cellular processes might also play a role in the heightened vulnerability to ALI observed in the Stub1^fl/fl^/Sftpc-Cre^+/–^ mice, which warrants further exploration.

**Fig. 7:**
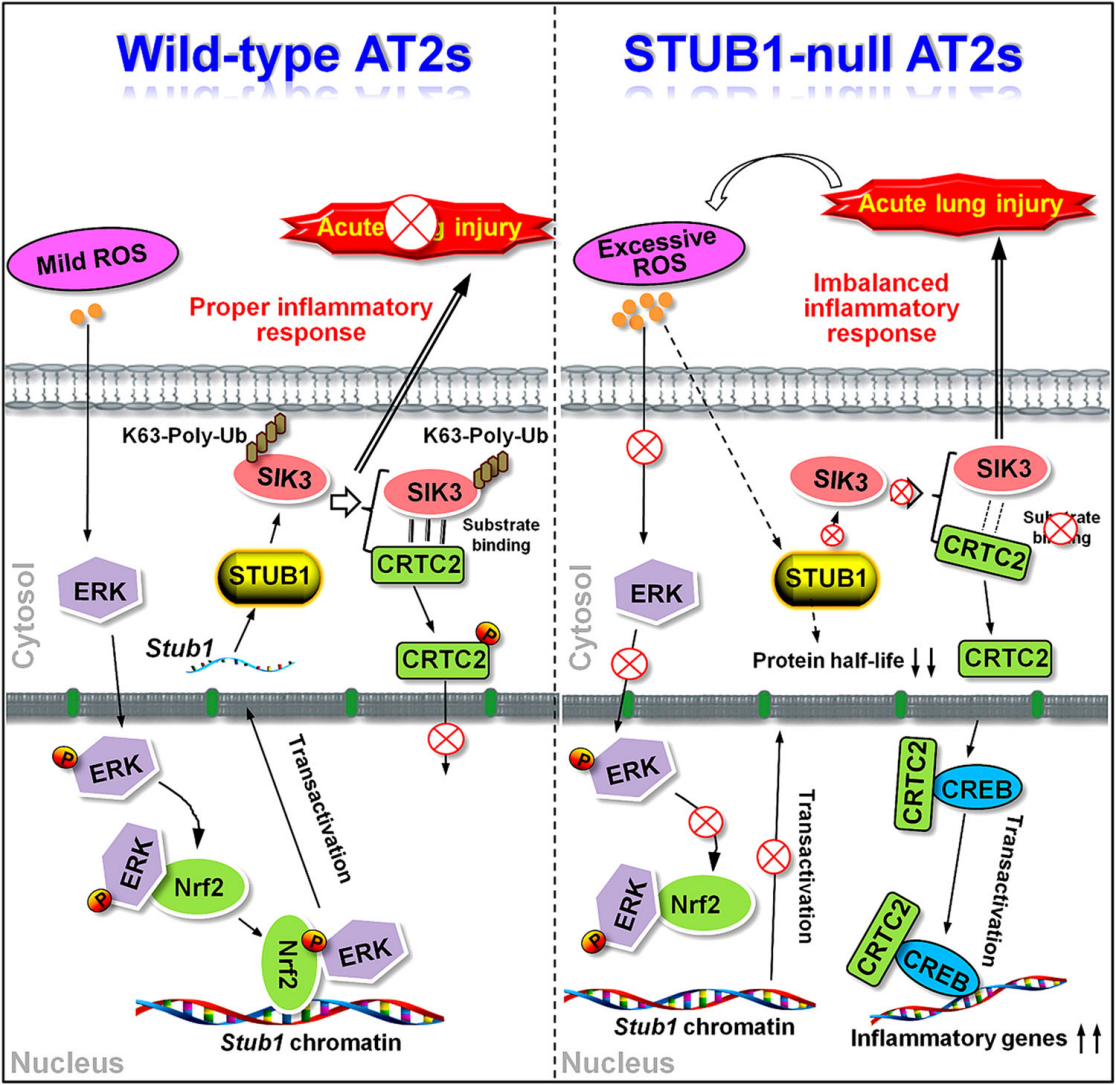
A proposed model illustrating STUB1-mediated nonproteolytic atypical polyubiquitination of SIK3, and its involvement in determining susceptibility and severity of ALI. A mild ROS potentiates Nrf2-mediated transactivation of the *STUB1* gene via activation of the ERK signaling. By contrast, aberrant redox homeostasis associated with ALI causes excessive production of ROS, which inhibits the protein half-life of STUB1. The resultant STUB1 deficiency fails to conferring K63-mediated nonproteolytic polyubiquitination of SIK3, which eventually abrogates CRTC2 (CREB-regulated transcription co-activator 2) substrate binding for SIK3 and triggered CREB signaling-mediated proinflammatory phenotypes. As a result, disruption in SIK3 activation aggravates lung edema, augments inflammatory infiltrate, increases AT2 apoptosis, thus potentiating cell-intrinsic inflammatory potential during ALI.

In sum, our mechanistic and functional data position STUB1 as a critical determinant of SIK3 activation in AT2s. Aberrant redox homeostasis associated with ALI usually causes excessive production of ROS, which significantly decreases the protein half-life of STUB1. The resultant STUB1 deficiency failed to confer K63-mediated nonproteolytic atypical polyubiquitination of SIK3, which in turn abrogated substrate binding of CRTC2 by SIK3, reduced phosphorylation of CRTC2, promoted nuclear translocation of CRTC2, and thereby potentiated CREB-dependent transcription at inflammatory gene loci, eventually leading to proinflammatory phenotypes. Consequently, disruption in SIK3 activation aggravated lung edema, augmented inflammatory infiltrate, and increased AT2 apoptosis, all of which are characteristic features of pathogenesis and progression of ALI (Fig. 7). Simultaneously, our findings highlight that a more granular understanding of STUB1 signaling (or regulation thereof) will be obligatory to better exploit the anti-inflammation roles of SIK3 activation in AT2s, in combination with ALI care.

## Materials and methods

### Mice

All protocols involved in animal work, performed in accordance with the criteria from “*Guide for Care and Use of Laboratory Animals*”, were approved by the Institutional Animal Care and Use Committee (IACUC) of Air Force Medical University (Approval #: KY20173012). The *Stub1* floxed mice, kindly supplied by Dr. Yang Yang (First Affiliated Hospital of Xi’an Jiaotong University, China), had been backcrossed onto the C57BL/6 J background for at least eight generations. The *Sftpc*-Cre mice were obtained from Labbio Biotechnology (Chengdu, China). The Stub1^fl/fl^/Sftpc-Cre^+/-^ (KO) mice were generated by mating the floxed *Stub1* heterozygous female mice with *Sftpc*-Cre transgenic male mice [11]. Genotyping PCR using genomic DNA from tips of tails of 4-week-old pups was previously described [11], by using an EZ Fast Tissue/Tail PCR Genotyping Kit (EZ BioResearch, Saint Louis, MO, USA). Mice were housed in a specific pathogen-free environment, with a 12-h light/dark cycle and *ad libitum* access to food and water, and were fed with a standard Lab Mouse Maintenance Diet which consisted of 3.2% fat, 17.9% protein, 57.5% carbohydrate, and 3.9% fiber, as well as a mix of minerals and vitamins (Jiangsu Xietong Pharmaceutical Bio-engineering, Nanjing, China). Mice were allowed to acclimatize for at least seven days before experiments.

### Induction of lung injury

An intratracheal LPS-induced ALI model was established according to our previous work [83]. Briefly, mice (12 weeks old) were anaesthetized using a ketamine (80 mg/kg) and xylazine (8 mg/kg) mixture administered via intraperitoneal (i.p.) injection and placed on a temperature-controlled table (DLAB Scientific, Beijing, China), with the neck super-extended at a 90° angle relative to the table. We then vertically inserted a 22-gauge venous catheter approximately 10 mm into the trachea along the tongue’s root. Subsequently, mice were instilled intratracheally with LPS (5 μg/g B.W. dissolved in sterile PBS, Sigma-Aldrich, Shanghai, China) or the equivalent volume of PBS as a control for 24 or 48 h. A decrease in body temperature or the occurrence of respiratory depression was indicative of proper induction of ALI [83]. In separate experiments, Kaplan-Meier plots were determined for mouse survival following instillations of ALI-inducing lethal LPS (50 μg/g B.W.) [20].

Overventilation-induced lung injury was generated as described [21]. In brief, anaesthetized WT and KO mice were overventilated using a RoVent® Advanced Ventilator (Zenda LLC, Hong Kong, China) at 30 ml/kg (tidal volume/body weight) for 30 min. Mice receiving a low-tidal ventilation with an inspiratory pressure of 7 ml/kg were used as normal controls. Left lung ischemia/reperfusion (I/R) injury was performed as described elsewhere [84]. After cannulation, anesthetized mice were connected to a RoVent® Advanced Ventilator with room air at 0.28-ml tidal volume and 85-bpm respiratory rate. The animals were placed on their right sides so that a left anterolateral thoracotomy in the fifth intercostal space could be performed. The left hilum was cross-clamped for 60 min, after which the cross-clamp was released, and reperfusion proceeded for another 60 min. Following I/R induction, plasma PaO_2_ concentrations were measured in deeply anesthetized mice using a MouseOx® oximeter (Starr Life Sciences, Oakmont, PA, USA).

### Lung tissue morphology and edema detection

After tissue harvest, fresh left lung tissues were immediately fixed in 10% formalin, embedded in paraffin, sectioned at 4 μm on a rotary microtome (Leica, Beijing, China), and stained with H&E. Lung injury was assessed in 4 categories: interstitial inflammation; neutrophil infiltration; congestion; and edema. The results were shown as scores on a 0‒4-point scale: no injury = 0; injury in 25% of the field = 1; injury in 50% of the field = 2; injury in 75% of the field = 3; and injury throughout the field = 4. Four microscopic fields from each slide and five slides from each animal were analyzed. The sums of tissue slides were averaged to evaluate the severity of lung injury [85]. All microscopic sections were interpreted in a blinded manner by a pulmonary pathologist. In separate experiments, left lungs were dissected free of nonparenchymal tissue, and then the lung wet weight was measured. Following bronchoalveolar lavage, the same lung was dried at 56 °C for 72 h, followed by calculation of the ratio of wet/dry lung weight [83].

### Cell counts, protein concentrations and cytokines in bronchoalveolar lavage fluid (BALF)

Left lungs were triply lavaged with 4 ml of PBS containing 10 nM of ethylenediamine tetraacetic acid (Sigma-Aldrich, Beijing, China) via tracheotomy. ~1.2 ml of total BALF was collected from each mouse and centrifuged at 4 °C at 1500 × *g* for 15 min. The resultant cell pellets were stained with May-Grunwald-Giemsa (Solarbio, Beijing, China) as instructed by the manufacturer, followed by determination of total cells, as well as macrophage, lymphocyte and neutrophil numbers, using a Neubauer chamber (Thomas Scientific, Shanghai, China). In another experimental setting, total protein concentrations and cytokine levels in cell-free supernatants from lavaged BALF were assessed using a bicinchoninic acid protein assay kit (Sigma-Aldrich) and Mouse IL-1β/IL-6/TNF-α ELISA Kits (Beyotime, Shanghai, China), respectively, according to the manufacturers’ instructions.

### Determination of lung oxidant status

Left lungs were homogenized in ice-cold PBS buffer. The homogenates were centrifuged at 3000 × *g* for 10 min. The resultant supernatants were then subjected to protein concentration quantification using the Pierce™ bicinchoninic acid (BCA) Protein Assay Kit (Thermo Scientific). The superoxide dismutase (SOD) and malondialdehyde (MDA) contents in lung tissues were finally measured by spectrophotometry at 450 and 532 nm, respectively.

For determination of dihydroethidium (DHE) intensity, eight-micrometer frozen sections from left lung tissues were rinsed with deionized water (diH_2_O) for 20 seconds to remove optimal cutting temperature (OCT) compound, followed by incubation in DHE staining solution (Sigma-Aldrich DHE dissolved in Milli-Q pure H_2_O, 5 μM) at room temperature in the dark for 20 min. Following sequential diH_2_O wash and DAPI counterstaining, sections were immediately subjected to fluorescence observation and image capture under an Axio Imager M1 microscope (Zeiss, Beijing, China). The relative DH signal intensity was calculated and exported as the red 2-OH-E^+^ positive-cells per 10^3^ cells.

### Cell treatment

Isolation and purification of murine alveolar epithelial cells (ATs) was carried out according to previously reported protocols [86, 87]. Briefly, lungs of anaesthetized mice were rapidly perfused via the pulmonary artery with 10 ml PBS at 23 °C. About 2ml of dispase was instilled into the lung through the trachea, followed by 0.5ml of melted agarose (2% in PBS). After lungs were collected from mice using forceps, tissues were immediately lavaged for five times at 37 °C with HEPES-buffered DMEM (containing 0.9% NaCl, 0.1% glucose, 30 mM HEPES, 6 mM KCl, 0.1 mg/ml streptomycin sulfate, 0.07 mg/ml penicillin G, 0.07 mg/ml EGTA, 3 mM Na_2_HPO_4_, 3 mM NaH_2_PO_4_, pH 7.4, 3 mM MgSO_4_ and 1.5 mM CaCl_2)_, followed by sequential mincing with a tissue scissors and digestion in HEPES-buffered DMEM containing 3 U/ml elastase (Thermo Scientific) for 10 min. The elastase digestion process was repeated twice. The resultant cell suspension was treated with 100 μg/ml DNase I (Sigma-Aldrich) at 37 °C for 5 min, and filtered through a 70-μm cell strainer and then a 20-μm nylon gauze to obtain a single-cell suspension in a 15-ml centrifuge tube. The cells were incubated in mouse IgG-coated polystyrene bacteriological 100 mm Petri dishes at room temperature for 30 min, centrifuged at 250 × *g* for 5 min to remove unattached cells, and resuspended in HEPES-buffered DMEM containing 1% FBS and 100 μg/ml DNase I. To remove potential macrophage and AT1 contaminations, cells were incubated with mouse IgG and rabbit anti-mouse AT1α (40 μg/ml) at 4 °C for 30 min respectively, followed by incubation with goat anti-mouse IgG Dynabeads (100 μl/mouse) and goat anti-rabbit IgG BioMags beads (500 μl/mouse, both beads from Thermo Scientific). To isolate AT1s, lung tissues were processed as described above, and the resultant single cell suspension was incubated with rabbit anti-mouse AT1α (40 μg/ml) at 4 °C for 30 min, and washed thoroughly using HEPES-buffered DMEM. AT1s were finally purified from contaminated cells by incubation with goat anti-rabbit IgG BioMags beads (500 μl beads/mouse) at 4 °C for 15 min. The purity of isolated ATs was examined by general immunofluorescence staining using antibodies against AT1α or SP-C.

The human lung adenocarcinoma A549 cells (Cat.#: CRM-CCL-185), human pulmonary artery endothelial cells (HPAEC, Cat.#: PCS-100-022), human lung microvascular endothelial cells (HULEC-5a) and human umbilical vein endothelial cells (HUVEC, Cat.#: PCS-100-013) were all obtained from American Type Culture Collection (ATCC, Manassas, VA, USA). A549 cells were maintained in DMEM supplemented with 10% fetal bovine serum (FBS) and 2 mM L-glutamine (Thermo Scientific). HPAEC were cultured in vascular cell basal medium supplemented with endothelial cell growth kit-VEGF (ATCC). HULEC-5a were maintained in antibiotic-free endothelial basal medium (Sigma-Aldrich) with 10% FBS. HUVEC were maintained in endothelial cell medium (ECM, Sigma-Aldrich) containing 10% FBS and endothelial cell growth supplement (ECGS), and 100 U/ml of penicillin and streptomycin. All cells were grown in 5% CO_2_ in an air-humidified incubator at 37 °C, and were passaged when they were ∼80% confluent. To induce anoxia and reoxygenation (A/R) injury, A549 cells were cultured in an anoxic chamber with 5% CO_2_ and 95% N_2_ at 37 °C for 2 h, followed by reoxygenation for another 2 h [21].

To study the potential involvement of moderate oxidative stress in the regulation of STUB1 expression, A549 cells were incubated with different doses of H_2_O_2_ for various durations as indicated. To investigate the specific involvement of MAPK signaling in mediating ROS-elicited STUB1 expression, cells were preincubated with the JNK inhibitor SP600125 (50 μM), P38 inhibitor SB203580 (10 μM), or MEK (ERK upstream signal molecule) inhibitor U0126 (5 mM, all inhibitors were from MedChemExpress, Shanghai, China) for 30 min before H_2_O_2_ challenge. To study the effects of LPS challenge, A549 cells (2 × 10^6^ cells/ml) were incubated in DMEM containing 100 ng/ml LPS for 12 h.

### Plasmids and transfection

The pCDNA3.1-Flag-Nrf2 was a gift from Randall Moon (Addgene plasmid #36971) [88]. The pBABE-mRFP1-NRF2 and pBABE-mRFP1-NRF2 (C506S, L4A) were gifts from Kevin Janes (Addgene plasmid #136579 and #136580) [30]. His-Stub1 was created by inserting the whole coding region into the pcDNA3.1-His vector (Thermo Scientific), and Stub1 point mutant, H260Q, was generated with the GeneEditor in vitro site-directed mutagenesis system (Promega, Shanghai, China), as reported [89]. pCMV6-Myc-Sik3 was purchased from OriGene (Wuxi, China). Generation of lentiviral pHAGE-MEK1CA expressing a constitutive active form of the kinase MEK1 (MEK1CA) acting upstream of ERK1/2 was described elsewhere [90]. Mammalian expression constructs of pRK5-HA-Ubiquitin-WT and pRK5-HA-Ubiquitin^K0^, as well as other ubiquitin mutants, were all obtained from AddGene. For bacterial expression, *Stub1* PCR product, along with different ERK2 mutant PCR products, including ERK2-F (1-358 aa), ERK2-Δ1 (1-271 aa) and ERK2-Δ2 (54-358 aa), were amplified using reported primers [89, 91], and were digested with BamHI and XhoI and subcloned into pGEX-6P-1 vector (Cytiva, Shanghai, China). For mammalian cell expression, PCR products of ERK2-F (1-358 aa) were subcloned into pcDNA3.1(+) vector (Cytiva).

Stable knockdown of STUB1 in A549 cells was achieved by using the pLKO.1-EGFP-based lentiviral transduction system [92]. Three shRNA sequences targeting *STUB1* were: Sh1: CCGGGACGCATTCATCTCTGAGAATCTCGAGATTCTCAGAGATGAATGCGTCTTTTTG and AATTCAAAAAGACGCATTCATCTCTGAGAATCTCGAGATTCTCAGAGATGAATGCGTC; Sh2: CGGGCAGTCTGTGAAGGCGCACTTCTCGAGAAGTGCGCCTTCACAGACTGCTTTTTG and ATTCAAAAAGCAGTCTGTGAAGGCGCACTTCTCGAGAAGTGCGCCTTCACAGACTGC; Sh3: CGGCGCGAAGAAGAAGCGCTGGAACTCGAGTTCCAGCGCTTCTTCTTCGCGTTTTTG and ATTCAAAAACGCGAAGAAGAAGCGCTGGAACTCGAGTTCCAGCGCTTCTTCTTCGCG. *STUB1* targeting shRNA or non-target scramble shRNA (Scramble sh) were synthesized by Sangon Biotech (Shanghai, China) and cloned into the pLKO.1-EGFP vector (QCheng Bio, Shanghai, China) using AgeI and EcolI restriction sites. To prepare lentivirus, pLKO.1-EGFP-STUB1 (three shRNAs mixture) or Scramble sh, along with the packing vector psPAX2 and pMD2G (both from Addgene), were co-transfected into 293 T cells using Lipofectamine 3000 (Thermo Scientific) following vendor’s instructions. After the collection of lentivirus particles from the culture medium of 293 T cells, A549 cells were infected for 48 h. The A549 cells stably deprived of STUB1 were then selected by incubation with 50 ng/ml puromycin (Sigma-Aldrich) accordingly. Nrf2 shRNA and control Scramble shRNA plasmids were obtained from Santa Cruz Biotechnology (Shanghai, China). To knock down the endogenous Nrf2, confluent A549 cells were transfected for 48 h with the indicated shRNA using Lipofectamine 3000 as instructed by the manufacturer.

### Quantitative real-time PCR (qPCR)

Total RNA was isolated from cells or lung tissues using the Qiagen miniRNA extraction kit (Qiagen, Shanghai, China) following the manufacturer’s instructions. RNA was quantified using a NanoDrop 2000 spectrophotometer (Thermo Scientific), and complementary DNA was reverse transcribed using the Thermo Scientific RevertAid RT Kit as instructed by the manufacturer. The cDNA samples were used at 20 ng/well in a 96-well plate and run in triplicate. Subsequent PCR reactions were set up in 25-μl volumes using SYBR® Green Master Mix (Thermo Scientific) on an ABI Prism 7500 Sequence Detection System (Thermo Scientific). Quantification of relative mRNA expression was performed by the comparative CT (critical threshold) method, where the amount of target mRNA, normalized to endogenous *18S* rRNA expression, was determined by the formula 2^–ΔCT^ [93]. The primers used in the current study were listed in Supplementary Table 2.

### Antibody pre-absorption and immunoblotting

Where appropriate, pre-absorbed serum was prepared by incubating the anti-STUB1 antibody with the STUB1 peptide antigen (R&D Systems, final concentration of peptide 5.0 μg/ml) for 12 h at 4 °C while rotating, after which the antibody was collected following mild centrifugation (900 × *g*).

Cells were lysed in RIPA lysis buffer containing 50 mM/L Tris-HCl (pH 8.0), 150 mM/L NaCl, 0.5% sodium deoxycholate, 0.1% sodium dodecylsulfate (SDS), 1% NP-40, 1 mM/L phenylmethanesulfonylfluoride and a halt phosphatase inhibitor cocktail (Roche, Shanghai, China). Left lung tissues were homogenized in Tissue Extraction Reagent (Thermo Scientific). The homogenates were incubated on ice for 45 min and then centrifuged at 4 °C at 12,000 × *g* for 5 min. The protein concentrations in supernatants were then determined using the Pierce™ BCA Protein Assay Kit (Thermo Scientific). Approximately 30 µg of total protein was loaded on an SDS polyacrylamide gel to complete electrophoresis at 120 V for 2 h, followed by being transferred onto a polyvinylidene difluoride (PVDF) membrane (Sigma-Aldrich). Blots were then incubated with primary antibodies overnight at 4°C, followed by detection with horseradish peroxidase-conjugated secondary antibodies, as listed in Supplementary Table 3. Targeted proteins were finally visualized using Pierce™ ECL Western Blotting Substrate Chemiluminescent system. The blots were analyzed with QUANTITY ONE v4.6 software. The data normalized to β-ACTIN was expressed as OD integration.

### Histological analysis

Immunohistochemistry was performed as described [46]. Briefly, 5-μm-thick 4% paraformaldehyde (PFA)-fixed lung sections were deparaffinized, rehydrated, and subjected to sequential antigen retrieval (20 min at 95 °C in 10 nM citrate buffer, pH=6.0) and elimination of endogenous peroxidase activity (20 min at room temperature in 0.5% v/v H_2_O_2_/methanol). Followed by thorough washing with PBS, sections were incubated at 4 °C overnight with different primary antibodies (Supplementary Table 3). Subsequent treatment with the second antibody and avidin–biotin complex (ABC) was carried out using VECTASTAIN ELITE® ABC Kit (Vector Lab, Burlingame, CA, USA). Final peroxidase reaction was achieved by incubating sections with 0.7 mg/ml 3-3’-diaminobenzidine tetrahydrochloride (Sigma-Aldrich) in a urea hydrogen peroxide solution (1.6 mg/ml). Slides were counterstained slightly with hematoxylin for 30 s to visualize nuclei.

For double immunofluorescence, after deparaffinization and rehydration, 4% PFA-fixed lung sections were incubated with blocking solution (10% donkey serum, 0.5% BSA and 0.3% Triton X-100 in PBS) at room temperature for 1 h. Sections were then incubated with different primary antibodies as indicated (Supplementary Table 3), in a humidified chamber overnight at 4 °C. Slides were rinsed 3 times with diluent and incubated for approximately 1 h at room temperature with fluorescein isothiocyanate (FITC)/Cy3-labeled second antibodies (Supplementary Table 3). Sections were then washed 3 times with diluents, counterstained for 5 min with 2-(4-Amidinophenyl)-6-indolecarbamidine dihydrochloride (DAPI, Sigma-Aldrich), and were viewed by epifluorescence with an Axio Imager M1 microscope (Zeiss, Beijing, China) and scanned with a Zeiss LSM880 laser scanning confocal microscope. Sections incubated with a preabsorbed serum, instead of the primary antibody, served as negative controls.

### Measurement of apoptosis in vivo

In situ cell death was evaluated by TUNEL staining as described. Following deparaffinization and rehydration, 4% PFA-fixed lung sections were subjected to antigen retrieval as above-mentioned. Sections were then saturated with 50 μl of an equilibration buffer (EB: 200 mM K-cacodylate, 25 mM Tris-HCl (pH 6.6), 0.2 mM DTT, 0.25 μg/μl BSA and 2.5 mM CoCl_2_) at room temperature (RT) for 10 min, followed by incubation with TUNEL reaction mixture and 1 μl of 25 U/μl of terminal deoxynucleotidyl transferase (Roche) at 37 °C for 1 h. TUNEL reaction was terminated by incubation with 2 × sodium chloride and sodium citrate (2×SSC: 300 mM NaCl and 30 mM Na-citrate, pH 7.4) at RT for 15 min. After a thorough rinse with PBS, the dark sections were counterstained for 5 min with DAPI. Positive signals were finally visualized by epifluorescence with an Axio Imager M1 microscope. For quantification of TUNEL staining, TUNEL-positive nuclei in at least 5 regions of each slide were manually counted, and a total of seven mice from each group were analyzed [93].

### Assessment of cell viability, lactate dehydrogenase (LDH) cytotoxicity and apoptosis in A/R cell model

The A549 cells with different transfections as indicated were cultured in an anoxic chamber with 5% CO_2_ and 95% N_2_ at 37°C for 2 h, followed by reoxygenation for another 2 h to induce A/R. Upon cell harvest, cell viability and cytotoxicity were assayed by spectrophotometry using a CyQUANT MTT Cell Viability Kit (Thermo Scientific) and an LDH Cytotoxicity Assay Kit (Yeasen, Shanghai, China), respectively, following the vendors’ instructions. Final absorbance was read on a standard microplate reader (#680; Bio-Rad, Shanghai, China), using 570 nm and 490 nm as the primary wavelengths for MTT and LDH, respectively. Following A/R treatment, cytoplasmic histone-associated DNA fragments were quantitatively measured in different cells using an Apoptosis ELISA kit (Roche) according to the manufacturer’s instructions. Final absorbance was determined at 405 nm in triplicate spectrophotometry on a microplate reader (Bio-Rad).

### Luciferase reporter assay

The genomic fragments of human *STUB1* promoter ( ~ 2.3 kb upstream of the translation start site) were amplified from a universal human cDNA (Takara Bio, Beijing, China), and were subcloned into pGL3-Basic vector (Promega, Madison, WI, USA) using the CloneJET PCR Cloning Kit (Thermo Scientific). The pGL3-STUB1-Mu mutant was constructed by standard molecular biology techniques, with the aid of the Q5 Site-Directed Mutagenesis Kit (New England Biolabs, Ipswich, MA, USA). For reporter assay, 0.5 μg pGL3-STUB1 and pRL-TK Renilla reporter plasmid, along with 0.5 μg of Flag-Nrf, pBABE-Nrf2 or pBABE-Nrf2 (C506S, L4A), were co-transfected into Nrf2-null 293 T cells (Applied Biological Materials) using Lipofectamine 3000. 48 h after transfection, cells were challenged with different doses of H_2_O_2_ as indicated for 6 h, in the presence or absence of 0.5 mM NAC (Beyotime) [46], followed by measurement of luciferase activities using a dual luciferase reporter assay kit (Promega).

### Immunoprecipitation (IP) and Co-IP

Protein immunoprecipitation was performed as described [94]. Transfected cells were lysed in lysis buffer (50 mM Tris-HCl, pH 7.5, 150 mM NaCl, 1 mM EDTA, 1 mM EGTA, 1% Triton X-100, 0.5% NP-40, 1 mM Na_3_VO_4_, 2 mM NaF, 1 mg/ml aprotinin, 1 mM PMSF, and 10 mM N-ethylmaleimide NEM). The resultant lysates were centrifuged at 13,000 × *g* for 15 min to remove any insoluble material, and were then incubated with different antibodies at 4 °C overnight. Immunocomplexes were finally collected using protein A/G-Sepharose beads or Ni-NTA beads (Abcam, Shanghai, China), via extensive washing in lysis buffer. Of note, immunoprecipitation was performed under denaturing conditions in the case of detecting SIK3 polyubiquitination. To denature proteins, the lysates were added with SDS to a concentration of 2%, and then heated at 95 °C for 10 min. The lysates were subsequently diluted using lysis buffer to reduce SDS concentration to 0.2% before anti-SIK3 or anti-Ub antibody was added, followed by immunoblotting analysis as described above.

For the Co-IP assay, A549 cells were starved in DMEM containing 5% charcoal-stripped FBS overnight, and were then treated with 0.1 mM H_2_O_2_ for 6 h. After cell harvest, cells were lysed using RIPA lysis buffer (Thermo Scientific). The resultant whole cell lysates were incubated with 10 μg of anti-Nrf2 antibody for 12 h at 4 °C and incubated for another 6 h after addition of 30 μl protein A–agarose bead slurry (Abcam). The beads were then washed at 4 °C thoroughly using RIPA cell lysis buffer, and bound proteins were separated by SDS–PAGE and analyzed by immunoblotting using antibodies as indicated.

### Preparation of Glutathione S-transferase (GST) fusion protein and in vitro GST pull-down assay

GST fusions were expressed in log phase BL21 Star *Escherichia coli* (DE3) (Thermo Scientific) and induced with 0.01-0.2 mM isopropyl-1-thio-β-D-galactopyranoside (IPTG, Sigma-Aldrich) at 30 °C for 3–4 h or room temperature [91]. Bacterial pellets were resuspended in 1×PBS and lysed by probe sonication. The sonicates were clarified by centrifugation at 4 °C for 15 min at 13,000 × *g*, aliquoted, and stored at –80 °C. Levels of expressed GST fusions were estimated by incubating sonicates with glutathione-Sepharose beads (Abcam), washing, and then eluting with Elution Buffer (15 mM reduced glutathione, 100 mM Tris-HCl, pH 8.0, 120 mM NaCl), followed by SDS-PAGE. For GST-pull-down, BL21 Star *Escherichia coli* (DE3) was transformed with pGEX-6P-1 and pGEX-6P-1-ERK-2 (full-length and deletion mutants), and was induced with 0.1 mM IPTG for approximately 3–4 at 30 °C. Cells were then lysed, sonicated and centrifuged at 20,000 × *g* for 30 min. The resultant supernatants were incubated with glutathione-Sepharose beads overnight at 4 °C. Ten microliters of protein-bound beads were then incubated with HNTG buffer (25 mM HEPESKOH pH 7.5, 100 mM NaCl, 0.1% TritonX-100, 10% glycerol, 5 mM MgCl_2_, 1 mM DTT, 1 mM PMSF, and 5 mM EDTA). The recombinant protein Flag-Nrf2 was obtained by transfecting 293 T cells, followed by affinity purification with anti-Flag beads (Thermo Scientific). Flag-Nrf2 (100 ng) was then added and incubated at 4 °C for another 2 h, followed by washing three times with IP buffer, SDS-PAGE and immunoblotting analyses.

### In vitro polyubiquitination

10 μg of purified GST-STUB1 recombinant protein was mixed with 1 μg of GST-SIK3 recombinant protein corresponding to the C terminus of human SIK3 (amino acids 1-307, Thermo Scientific), along with E1 (25 ng), E2 (UbcH5b; 400 ng, R&D Systems), 2 μg of HA-ubiquitin or lysine lacking variant (HA-Ubiquitin^K0^), in energy regenerating solution and Ubiquitin conjugation reaction buffer (Enzo Life Sciences, Shanghai, China) [95]. Samples were incubated at 37 °C for 4 h, terminated by boiling for 5 min in SDS-sample buffer, and resolved by SDS-PAGE followed by immunoblotting using anti-SIK3 to monitor polyubiquitination of SIK3.

### Non-radioactive electrophoretic mobility shift assay (EMSA)

A549 cells were transfected with Flag-NRF2 using Lipofectamine 3000, in the presence or absence of cotransfection with pcDNA3.1( + )-ERK2-F. 48 h later, cells were treated with 0.1 mM H_2_O_2_ for another 6 h. Subsequently, cells were harvested, and nuclear protein extracts were prepared using the Nuclear Extraction Kit (Abcam) following the vendor’s protocol. To test direct binding of NRF2 to the antioxidant response element (ARE), 1 μg of NRF2-enriched nuclear extracts was incubated with 30 mM of custom DIG-labeled probes corresponding to the putative ARE motifs. Upon completion of gel electrophoresis and nylon membrane transfer, protein-bound DIG-labeled probes were immunologically detected with anti-DIG–alkaline phosphatase conjugate and CSPD chemiluminescent substrate (Roche), as per the manufacturer’s instructions. The oligonucleotide probe used was TCAGCGAAGCAAGTGAGGCATCTCACTGGGAAAGTC. Competitive inhibition consisted of incubation with a 50-fold molar excess of unlabeled/cold probe.

### Kinase activity assay

293 T cells were cotransfected with pcDNA3.1-His-Stub1 and pCMV6-Myc-Sik3 using Lipofectamine 3000. On the 4th day after transformation, cells were harvested and treated with LPS (100 ng/ml) for another 12 h. The Myc fusion protein was then immunoprecipitated using Anti-Myc Magnetic Beads (Thermo Scientific), followed by measurement of in vitro kinase activity of Myc-SIK3 proteins using HDAC5tide as a substrate, with the aid of the ADP-Glo™ Assay Kit (Promega).

In separate experiments, an in vitro kinase assay was performed to test the ERK2 mutants for their capability to phosphorylate myelin basic protein (MBP, a frequently used substrate for ERK) [96]. In brief, 100 ng of GST-ERK2-F, ERK2-△1 or ERK2-△2, in the presence or absence of the cotreatment with 10 ng of recombinant active MEK1 (Abcam) to activate the recombinant EER2 variants, were incubated with 3 μg MBP (Santa Cruz Biotechnology) in kinase reaction buffer (10 mM MgCl_2_, 1 mM DTT and 50 mM Tris-HCl pH 7.5) supplemented with 25 μM ATP and [γ-^32^P]-ATP (1 μCi per reaction) at 30 °C for 30 min. The reaction was terminated by adding SDS loading buffer. After the reaction, phosphate incorporation was visualized using autoradiography. After electrophoresis, the gels were stained with Coomassie Brilliant Blue as loading controls.

### Adeno-associated virus (AAV) preparation and administration in vivo

The mutation (Thr to Glu at T163) was introduced into mSik3 using site-directed mutagenesis as described [39], with the aid of a Q5® Site-Directed Mutagenesis Kit according to the vendor’s instructions. The mutant *Sik3* (T163E) was amplified by PCR and subcloned into the pAAV6-CMV (Cell Biolabs, San Diego, CA, USA) under the control of the cytomegalovirus (CMV), a potent promoter in rodents [97]. The pAAV6-CMV-Sik3 (T163E) was synthesized by Beijing Biomed Gene Technology. For AAV packaging and purification (Supplementary Fig. S14), recombinant AAVs were packaged in 293AAV cells (Cell Biolabs), which had been shown to have no mycoplasma contamination [98], by cotransfection with pAAV6-CMV-Sik3 (T163E), pAAV-RC2 and pHelper (all from Cell Biolabs) using Lipofectamine™ LTX (Thermo Scientific). 72 h later, cells and culture media were harvested and centrifuged at 1000 × *g* for 5 min, followed by removal of the supernatants. The resulting cell pellets were resuspended in lysis buffer (10 mM Tris-HCl, pH=7.6, 150 mM NaCl, 10 mM MgCl_2_) and repeatedly frozen and thawed by placing them alternately in a dry ice/ethanol bath and a water bath of 37 °C. Cell debris was removed by centrifugation at 10,000 ×g for 10 min. The virus-containing supernatants were then purified using a Cell Biolabs’ ViraBind™ AAV Purification Kit, according to the manufacturer's specifications. The titer of concentrated AAVs was determined using a Cell Biolabs’ QuickTiter™ AAV Quantitation Kit, and recombinant AAVs were finally formulated in sterile PBS containing 0.01% Pluronic F68 (Sigma-Aldrich) and stored in aliquots at −80 °C.

For AAV administration in vivo, anesthetized mice were placed in dorsal recumbency. A preliminary volume of 40 μl of vector was delivered drop-wise over the nares and passively inhaled, and a total of 80 μl of vector was instilled, as described elsewhere [99]. The AAV vectors were matched to a titer of 1 × 10^12^ genome copies (gc/ml) with sterile PBS. The negative control group was intranasally instilled with the same volume of an empty AAV vector. Mice were allowed to recover from anesthesia for 10 min in a recovery cage.

### Statistical analysis

Experiments were repeated at least three times, and one representative result from at least three similar results is presented. The significance of the results was determined using *Student’s t* test or two-way analysis of variance (ANOVA) followed by Tukey’s post-hoc test as appropriate, with the aid of GraphPad Prism 8 (GraphPad Software, San Diego, CA, USA). Statistical differences were considered significant at *P* < 0.05. Data were presented as the mean ± S.D.

## Supplementary information

Supplementary information

Supplementary Fig. 1

Supplementary Fig. 2

Supplementary Fig. 3

Supplementary Fig. 4

Supplementary Fig. 5

Supplementary Fig. 6

Supplementary Fig. 7

Supplementary Fig. 8

Supplementary Fig. 9

Supplementary Fig. 10

Supplementary Fig. 11

Supplementary Fig. 12

Supplementary Fig. 13

Supplementary Fig. 14

Supplementary Fig. 15

Uncropped immunoblots

## Data Availability

All data generated or analyzed during this study are included in this published article and its supplementary information files.
