# Supplementary material for: STUB1-induced polyubiquitination of SIK3 in alveolar type 2 epithelial cells alleviates severity and outcomes of acute lung injury
Source: Cell Death Dis. 2026 May 4;17(1):589. doi: 10.1038/s41419-026-08822-x (PMC13284247; doi:10.1038/s41419-026-08822-x)
Supplement: Supplementary file 1 — Supplementary information [file 41419_2026_8822_MOESM1_ESM.docx]

Supplementary figure legends

Supplementary Fig.1

At 24 h after lipopolysaccharide (LPS) challenge, bronchoalveolar lavage fluid (BALF) was isolated from sham control or LPS-treated mice, and was the subjected to qPCR analysis using specific primers to macrophages (*Cd38*), neutrophils (*S100a8*), or to B lymphocytes (*Cd19*). n = 4 in each group, **P* < 0.05 and *****P* < 0.0001 (*Student's t*-test). Right panel, myeloperoxidase (MPO) activity in lung tissues was determined at 24 h after ALI induction (n=7, *****P* < 0.0001, *Student's t*-test).

Supplementary Fig. 2

Purities of type I alveolar epithelial cells (AT1s) and AT2s. Cytocentrifuged AT2s and AT1s preparations were double-labeled with anti-SP-C antibodies/FITC anti-mouse IgG and anti-AT1α antibodies/Cy3 anti-goat IgG. Scale bar 25 μm.

Supplementary Fig. 3

Schematic strategy of specific knockout of Stub1 in AT2s using the Cre-loxP system. The primer sets, P1 and P2, P1 and P3, were used for genotyping.

Supplementary Fig. 4

Myeloperoxidase (MPO) activity in lung tissues was determined at 24 h after ALI induction (n=7, ****P* < 0.001 and *****P* < 0.0001, two way ANOVA followed by Tukey’s post-hoc test).

Supplementary Fig. 5

Parallel experiments were conducted to exclude possible effects of Cre expression in the lung. (A) Haematoxylin and eosin (H&E) staining of lung sections derived from sham control or lipopolysaccharide (LPS)-challenged WT or Sftpc-Cre^+/-^ mice at 24 h post-treatment. Bar=25 μm. Right panel, Morphometric analyses of lung by lung injury scores on a 0‒4-point scale in all groups as in (a), n = 7 in each group, *****P*< 0.0001 (two way ANOVA followed by Tukey’s post-hoc test). (b) Lung oedema was measured as the wet/dry weight ratio of the excised lung from WT or Sftpc-Cre^+/-^ mice 24 h after intratracheal administration of PBS or LPS, n=7/group, ****P*< 0.001 (two way ANOVA followed by Tukey’s post-hoc test). (c) Levels of IL-1β, IL-6, and TNF-α in BALF from mice 24 h after intratracheal administration of PBS or LPS (5 μg/g) as determined by ELISA, n=7/group, ***P*< 0.01, ****P*< 0.001 and *****P*< 0.0001 (two-way ANOVA followed by Tukey’s post-hoc test). (d) Representative 4% paraformaldehyde (PFA)-fixed lung sections stained by TUNEL demonstrated equal apoptosis in Sftpc-Cre^+/-^ mice at 24 h after LPS exposure (Bar=25 μm). Right panel, quantification of TUNEL–positive cells per field in LPS-treated WT or Sftpc-Cre^+/-^ mice at 24 h after LPS exposure (n=7, *Student's t*-test).

Supplementary Fig. 6

A549 cells were treated with 0.5 mM H_2_O_2_ for different time points, followed by cell viability measurement by spectrophotometry using a CyQUANT MTT Cell Viability Kit (Thermo Scientific).

Supplementary Fig. 7

Schematic of a consensus antioxidant response element (ARE) in the *STUB1* promoter.

Supplementary Fig. 8

Nrf2-null 293T cells were cotransfected with 0.5 μg of Flag-Nrf2, as well as the pGL3-STUB1 or pGL3-STUB1-Mu reporter plasmids as indicated. H_2_O_2_ (0.1 mM) was applied after 48 h of transfection, in the presence or absence of co-treatment with the ROS scavenger N-Acetyl-L-cysteine (NAC), and luciferase activity was measured after another 6 h-incubation. The error bars indicate the standard deviation (n=4/group, **P*<0.05 and *****P*<0.0001, one-way ANOVA followed by Tukey’s post-hoc test).

Supplementary Fig. 9

Quantification of the relative expression of different proteins in Fig. 4 by Image J (n=3, **P* < 0.05, ***P*< 0.01, ****P*< 0.001 and *****P*< 0.0001, two-way ANOVA followed by Tukey’s post-hoc test).

Supplementary Fig. 10

In vitro kinase assay: 100 ng of GST-ERK2-F, ERK2-△1 or ERK2-△2, in the presence or absence of the cotreatment with 10 ng of recombinant active MEK1 to activate the recombinant EER2 variants, were incubated with 3 μg MBP in kinase reaction buffer (10 mM MgCl_2_, 1 mM DTT and 50 mM Tris-HCl pH 7.5) supplemented with 25 μM ATP and [γ-^32^P]-ATP (1 μCi per reaction) at 30°C for 30 min. The reaction was terminated by adding SDS loading buffer. After the reaction, phosphate incorporation was visualized using autoradiography. After electrophoresis, the gels were stained by Coomassie Brilliant Blue as loading controls.

Supplementary Fig. 11

Normalized quantification of the EMSA gel (to Nrf2 transfection alone as the Control for each lane) of the ARE/Nrf2 EMSAs in Fig. 4k, two replications.

Supplementary Fig. 12

(a) qPCR analysis of *Sik1* and *Sik2* mRNA expression in lung tissues at 24 h following intratracheal administration of PBS or LPS, n=3/group (one-way ANOVA followed by Tukey’s post-hoc test). (b) Immunoblotting analysis of SIK1 and SIK2 in lung tissues at 24 h following intratracheal administration of PBS or LPS. β-ACTIN served as a loading control. Densitometric scanning of immunoblots was performed in which the level of a target protein was normalized against the protein level in Ctrl group, which was arbitrarily set at 1 (n=3, one-way ANOVA followed by Tukey’s post-hoc test).

Supplementary Fig. 13

293T cells were transfected with pcDNA3.1-His-Stub1 or pCMV6-Myc-Sik3 as indicated. 48 h after transfection, cells were treated with LPS (100 ng/ml) for another 12 h. Cells were then lysed and subjected to immunoprecipitation using anti-SIK3 under denaturing conditions, followed by immunoblotting with the indicated antibodies.

Supplementary Fig. 14

AT2s and AT2^Stub-/-^ cells were treated with LPS (100 ng/ml) for 12 h. Cell culture supernatants were then assayed using ELISA for TNF-α, IL-6 or IL-1β per manufacturer’s instructions (n=4, **P*<0.05, ***P*<0.01, ****P*<0.001 and *****P*<0.0001, two-way ANOVA followed by Tukey’s post-hoc test).

Supplementary Fig. 15

Minimal toxicity to normal tissues in mice treated with AAV-SIK3 (T163E). (a) A flowchart for AAV production regarding *in vivo* administration. (b) Biodistribution of GFP in lung tissues at 12 h after a single instillation of pAAV6-GFP or PBS (vehicle control). Bar=25 μm. Lower panel, assessment of GFP signals through the analysis of relative fluorescent intensity (n=4, ***P*<0.01). (c) Mouse weight measurements were performed every five days a duration of 30 days (n=5). (d) Basic blood chemistry tests in the mice treated with PBS or AAV-SIK3 (T163E) were carried out at the end of 30 days following AAV administration. (e) Representative images of H&E stained tissues from the mice after 30 days of treatment with PBS or AAV-SIK3 (T163E). No notable toxicities were identified. Bar=50 μm

Supplementary Table 1. Prediction of proteome-wide E3 ligase-substrate for STUB1 by UbiBrowser 2.0 (http://ubibrowser.bio-it.cn/ubibrowser_v3/) (partial data).

SwissProt ID (E3) Gene Symbol (E3) SwissProt ID (Substrate) Gene Symbol(Substrate) Domain_LikelihoodRatio Go_LikelihoodRatio Network_LikelihoodRatio Motif_LikelihoodRatio Confidence Score Species

Q9WUD1 Stub1 Q9CVD2 Atxn3 12.12 7.74 1.00 9.23 0.950 M.musculus

Q9WUD1 Stub1 Q9EQY0 Ern1 7.88 7.74 1.00 9.23 0.940 M.musculus

Q9WUD1 Stub1 Q9Z2E3 Ern2 7.88 7.74 1.00 9.23 0.940 M.musculus

Q9WUD1 Stub1 A2CG49 Kalrn 7.88 5.94 1.00 9.23 0.933 M.musculus

Q9WUD1 Stub1 P01108 Myc 7.88 5.94 1.00 9.23 0.933 M.musculus

Q9WUD1 Stub1 P37040 Por 12.12 8.56 3.83 1.00 0.931 M.musculus

Q9WUD1 Stub1 Q4LDD4 Arap1 7.88 5.85 3.83 2.28 0.931 M.musculus

Q9WUD1 Stub1 Q3UHJ0 Aak1 7.88 5.35 1.00 9.23 0.930 M.musculus

Q9WUD1 Stub1 Q9EQW6 Olig2 7.88 3.03 3.83 4.09 0.929 M.musculus

Q9WUD1 Stub1 P70313 Nos3 12.12 8.56 1.00 3.35 0.927 M.musculus

Q9WUD1 Stub1 O35226 Psmd4 12.12 7.74 1.00 3.35 0.924 M.musculus

Q9WUD1 Stub1 P06537 Nr3c1 3.90 8.56 1.00 9.23 0.923 M.musculus

Q9WUD1 Stub1 P19091 Ar 3.90 8.56 1.00 9.23 0.923 M.musculus

Q9WUD1 Stub1 O55098 Stk10 7.88 3.63 1.00 9.23 0.918 M.musculus

Q9WUD1 Stub1 O88898 Tp63 7.88 3.63 1.00 9.23 0.918 M.musculus

Q9WUD1 Stub1 P35761 Ttk 7.88 3.63 1.00 9.23 0.918 M.musculus

Q9WUD1 Stub1 Q0KL02 Trio 7.88 3.63 1.00 9.23 0.918 M.musculus

Q9WUD1 Stub1 Q8R3L8 Cdk8 7.88 3.63 1.00 9.23 0.918 M.musculus

Q9WUD1 Stub1 Q91Z96 Bmp2k 7.88 3.63 1.00 9.23 0.918 M.musculus

Q9WUD1 Stub1 Q9JJP2 Tp73 7.88 3.63 1.00 9.23 0.918 M.musculus

Q9WUD1 Stub1 Q9QZR5 Hipk2 7.88 3.63 1.00 9.23 0.918 M.musculus

Q9WUD1 Stub1 Q9R0G8 Nrk 7.88 3.63 1.00 9.23 0.918 M.musculus

Q9WUD1 Stub1 Q672J9 Nox3 12.12 8.56 1.00 2.28 0.915 M.musculus

Q9WUD1 Stub1 Q8VE38 Oxnad1 12.12 8.56 1.00 2.28 0.915 M.musculus

Q9WUD1 Stub1 Q02067 Ascl1 7.88 3.07 1.00 9.23 0.913 M.musculus

Q9WUD1 Stub1 O08789 Mnt 7.88 3.02 1.00 9.23 0.912 M.musculus

Q9WUD1 Stub1 O35185 Bhlhe40 7.88 3.02 1.00 9.23 0.912 M.musculus

Q9WUD1 Stub1 P35569 Irs1 7.88 3.03 1.00 9.23 0.912 M.musculus

Q9WUD1 Stub1 Q6P4S6 Sik3 7.88 3.02 1.00 9.23 0.912 M.musculus

Q9WUD1 Stub1 Q8BM47 Plekhm3 7.88 3.02 1.00 9.23 0.912 M.musculus

Q9WUD1 Stub1 Q8CHE4 Phlpp1 7.88 3.02 1.00 9.23 0.912 M.musculus

Q9WUD1 Stub1 Q9JIZ5 Tfap4 7.88 3.03 1.00 9.23 0.912 M.musculus

Q9WUD1 Stub1 Q9JKN5 Olig1 7.88 3.03 1.00 9.23 0.912 M.musculus

Q9WUD1 Stub1 Q9JM52 Mink1 7.88 3.03 1.00 9.23 0.912 M.musculus

Q9WUD1 Stub1 Q9QYC3 Bhlha15 7.88 3.02 1.00 9.23 0.912 M.musculus

Q9WUD1 Stub1 Q9R210 Tfeb 7.88 3.03 1.00 9.23 0.912 M.musculus

Q9WUD1 Stub1 O09000 Ncoa3 3.90 5.94 1.00 9.23 0.911 M.musculus

Q9WUD1 Stub1 P35583 Foxa2 3.90 5.94 1.00 9.23 0.911 M.musculus

Q9WUD1 Stub1 P39429 Traf2 12.12 7.74 1.00 2.28 0.911 M.musculus

Q9WUD1 Stub1 P51450 Rorc 3.90 5.94 1.00 9.23 0.911 M.musculus

Q9WUD1 Stub1 P53762 Arnt 3.90 5.85 1.00 9.23 0.911 M.musculus

Q9WUD1 Stub1 P58462 Foxp1 3.90 5.94 1.00 9.23 0.911 M.musculus

Q9WUD1 Stub1 Q60644 Nr1h2 3.90 5.85 1.00 9.23 0.911 M.musculus

Q9WUD1 Stub1 Q60987 Foxg1 3.90 5.94 1.00 9.23 0.911 M.musculus

Q9WUD1 Stub1 Q6NZQ4 Paxip1 1.00 5.94 3.83 9.23 0.911 M.musculus

Q9WUD1 Stub1 E9Q842 Nav2 1.00 5.85 3.83 9.23 0.910 M.musculus

Q9WUD1 Stub1 O35099 Map3k5 7.88 7.74 1.00 3.35 0.910 M.musculus

Q9WUD1 Stub1 B2RUQ2 Usf3 7.88 2.78 1.00 9.23 0.909 M.musculus

Q9WUD1 Stub1 P28028 Braf 7.88 5.94 1.00 4.09 0.907 M.musculus

Q9WUD1 Stub1 P83741 Wnk1 7.88 5.94 1.00 4.09 0.907 M.musculus

Q9WUD1 Stub1 Q80TJ1 Cadps 7.88 5.85 1.00 4.09 0.907 M.musculus

Q9WUD1 Stub1 Q61006 Musk 7.88 5.94 3.83 1.00 0.905 M.musculus

Q9WUD1 Stub1 Q8BWB6 Steap2 12.12 5.94 1.00 2.28 0.902 M.musculus

Q9WUD1 Stub1 Q3U1N2 Srebf2 7.88 5.94 1.00 3.35 0.900 M.musculus

Q9WUD1 Stub1 Q64124 Scx 7.88 5.94 1.00 3.35 0.900 M.musculus

Q9WUD1 Stub1 Q6PDH0 Phldb1 7.88 5.94 1.00 3.35 0.900 M.musculus

Q9WUD1 Stub1 Q9Z1W9 Stk39 7.88 5.94 1.00 3.35 0.900 M.musculus

Q9WUD1 Stub1 P00520 Abl1 7.88 5.35 1.00 3.35 0.896 M.musculus

Q9WUD1 Stub1 P11798 Camk2a 7.88 7.74 1.00 2.28 0.895 M.musculus

Q9WUD1 Stub1 P97343 Uhmk1 7.88 7.74 1.00 2.28 0.895 M.musculus

Q9WUD1 Stub1 Q5S006 Lrrk2 7.88 7.74 1.00 2.28 0.895 M.musculus

Q9WUD1 Stub1 Q91W69 Epn3 12.12 3.03 3.83 1.00 0.895 M.musculus

Q9WUD1 Stub1 P02340 Tp53 12.12 8.56 1.00 1.32 0.894 M.musculus

Q9WUD1 Stub1 P29477 Nos2 12.12 8.56 1.00 1.32 0.894 M.musculus

Q9WUD1 Stub1 Q61093 Cybb 12.12 8.56 1.00 1.32 0.894 M.musculus

Q9WUD1 Stub1 Q8CIZ9 Nox1 12.12 8.56 1.00 1.32 0.894 M.musculus

Q9WUD1 Stub1 P58463 Foxp2 3.90 3.63 1.00 9.23 0.892 M.musculus

Q9WUD1 Stub1 Q6P9Q4 Fhod1 1.00 3.63 3.83 9.23 0.892 M.musculus

Q9WUD1 Stub1 O88811 Stam2 12.12 3.02 1.00 3.35 0.890 M.musculus

Q9WUD1 Stub1 P51448 Rora 3.90 3.46 1.00 9.23 0.890 M.musculus

Q9WUD1 Stub1 Q02780 Nfia 3.90 3.63 3.83 2.28 0.890 M.musculus

Q9WUD1 Stub1 Q9QZB6 Nr4a3 3.90 3.46 1.00 9.23 0.890 M.musculus

Q9WUD1 Stub1 P09581 Csf1r 7.88 3.63 1.00 4.09 0.888 M.musculus

Q9WUD1 Stub1 Q61532 Mapk6 7.88 3.63 1.00 4.09 0.888 M.musculus

Q9WUD1 Stub1 O08785 Clock 3.90 3.03 1.00 9.23 0.885 M.musculus

Q9WUD1 Stub1 P43135 Nr2f2 3.90 3.02 1.00 9.23 0.885 M.musculus

Q9WUD1 Stub1 Q60750 Epha1 7.88 3.63 3.83 1.00 0.885 M.musculus

Q9WUD1 Stub1 Q61324 Arnt2 3.90 3.03 1.00 9.23 0.885 M.musculus

Q9WUD1 Stub1 Q8BUR3 Foxj3 3.90 3.02 1.00 9.23 0.885 M.musculus

Q9WUD1 Stub1 Q9DBY0 Foxp4 3.90 3.02 1.00 9.23 0.885 M.musculus

Q9WUD1 Stub1 Q9ES18 Foxj2 3.90 3.02 1.00 9.23 0.885 M.musculus

Q9WUD1 Stub1 O54751 Crx 1.00 3.03 3.83 9.23 0.884 M.musculus

Q9WUD1 Stub1 O70469 Dok2 7.88 1.48 1.00 9.23 0.884 M.musculus

Q9WUD1 Stub1 P08923 Ltk 7.88 5.94 1.00 2.28 0.884 M.musculus

Q9WUD1 Stub1 P27038 Acvr2a 7.88 5.94 1.00 2.28 0.884 M.musculus

Q9WUD1 Stub1 P54265 Dmpk 7.88 5.94 1.00 2.28 0.884 M.musculus

Q9WUD1 Stub1 Q01887 Ryk 7.88 5.94 1.00 2.28 0.884 M.musculus

Q9WUD1 Stub1 Q61234 Snta1 7.88 5.94 1.00 2.28 0.884 M.musculus

Q9WUD1 Stub1 Q99PV5 Bhlhe41 7.88 5.94 1.00 2.28 0.884 M.musculus

Q9WUD1 Stub1 Q9D3R3 Cep72 1.00 3.02 3.83 9.23 0.884 M.musculus

Q9WUD1 Stub1 Q9WUB0 Rbck1 1.00 3.02 3.83 9.23 0.884 M.musculus

Q9WUD1 Stub1 Q9WV93 Hey1 7.88 5.94 1.00 2.28 0.884 M.musculus

Q9WUD1 Stub1 Q61214 Dyrk1a 7.88 5.85 1.00 2.28 0.883 M.musculus

Q9WUD1 Stub1 Q99MQ3 Pink1 7.88 5.85 1.00 2.28 0.883 M.musculus

Q9WUD1 Stub1 A2AI05 Ndor1 12.12 8.56 1.00 1.00 0.882 M.musculus

Q9WUD1 Stub1 A2AQ92 Duox1 12.12 8.56 1.00 1.00 0.882 M.musculus

Q9WUD1 Stub1 A2AQ99 Duox2 12.12 8.56 1.00 1.00 0.882 M.musculus

Q9WUD1 Stub1 B1AS42 Cyb5rl 12.12 8.56 1.00 1.00 0.882 M.musculus

Q9WUD1 Stub1 Q3TDX8 Cyb5r4 12.12 8.56 1.00 1.00 0.882 M.musculus

Q9WUD1 Stub1 Q8BJM7 Tyw1 12.12 8.56 1.00 1.00 0.882 M.musculus

Q9WUD1 Stub1 Q8C1A3 Mtrr 12.12 8.56 1.00 1.00 0.882 M.musculus

Q9WUD1 Stub1 Q9CWR7 Steap1 12.12 8.56 1.00 1.00 0.882 M.musculus

Q9WUD1 Stub1 Q9DCN2 Cyb5r3 12.12 8.56 1.00 1.00 0.882 M.musculus

Q9WUD1 Stub1 Q9JHI8 Nox4 12.12 8.56 1.00 1.00 0.882 M.musculus

Q9WUD1 Stub1 P97471 Smad4 3.90 7.74 1.00 3.35 0.881 M.musculus

Q9WUD1 Stub1 P22091 Tal1 7.88 3.07 1.00 4.09 0.880 M.musculus

Q9WUD1 Stub1 P26687 Twist1 7.88 3.02 1.00 4.09 0.880 M.musculus

Q9WUD1 Stub1 P97793 Alk 7.88 3.07 1.00 4.09 0.880 M.musculus

Q9WUD1 Stub1 Q80US8 Mxd3 7.88 3.02 1.00 4.09 0.880 M.musculus

Q9WUD1 Stub1 Q80XI6 Map3k11 7.88 3.03 1.00 4.09 0.880 M.musculus

Q9WUD1 Stub1 Q8BWD8 Cdk19 7.88 3.02 1.00 4.09 0.880 M.musculus

Q9WUD1 Stub1 Q8C078 Camkk2 7.88 3.02 1.00 4.09 0.880 M.musculus

Q9WUD1 Stub1 Q8C6A8 Bhlhe22 7.88 3.02 1.00 4.09 0.880 M.musculus

Q9WUD1 Stub1 Q8K1R7 Nek9 7.88 3.02 1.00 4.09 0.880 M.musculus

......

Supplementary Table 2. Primers used in the current study.

| **Gene** | **Sequence** | **GenBank#** |
| --- | --- | --- |
| *Stub1* Forward | 5’-CCTGATAAGAGCCCGAGTGC-3’ | NM_019719.4 |
| *Stub1* Reverse | 5’-CACAAGTGGGTTCCGAGTGA-3’ |  |
| *STUB1* Forward | 5’-TTGGTCCCTAGACCCGGAAC-3’ | NM_001293197.2 |
| *STUB1* Reverse | 5’-GAAGAAGTGCGCCTTCACAG-3’ |  |
| *18S* Forward | 5’- CTCGCCGCGCTCTACCTACCTA-3’ | M10098.1 |
| *18S* Reverse | 5’- ATGAGCCATTCGCAGTTTCACTGTA-3’ |  |
| *Sik3* Forward | 5’-GTCCCCACTTGTCACCATGA-3’ | NM_027498.3 |
| *Sik3* Reverse | 5’-AGTGTGTGCCTTTTGGACCTA-3’ |  |
| *Sik1* Forward | 5’-ATGTCGGAGTTCAGTGCGGT-3’ | NM_010831.3 |
| *Sik1* Reverse | 5’-CACTGCAAAATTGCCTTTGCC-3’ |  |
| *Sik2* Forward | 5’-TTCAGGACACCGCATCTAGC-3’ | NM_001405014.1 |
| *Sik2* Reverse | 5’-TGCTGATTGGACAAGCCACT-3’ |  |

Supplementary Table 3. Sources of antibodies and the working dilutions that were used for the current study.

| **Peptide/protein target** | **Vendor** | **Catalog no.** | **Dilutions** |
| --- | --- | --- | --- |
| Rabbit anti-STUB1 | Proteintech (Wuhan, China) | 55430-1-AP | 1:500 (IB)  1:100 (IHC)  1:100 (IF) |
| Rabbit anti-Nrf2 | Cell signaling (Shanghai, China) | #12721 | 1:400 (IB)  1 μl/5 μg chromatin (IP) |
| Rabbit anti-β-ACTIN | Cell signaling (Shanghai, China) | #4970 | 1:3000 (IB) |
| Rabbit anti-Ub | Thermo Scientific (Shanghai, China) | PA1-187 | 1:1500 (IB) |
| Rabbit anti-Flag | Abcam (Hangzhou, China) | ab1162 | 1:2000 (IB) |
| Rabbit anti-GST | Abcam (Hangzhou, China) | ab184804 | 1:1000 (IB) |
| Rabbit anti-p-ERK | Cell signaling (Shanghai, China) | #4370 | 1:1000 (IB) |
| Rabbit anti-ERK | Cell signaling (Shanghai, China) | #4695 | 1:2000 (IB) |
| Rabbit anti-p-S/TP | Abcam (Hangzhou, China) | ab9344 | 1:500 (IB) |
| Anti-Rabbit IgG-Cy3 | Elabscience (Wuhan, China) | E-IR-R321 | 1:500 (IF) |
| Mouse anti-ERK1 | Thermo Scientific (Shanghai, China) | MA1-13041 | 1:1000 (IB) |
| Mouse anti-ERK2 | Thermo Scientific (Shanghai, China) | MA1-099 | 1:1000 (IB) |
| Goat anti-AT1α | Abcam (Hangzhou, China) | ab59018 | 1:50 (IF) |
| Mouse anti-SP-C | Santa Cruz Biotechnology (Shanghai, China) | sc-518029 | 1:100 (IF) |
| Rabbit anti-SIK3 | Cell signaling (Shanghai, China) | #39477 | 1:500 (IB)  1 μl/5 μg chromatin (IP) |
| Rabbit anti-SIK1 | Cell signaling (Shanghai, China) | #67776 | 1:1000 (IB) |
| Rabbit anti-SIK2 | Cell signaling (Shanghai, China) | #6919 | 1:1000 (IB) |
| Chicken anti-SIK3 | Abcam (Hangzhou, China) | ab211424 | 1:200 (IF) |
| Rabbit anti-HA | Sigma-Aldrich (Shanghai, China) | SAB5600116 | 1:2000 (IB) |
| Rabbit anti-His | Cell signaling (Shanghai, China) | #12698 | 1:2000 (IB) |
| Rabbit anti-Myc | Sigma-Aldrich (Shanghai, China) | SAB2103448 | 1:1000 (IB) |
| Rabbit anti-Flag | Abcam (Hangzhou, China) | ab1162 | 1:2000 (IB) |
| Rabbit anti-histone H1 | Thermo Scientific (Shanghai, China) | histone H1 | 1:1000 (IB) |
| Rabbit anti-CRTC2 | Cell Signaling (Shanghai, China) | #2587 | 1:2000 (IB) |
| Rabbit anti-pCRTC2^Ser171^ | Thermo Scientific (Shanghai, China) | BS-3415R | 1:400 (IB) |
| VECTASTAIN® Elite® ABC HRP Kit | VECTOR LABORATORIES, (Shenzhen, China) | PK-6105 | 1:500 (IHC) |
| Goat anti-Rabbit IgG-HRP | Thermo Scientific (Shanghai, China) | 31460 | 1:3000 (IB) |
| Goat anti-Mouse IgG-HRP | Thermo Scientific (Shanghai, China) | 31430 | 1:4000 (IB) |
| Donkey anti-Rabbit IgG-FITC | Thermo Scientific (Shanghai, China) | A16024 | 1:500 (IF) |
| Goat anti-Mouse IgG-FITC | MedChemExpress | HY-P80950 | 1:500 (IF) |
| Chicken anti-Goat IgG-  Alexa Fluor™ 594 | Thermo Scientific (Shanghai, China) | A-21468 | 1:500 (IF) |
| Chicken anti-Mouse IgG-  Alexa Fluor™ 594 | Thermo Scientific (Shanghai, China) | A-21201 | 1:500 (IF) |
| Goat anti-Chicken IgG-  Alexa Fluor™ 594 | Thermo Scientific (Shanghai, China) | A-11042 | 1:500 (IF) |

IHC, immunohistochemistry/immunostaining; IB, Western blotting/immunoblotting; IF, immunofluorescence; IP, immunoprecipitation.
