# Supplementary figures and images for: STUB1-induced polyubiquitination of SIK3 in alveolar type 2 epithelial cells alleviates severity and outcomes of acute lung injury
Source: Cell Death Dis. 2026 May 4;17(1):589. doi: 10.1038/s41419-026-08822-x (PMC13284247; doi:10.1038/s41419-026-08822-x)

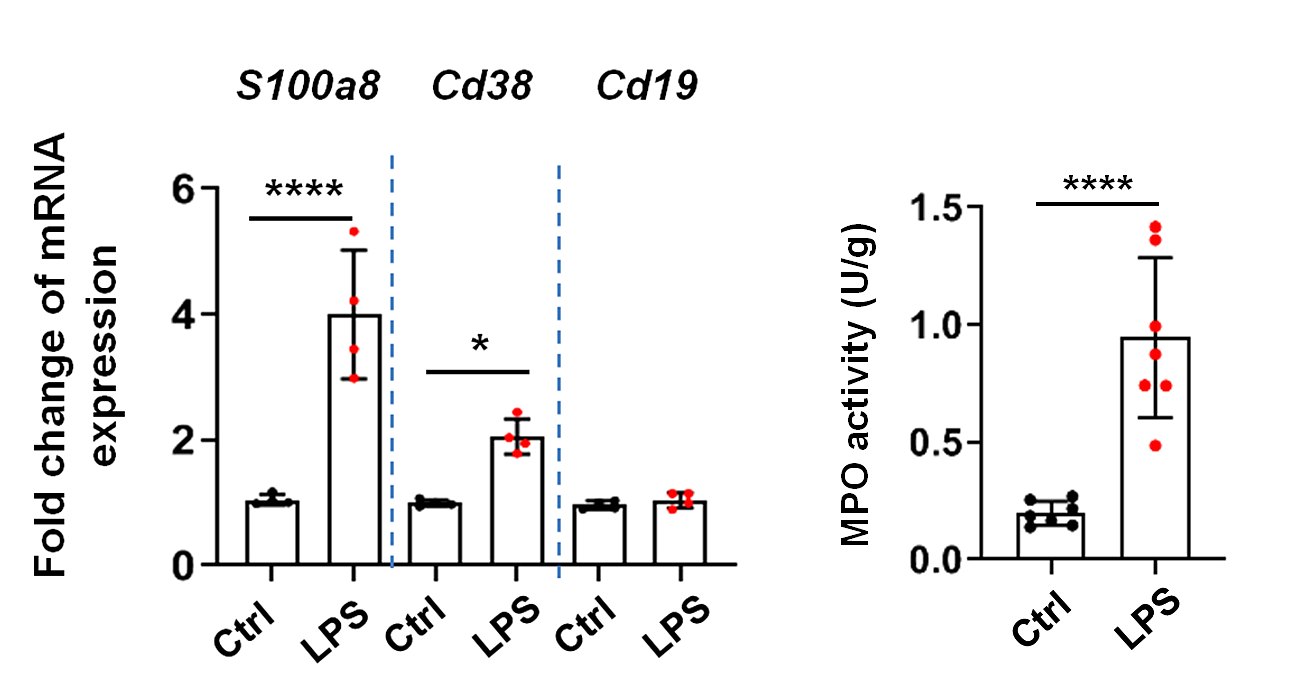

Supplement: Supplementary file 2 — Supplementary Fig. 1 [file 41419_2026_8822_MOESM2_ESM.tif]

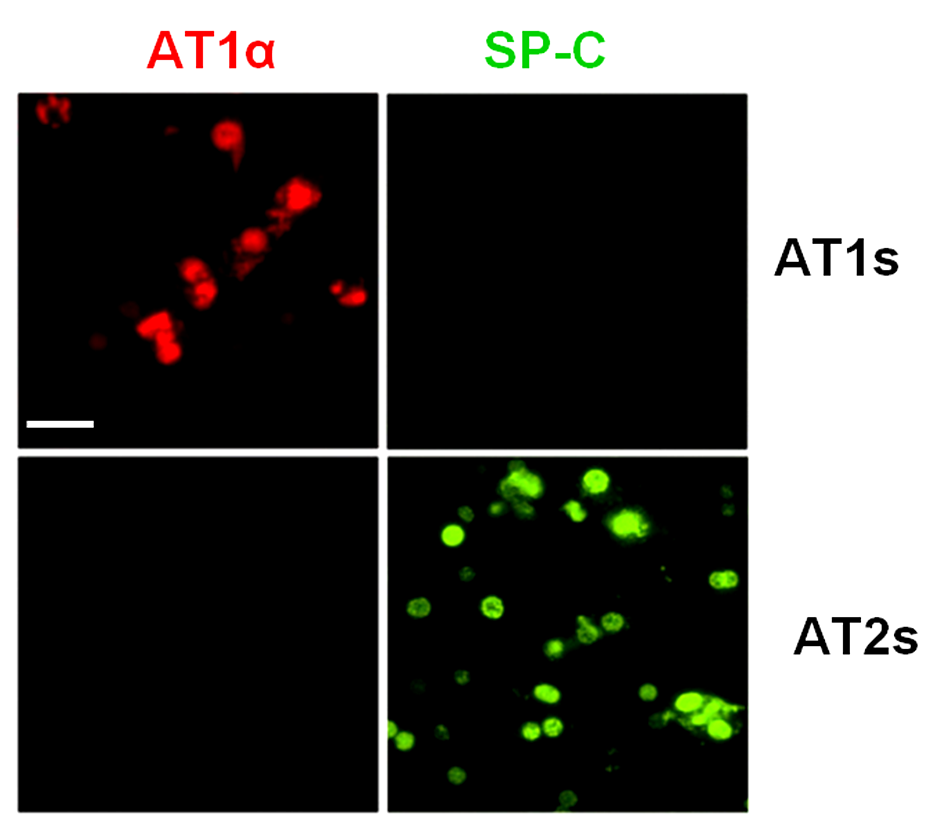

Supplement: Supplementary file 3 — Supplementary Fig. 2 [file 41419_2026_8822_MOESM3_ESM.tif]

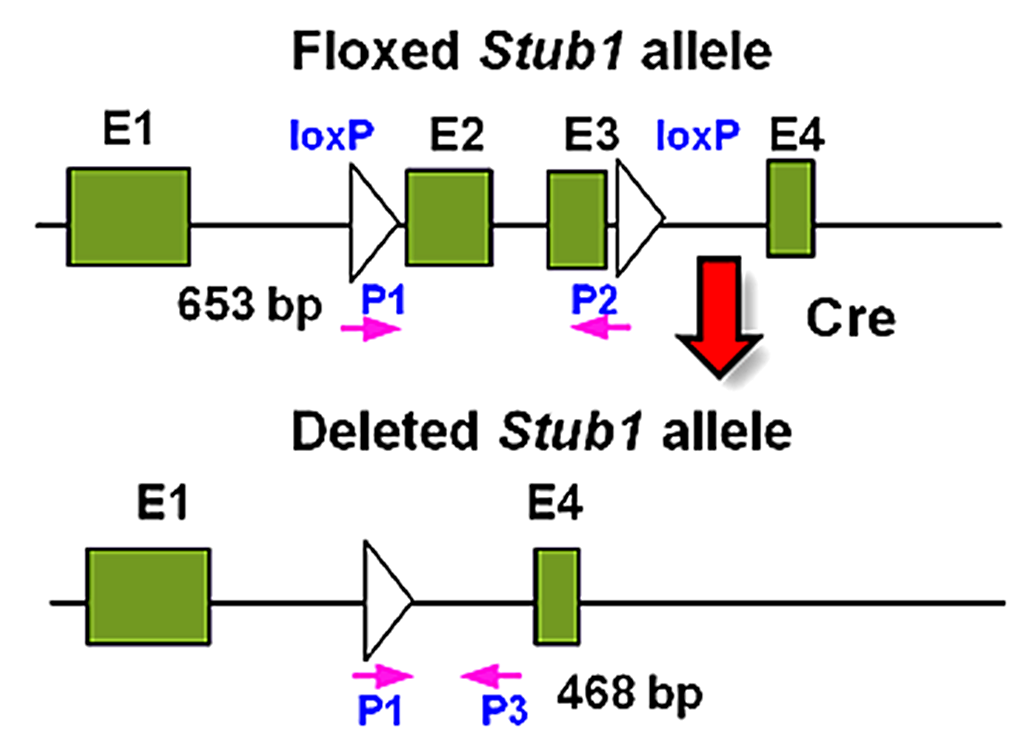

Supplement: Supplementary file 4 — Supplementary Fig. 3 [file 41419_2026_8822_MOESM4_ESM.tif]

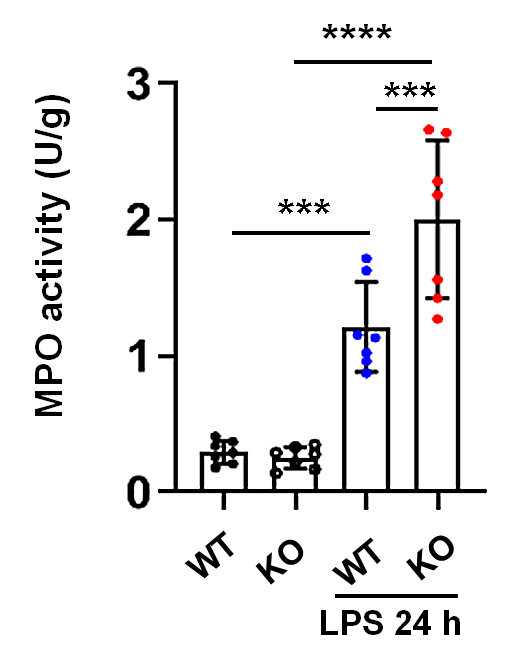

Supplement: Supplementary file 5 — Supplementary Fig. 4 [file 41419_2026_8822_MOESM5_ESM.tif]

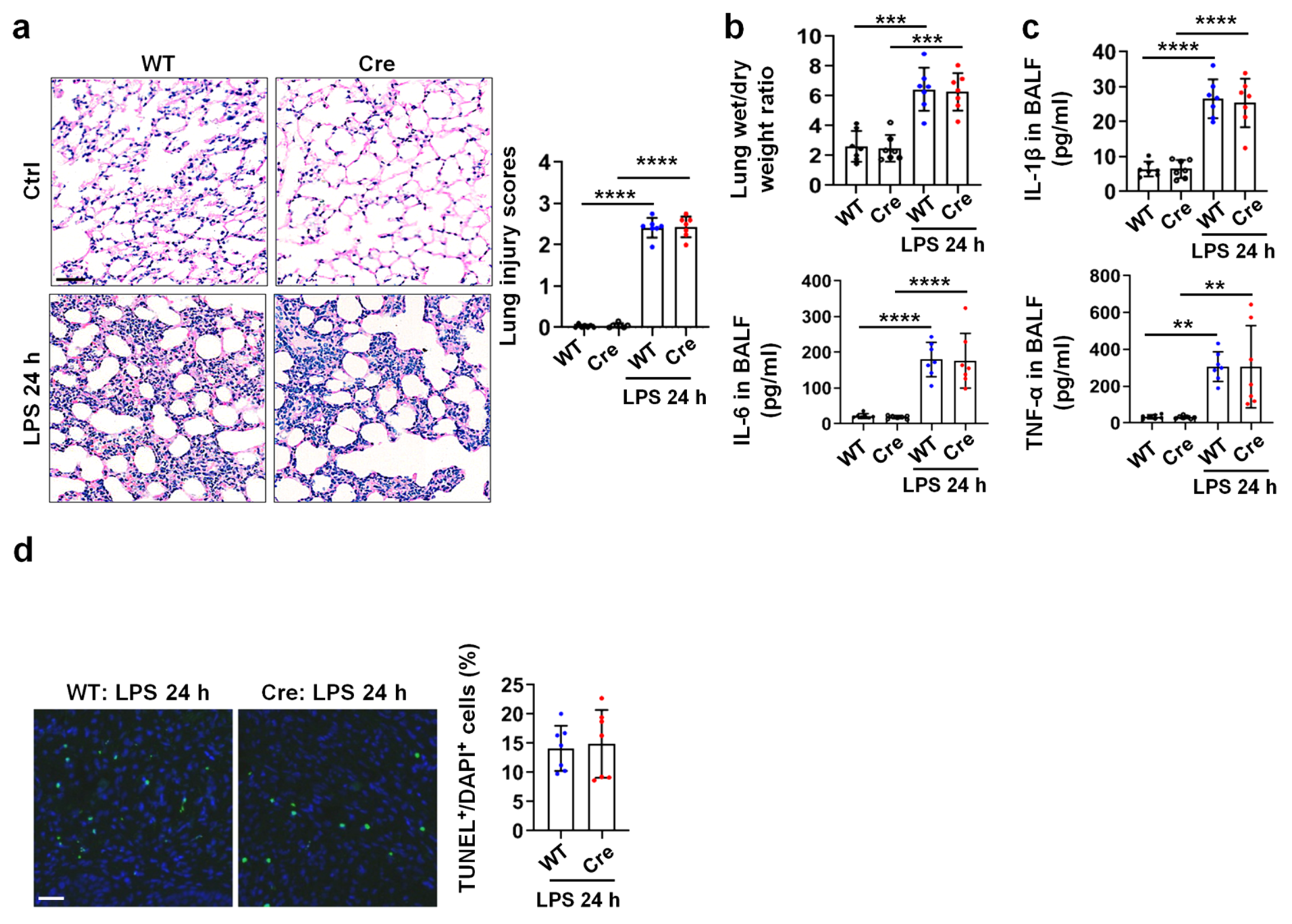

Supplement: Supplementary file 6 — Supplementary Fig. 5 [file 41419_2026_8822_MOESM6_ESM.tif]

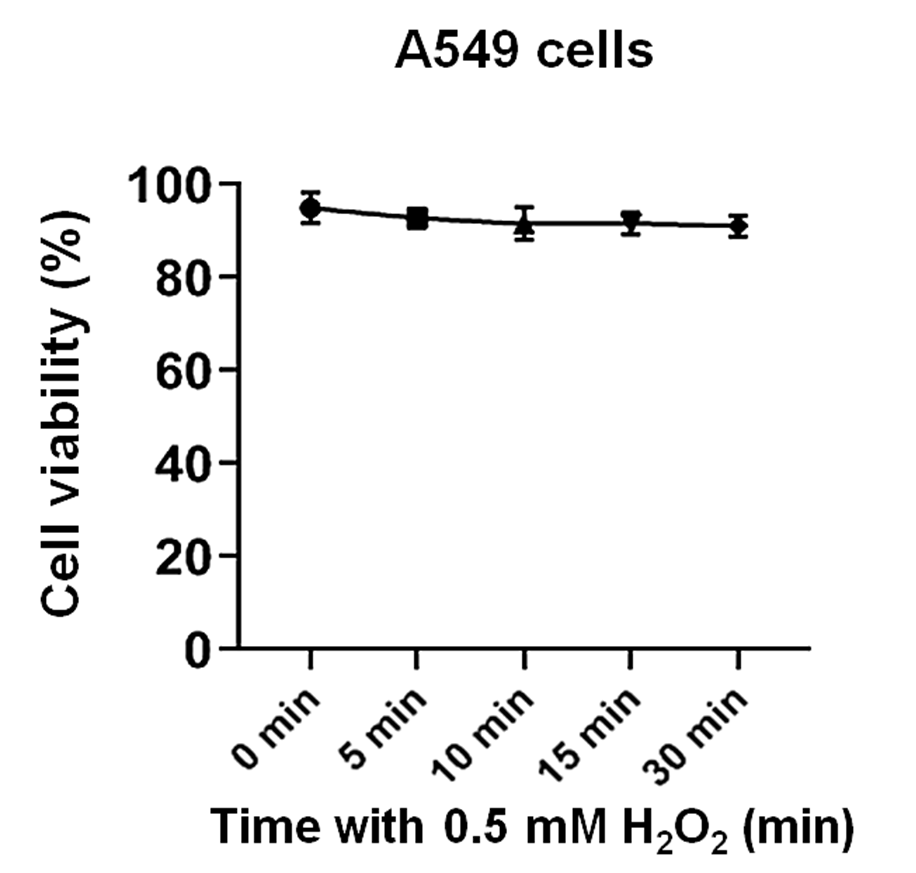

Supplement: Supplementary file 7 — Supplementary Fig. 6 [file 41419_2026_8822_MOESM7_ESM.tif]

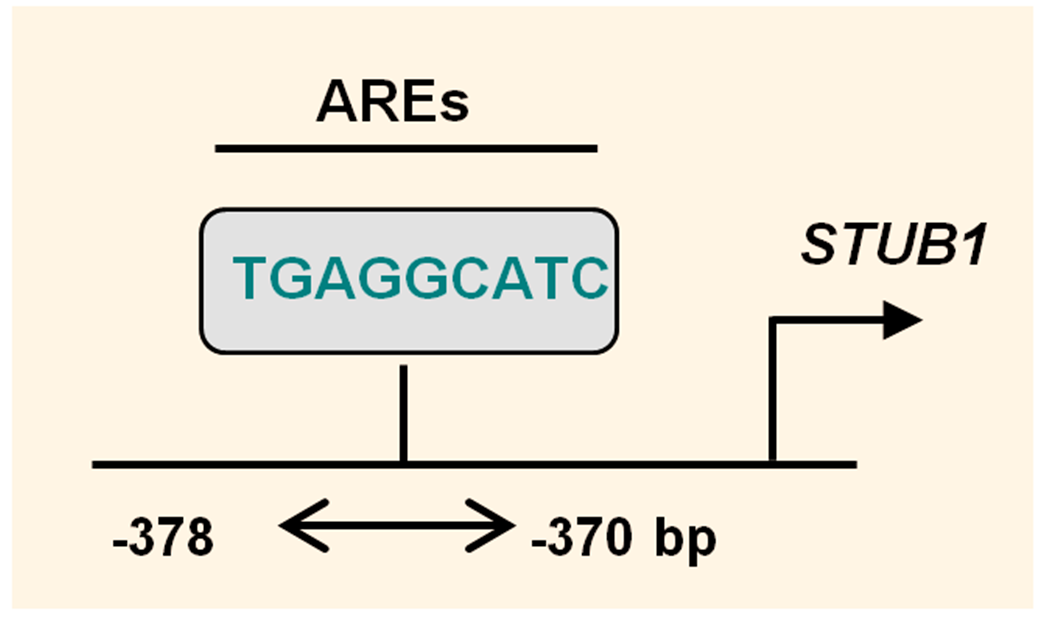

Supplement: Supplementary file 8 — Supplementary Fig. 7 [file 41419_2026_8822_MOESM8_ESM.tif]

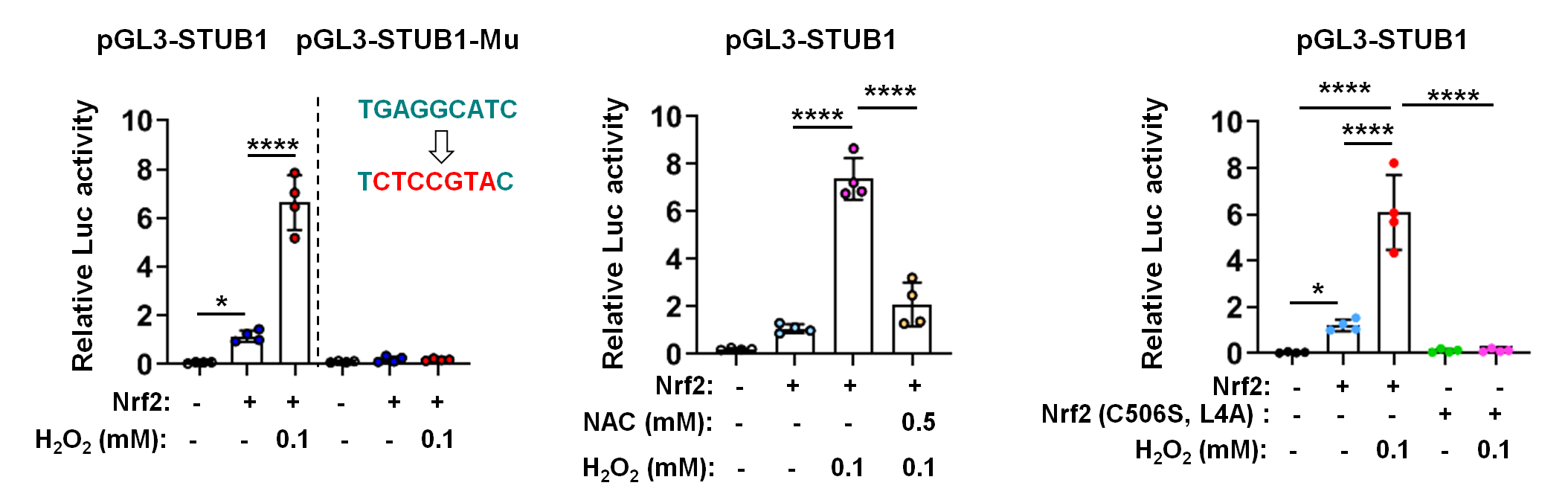

Supplement: Supplementary file 9 — Supplementary Fig. 8 [file 41419_2026_8822_MOESM9_ESM.tif]

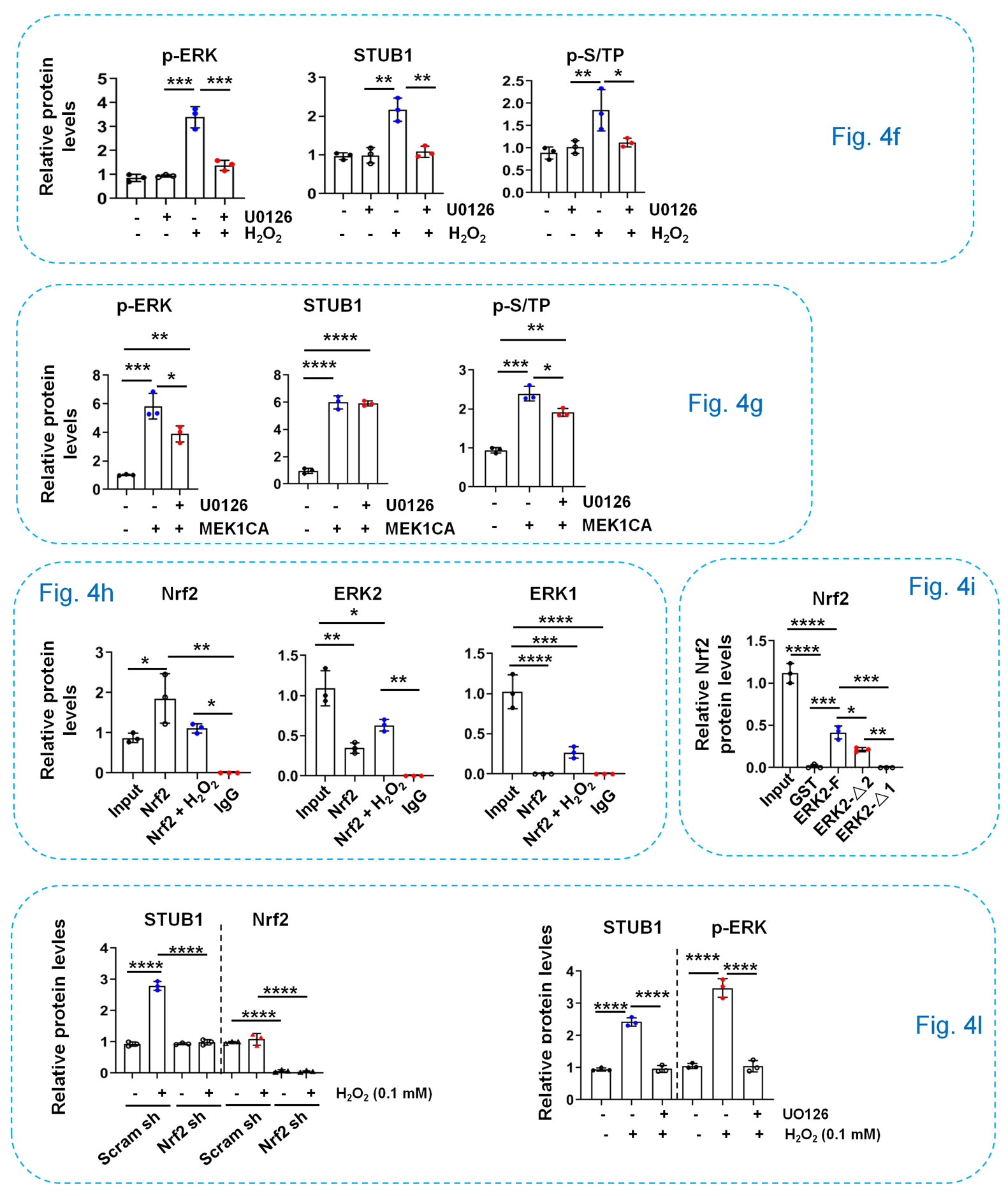

Supplement: Supplementary file 10 — Supplementary Fig. 9 [file 41419_2026_8822_MOESM10_ESM.tif]

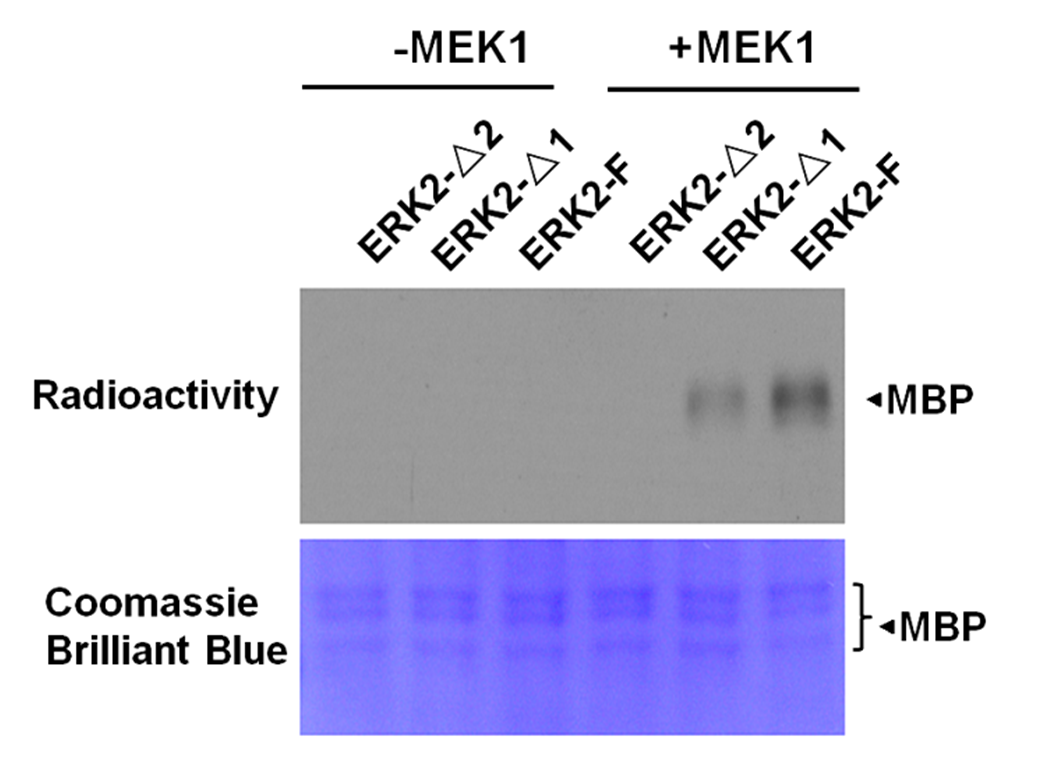

Supplement: Supplementary file 11 — Supplementary Fig. 10 [file 41419_2026_8822_MOESM11_ESM.tif]

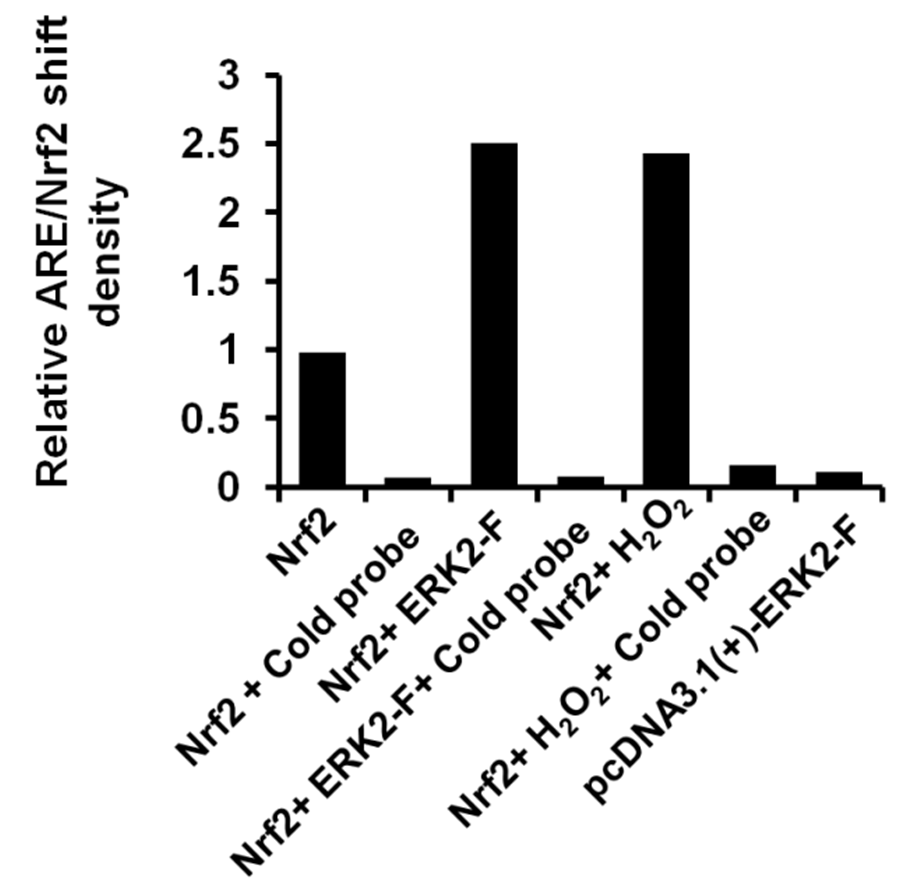

Supplement: Supplementary file 12 — Supplementary Fig. 11 [file 41419_2026_8822_MOESM12_ESM.tif]

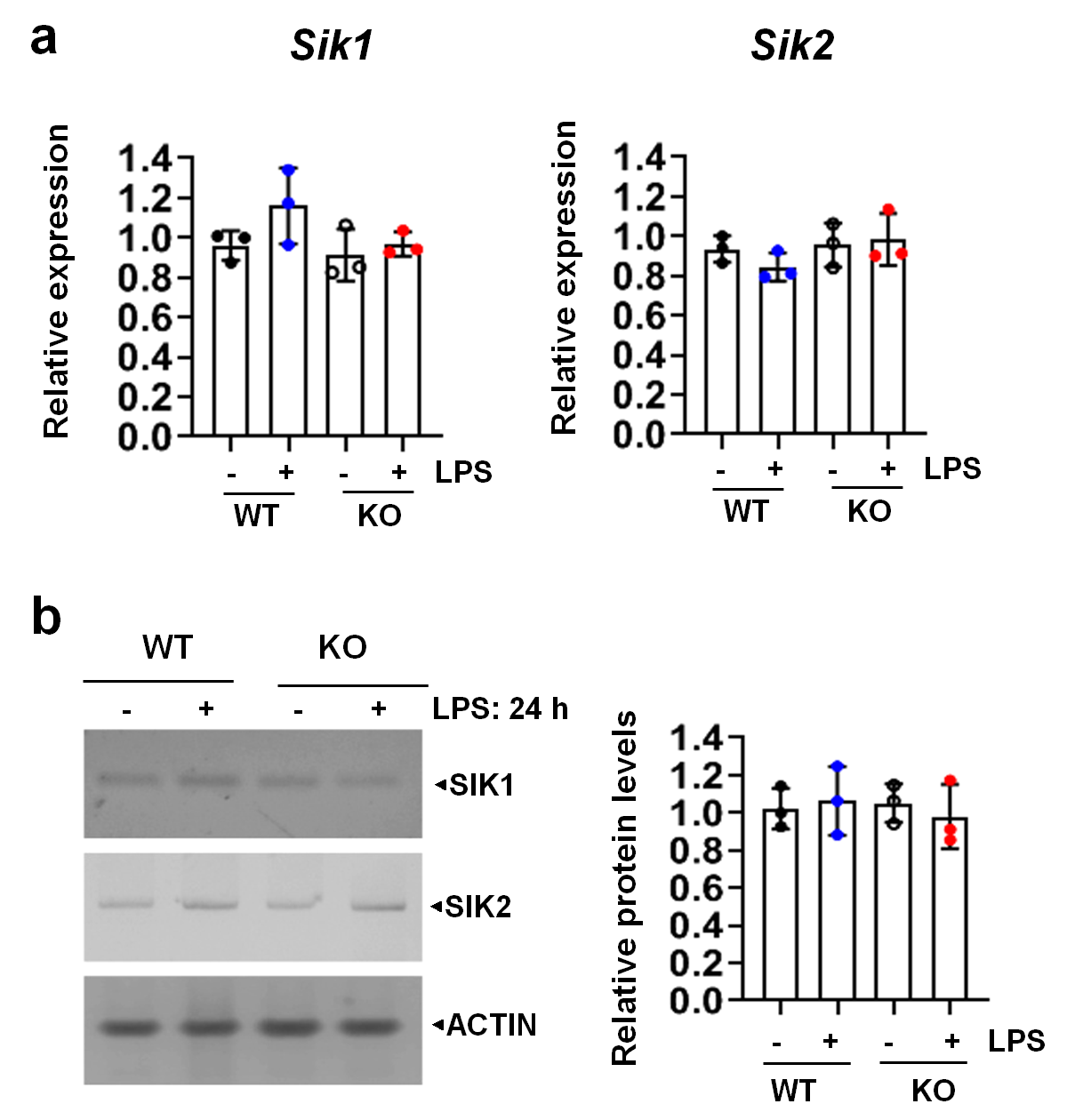

Supplement: Supplementary file 13 — Supplementary Fig. 12 [file 41419_2026_8822_MOESM13_ESM.tif]

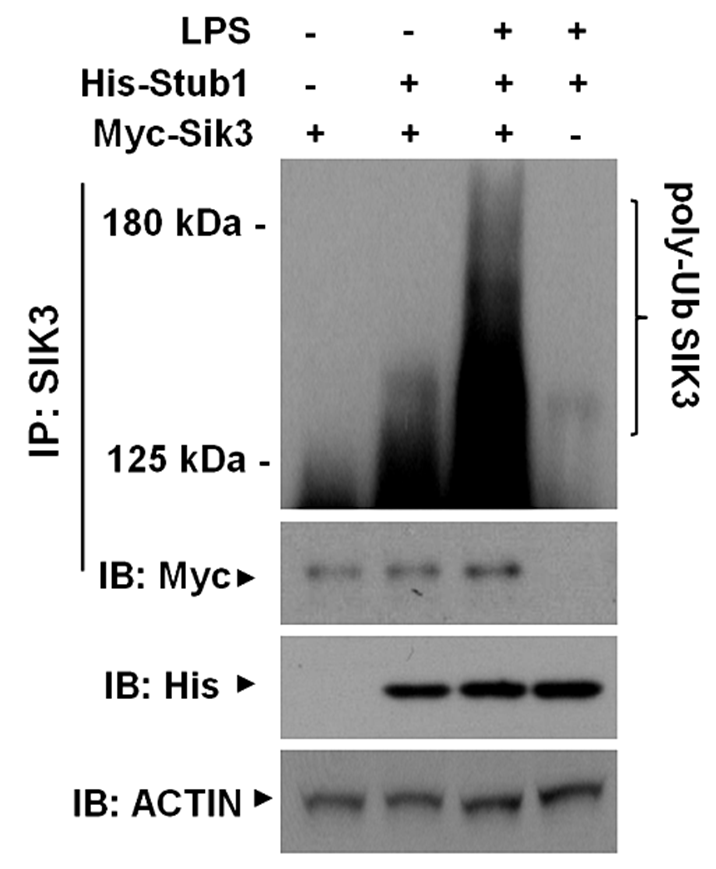

Supplement: Supplementary file 14 — Supplementary Fig. 13 [file 41419_2026_8822_MOESM14_ESM.tif]

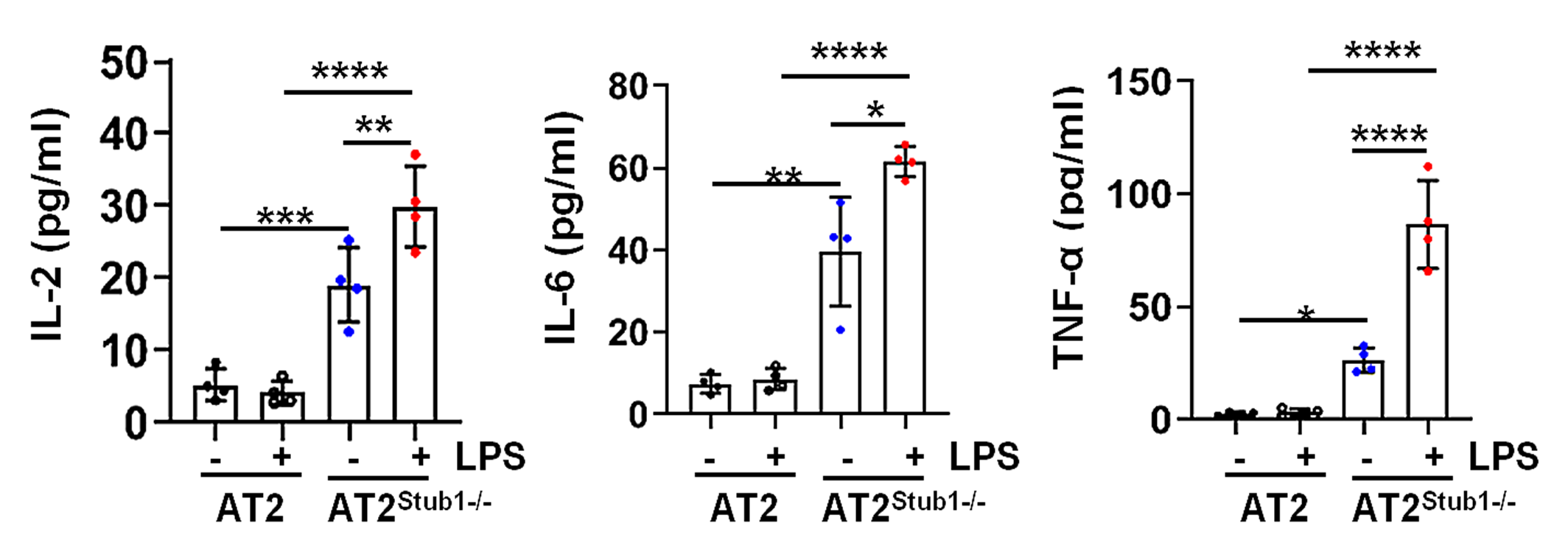

Supplement: Supplementary file 15 — Supplementary Fig. 14 [file 41419_2026_8822_MOESM15_ESM.tif]

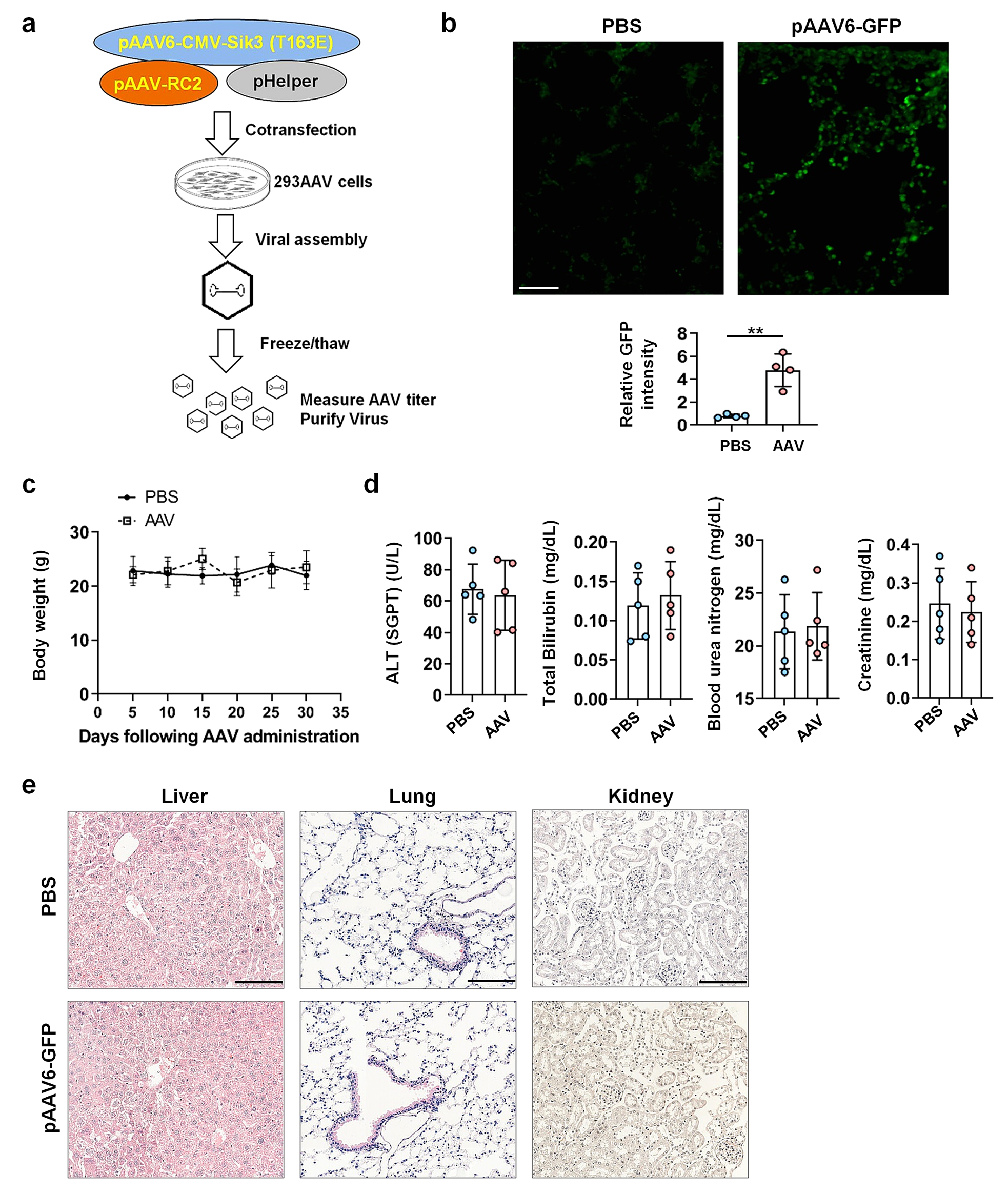

Supplement: Supplementary file 16 — Supplementary Fig. 15 [file 41419_2026_8822_MOESM16_ESM.tif]
