# Supplementary material for: STUB1-induced polyubiquitination of SIK3 in alveolar type 2 epithelial cells alleviates severity and outcomes of acute lung injury
Source: Cell Death Dis. 2026 May 4;17(1):589. doi: 10.1038/s41419-026-08822-x (PMC13284247; doi:10.1038/s41419-026-08822-x)

Uncropped full-length pictures of immunoblotting membranes. Membranes were often cut to enable blotting for multiple antibodies.

Fig. 1f

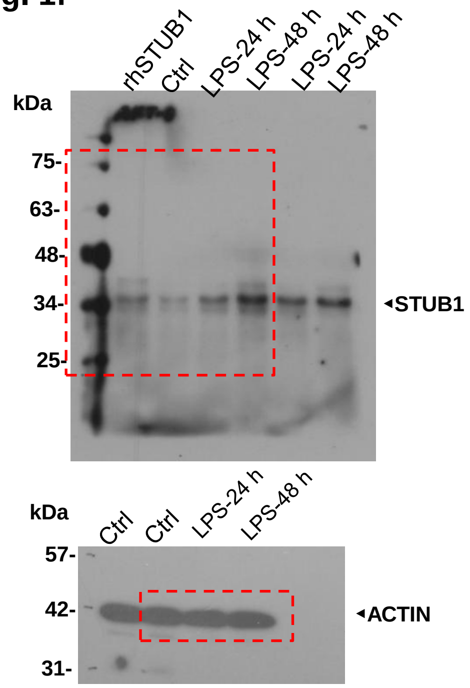

Fig. 1i

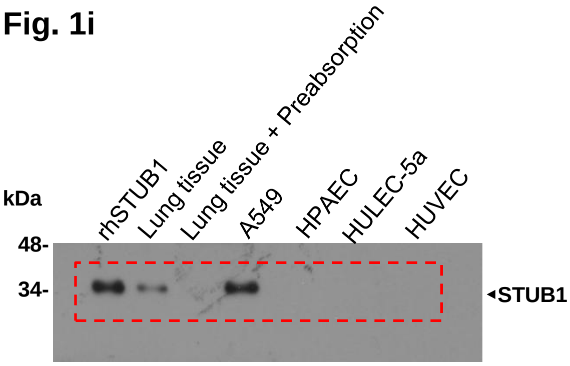

Fig. 2b

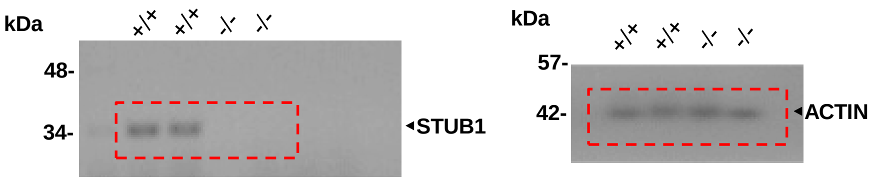

Fig. 3h

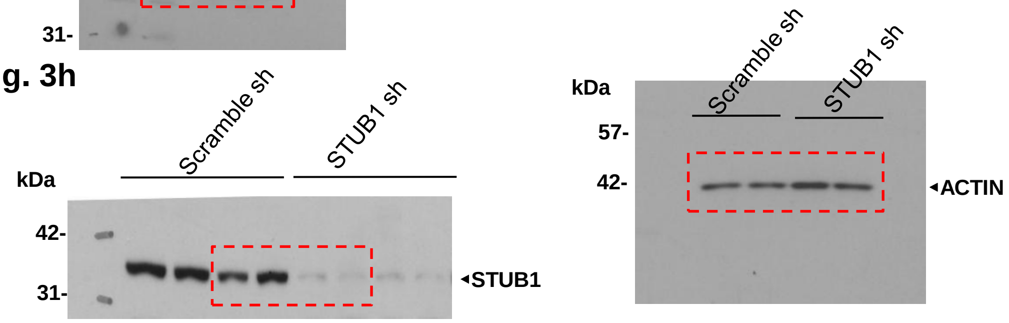

Fig. 4a

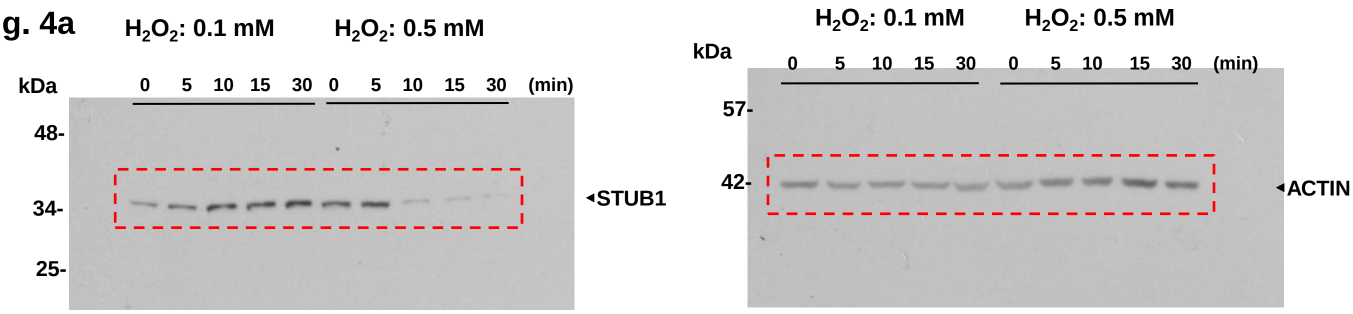

Fig. 4c

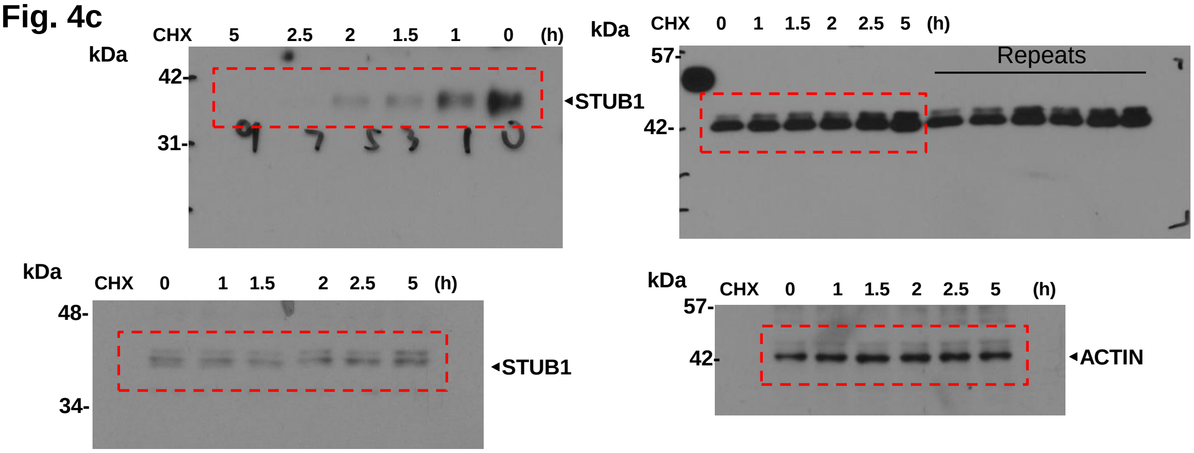

Uncropped full-length pictures of immunoblotting membranes. Membranes were often cut to enable blotting for multiple antibodies.

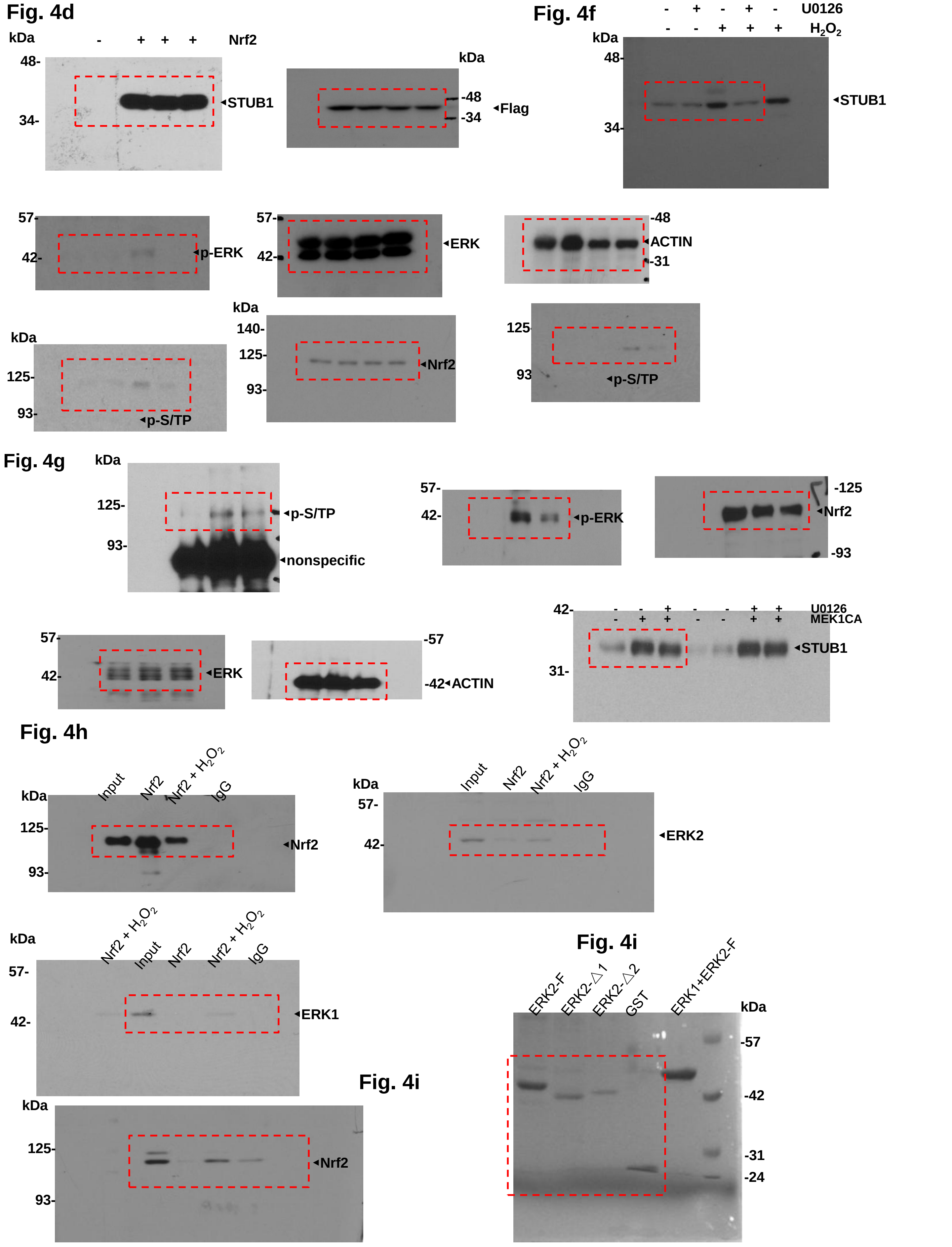

Uncropped full-length pictures of immunoblotting membranes. Membranes were often cut to enable blotting for multiple antibodies.

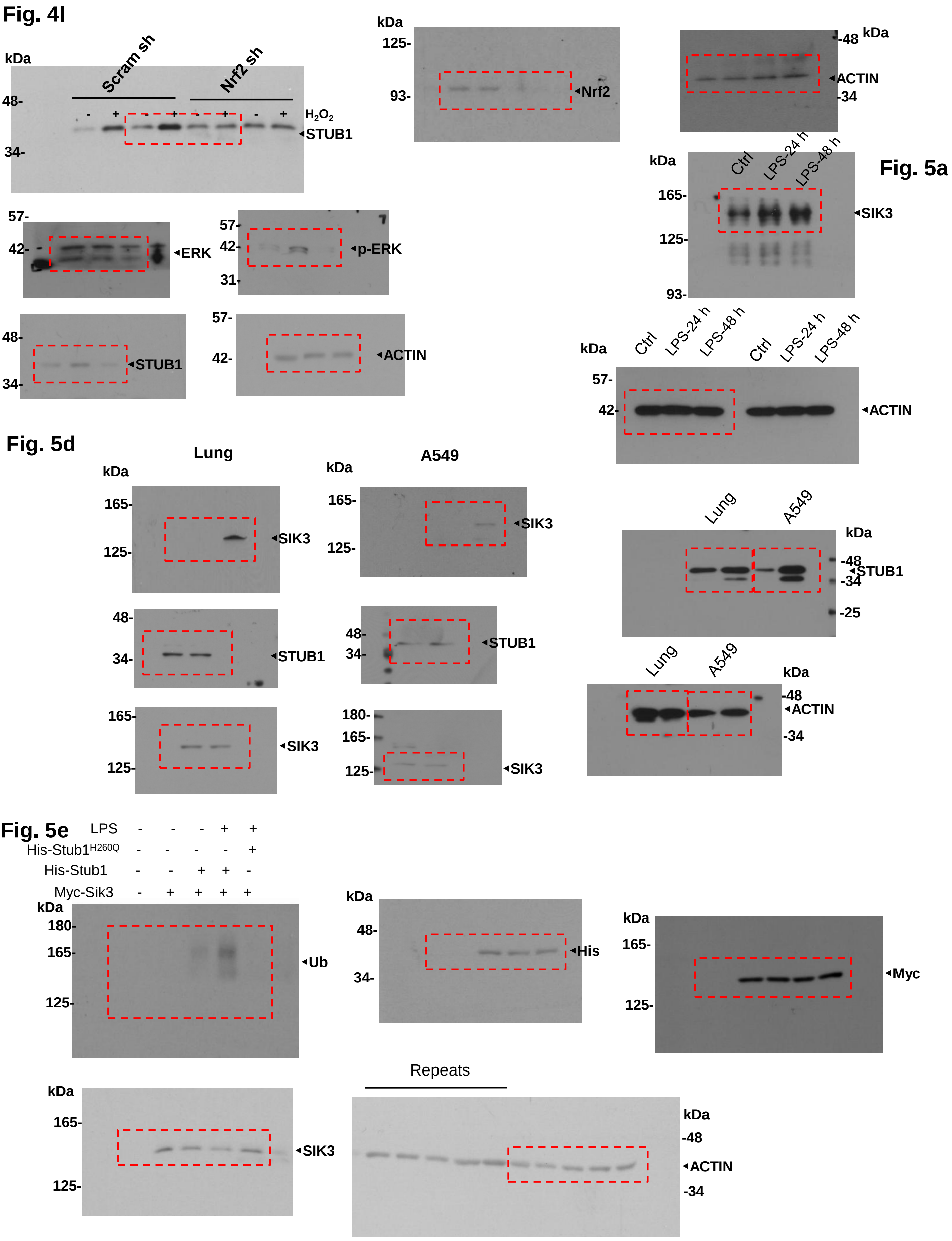

Uncropped full-length pictures of immunoblotting membranes. Membranes were often cut to enable blotting for multiple antibodies.

Fig. 5f

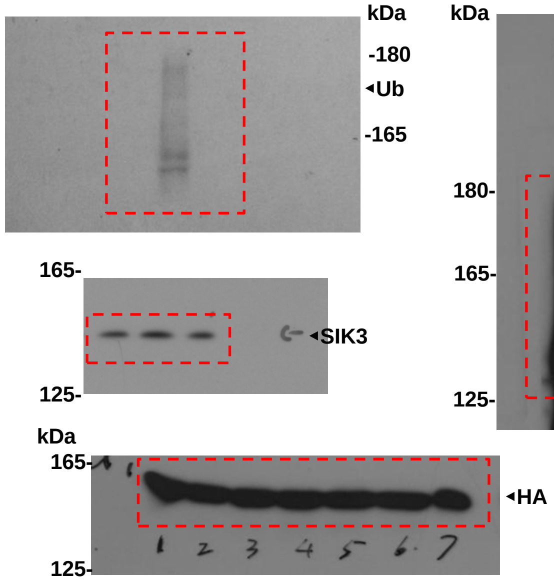

Fig. 5g

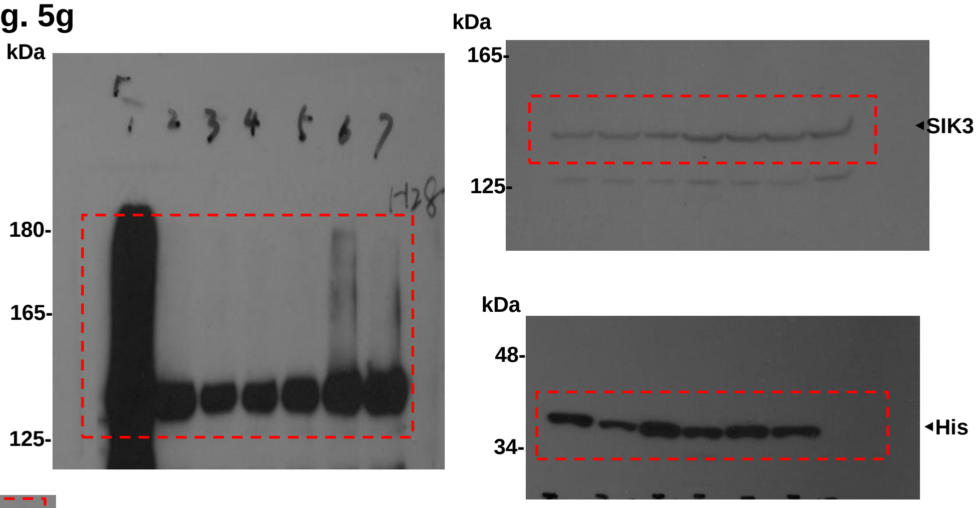

Fig. 5h

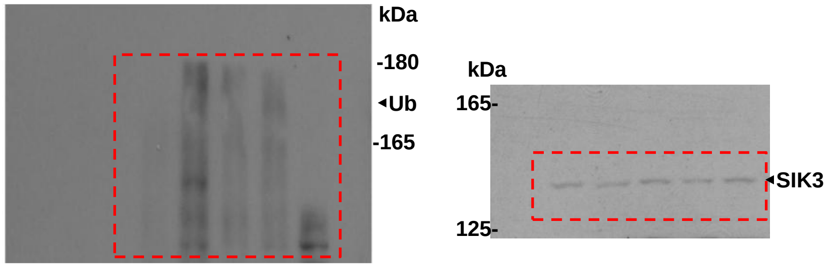

Fig. 5j

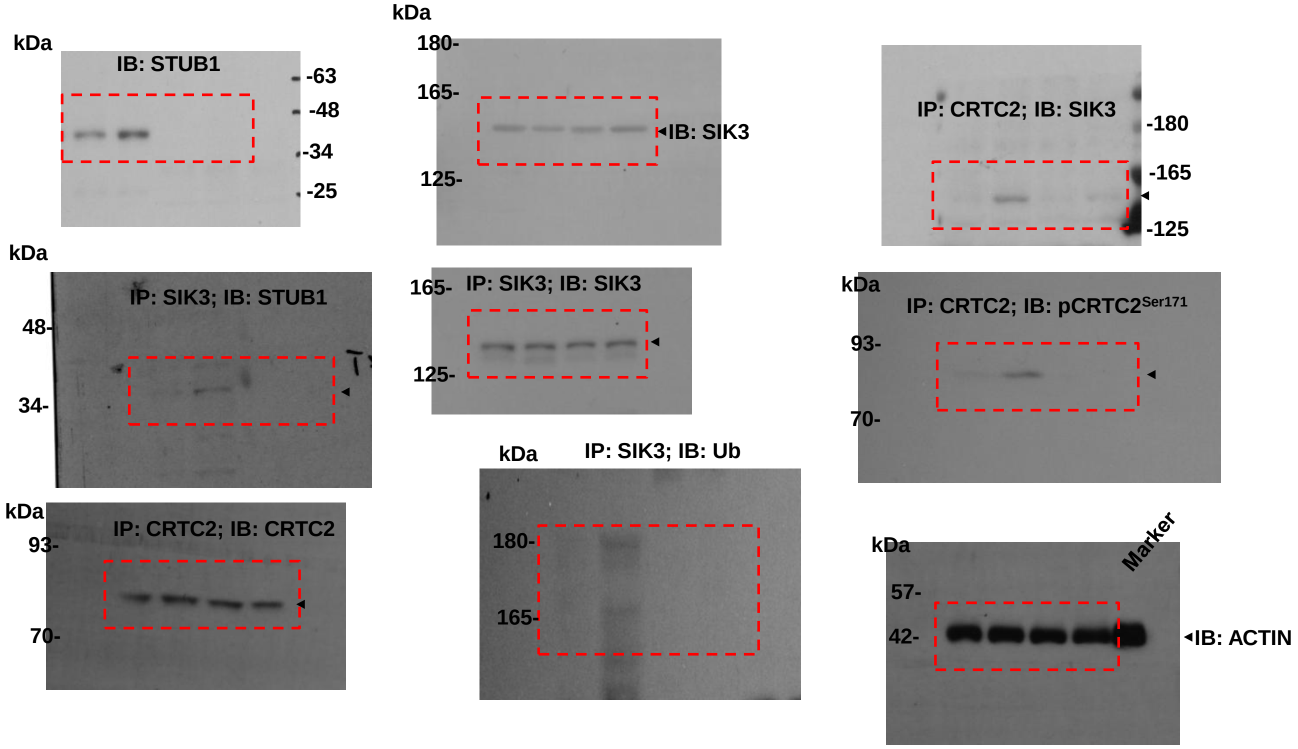

Uncropped full-length pictures of immunoblotting membranes. Membranes were often cut to enable blotting for multiple antibodies.

Fig. 5k

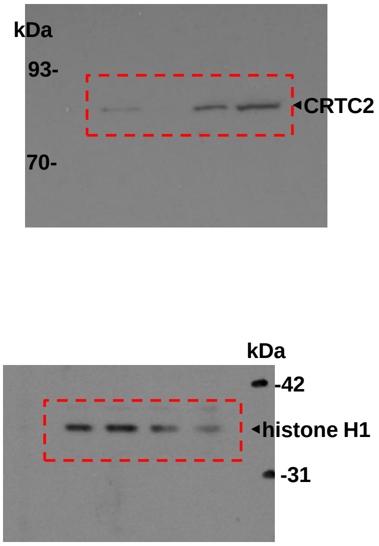

Fig. 6b

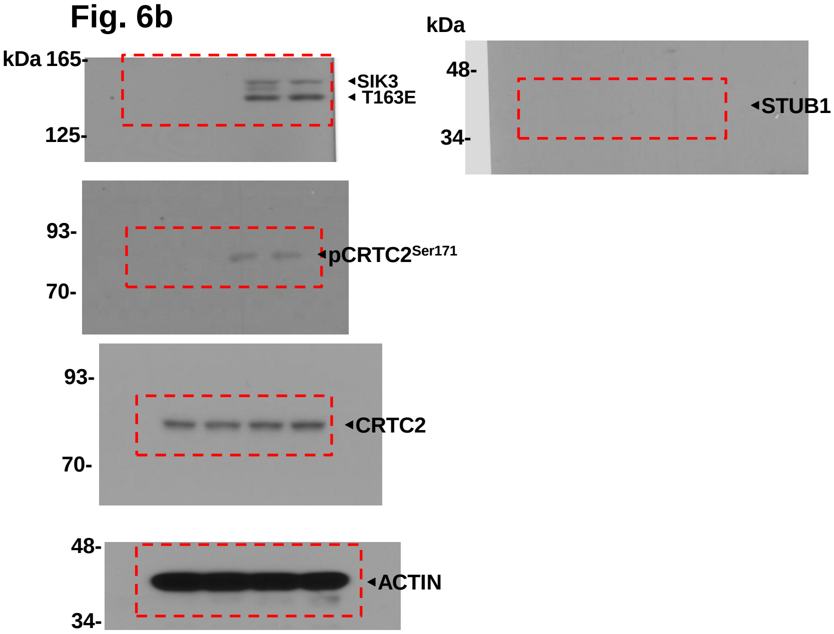

SFig. 9

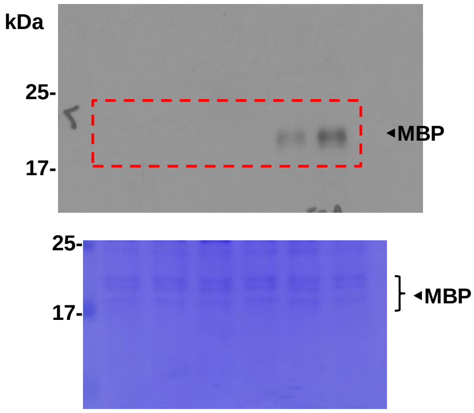

SFig. 11

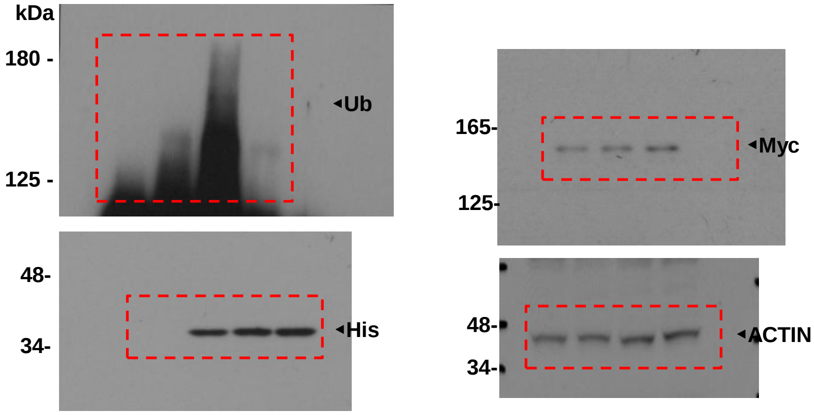

SFig. 12

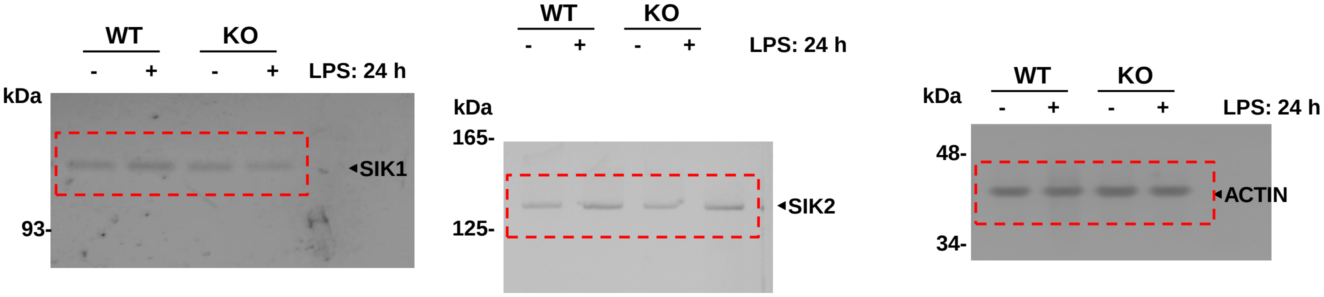

Supplement: Supplementary file 17 — Uncropped immunoblots [file 41419_2026_8822_MOESM17_ESM.pdf]
